# Genome-wide identification and drought stress-induced expression analysis of the *NHX* gene family in potato

**DOI:** 10.3389/fgene.2024.1396375

**Published:** 2024-07-11

**Authors:** Ji Yihong, Liu Zhen, Liu Chang, Shao Ziying, Zhang Ning, Suo Meiqing, Liu Yuhui, Wang Lei

**Affiliations:** ^1^ Potato Research Centre, Hebei North University, Zhangjiakou, China; ^2^ College of Agriculture and Forestry Science and Technology, Hebei North University, Zhangjiakou, China; ^3^ State Key Laboratory of Aridland Crop Science, Gansu Agricultural University, Lanzhou, China

**Keywords:** potato, NHX gene family, bioinformatics analysis, expression profile, drought stress

## Abstract

NHX proteins are transmembrane antiporters belonging to the cation/proton antiporter gene family, with a conserved Na^+^ (K^+^)/H^+^ exchange (PF00999) protein domain. NHXs play a prominent role in plant growth, development, and defense. However, the role of NHX gene family in potato (*Solanum tuberosum* L.) is yet to be known. In this study, we conducted a genome-wide analysis of the potato *NHX* gene family. A total of 25 *StNHX* family members were identified to be unevenly distributed on 10 chromosomes. The proteins ranged in length from 252 to 1,153 amino acids, with molecular masses ranging from 27516.32 to 127860.87 kD, and isoelectric points (pI) ranging from 4.96 to 9.3. Analyses of gene structures and conserved motifs indicated that *StNHX* genes in the same phylogenetic cluster are conserved. Phylogenetic analysis divided the *StNHX* genes into three subfamilies (Classes I, II, and III). Synteny analysis indicated that *StNHX* gene family Class III of NHX and all *Arabidopsis thaliana NHX*s shared a close evolutionary relationship. Analysis of *cis*-acting elements in the upstream 1,500 bp promoter region of potato *NHX* genes showed that these genes could be regulated by light, stress, and hormones such as abscisic acid and gibberellic acid. Protein-protein interaction network analysis indicated that StNHX proteins may participate in the regulation of potato growth and stress response. Besides, To determine a potential role of these genes in tissue development and drought response, we analyzed the RNA-seq data of different DM potato tissues. The results showed that *NHX* genes exhibited distinct tissue-specific expression patterns. We further examined the expression patterns of *StNHX* in different tissues (leaves, roots, shoots, tubers, stolons, and flowers) during the flowering stage in ‘Jizhangshu NO.8.’ potato. The qRT-PCR results further confirmed the importance of *StNHX* genes in potato plant growth and development. We further analyzed the RNA-seq data (DM potato) under different abiotic stresses (salt, drought, and heat), and found that the expression of *StNHX* genes was induced under abiotic stress. qRT-PCR analysis of shoots and roots of ‘Jizhangshu NO.8.’ potato treated for 0, 6, 12, and 24 h with 15% PEG6000 confirmed that the 25 *StNHX* genes are involved in the response to drought stress in potato. The results of this study may be useful for selecting appropriate candidate genes for the breeding of new drought-tolerant potato varieties. Furthermore, this study lays a foundation for prospective analysis of *StNHX* gene functions.

## 1 Introduction

In plants, transmembrane NHX antiporters, which belong to the cation/proton antiporter-1 gene family, contain a conserved Na^+^ (K^+^)/H^+^ exchange (PF00999) protein domain (Ayadi et al., 2020). In order to avoid cellular damage and maintain normal physiological functioning, According to the subcellular localization of its family members, NHXs can be divided into three classes: vacuolar membrane-localized (Vac-class), endosomal membrane-localized (Endo-class), and plasma membrane-localized (PM-class) (Orlowski and Grinstein, 2007). The model plant *A. thaliana* contains 8 *NHX* gene family members (*AtNHX1*-*AtNHX8*), including six intracellular *NHX*s (Vac-class: *AtNHX1*-*AtNHX4*; Endo-class: *AtNHX5* and *AtNHX6*) and two plasma membrane *NHX*s (*AtNHX7* and *AtNHX8*) (Yamaguchi et al., 2003). In saline or low water environments, the Vac-class *NHX*s (*AtNHX1*-*AtNHX4*) have the ability to both provide energy through the transmembrane proton gradient formed by vacuolar membrane-bound H^+^-ATPase or H^+^-PPase, and mediate Na^+^ regionalization to minimize cellular damage and stabilize osmotic pressure (Rausch et al., 1996; Apse et al., 1999; Gaxiola et al., 1999; Blumwald, 2000; Blumwald et al., 2000; Yokoi et al., 2002; Li et al., 2009; Liu et al., 2010). The End-class *NHX*s (*AtNHX5* and *AtNHX6*) are primarily localized to the endosomal compartments, including Golgi bodies and prevacuolar compartments, where they primarily regulate Na^+^ (K^+^), pH balance, and potassium nutrition (Gaxiola et al., 1999; Bassil et al., 2011; Bassil et al., 2011; Wu et al., 2016). The plasma membrane *NHX*s (*AtNHX7* and *AtNHX8*) participate in cytoplasmic Na^+^ efflux metabolism, which is the first barrier against salt ion toxicity in plants (Yamaguchi et al., 2003; An et al., 2007). *NHX* antiporters can expel Na^+^ (K^+^) out of the cell or partition Na^+^ (K^+^) into vacuoles to maintain a low cellular Na^+^ (K^+^) concentration. *NHX* antiporters have the ability to regulate cell turgor, maintain cell Na^+^ (K^+^) concentration, prompt cells to absorb water to maintain osmotic balance, regulate intracellular pH, and improve drought and salt tolerance (Elias et al., 2012; Pardo et al., 2006; Wang et al., 2014; Wang et al., 2015; Yamaguchi et al., 2001; Apse et al., 1999). The *NHX* genes have been identified and characterized in several plant species, including *Arabidopsis thaliana* (Gaxiola et al., 1999), rice (Atsunori et al., 2004), maize (Zorb et al., 2005), *Brassica napus* (Wang et al., 2003), apple (Li et al., 2010), *Gossypium hirsutum* (Li et al., 2010), banana (Xu et al., 2021), cotton (Akram et al.,. 2020), Tomato (Cavusoglu E et al., 2023), wheat (Sharma et al., 2023) among others. To date, many gene sequences encoding salt tolerance proteins in plants have been developed (Mehta et al., 2021; Cavusoglu et al., 2023). The functions of *NHX* family genes have also been widely studied. For example, applications of 200 mmol L^−1^ NaCl, 10% PEG, and 100 mmol L^−1^ ABA were found to improve the transcription level of *PbNHX1* in the leaves of *Pyrus betulaefolia* (Liu et al., 2018). The overexpression of *OsNHX1* can improve the salt tolerance of transgenic rice (Zorb et al., 2005). Genome-wide screening of *O. sativa Indica* subspecies revealed sixteen NHX orthologous, and these were confirmed to be Na+/H+ exchangers, under drought stress, the expression of *OsNHX7* and *OsNHX16* was significantly higher in transgenic rice leaves than in those of wild type plants (Khare et al., 2021). Guo Huimin et al. (Guo et al., 2012) showed that overexpression of *NnNHX1* improved the salt tolerance of transgenic tobacco, with higher expression resulting in greater salt tolerance. Transgenic corn strains overexpressing *SsNHX1* exhibited improved seed germination rate and decreased physiological damage when exposed to a 1% (w/v) NaCl solution (Huang et al., 2018). *A*. *thaliana* overexpressing *AtNHX1* could sustain growth and development in 200 mmol/L NaCl solution (Apse et al., 1999). After utilizing PCR to clone *NHX1* from wheat, functional analysis indicated that wheat *NHX1* could influence cold tolerance by regulating the expression of reverse transporters (Brini et al., 2005). Overexpression of *ZxNHX* and *ZxVP1-1* can enhance the ion regionalization ability of transgenic beet, thus improving drought tolerance (Wu et al., 2017). Furthermore, silencing *ZxNHX* leads to the downregulation of *ZxAKT1* and *ZxSKOR*, the overaccumulation of K^+^ in the tissues of *ZxNHX*-RNAi plants, and decreased drought tolerance (Yuan et al., 2015). Compared with the wild type, the activity of antioxidant enzymes in transgenic plants was enhanced, the degree of membrane lipid peroxidation was reduced, and the expression of abiotic stress-related genes was significantly upregulated after mannitol treatment (Pardo et al., 2021). The overexpression of *NnNHX1* from lotus increased salt tolerance in tobacco (Guo et al., 2012). The GhNHX1 gene plays an important role in salt stress (Wu et al., 2004). Finally, Wang et al. (2016) found that the expression of *IbNHX2* was positively correlated with the drought tolerance of transgenic sweet potato (*Ipomoea batatas*) plants (Wang et al., 2016). These studies demonstrate that overexpression of *NHX* could improve the drought tolerance of transgenic plants.

Potato (*Solanum tuberosum L.*; Solanaceae), originating from the Andean regions of Peru and Bolivia (Spooner et al., 2005), is an annual crop cultivated worldwide (Xu et al., 2011), and is the fourth most important food crop after wheat, rice, and maize (Visser et al., 2009; Zhang et al., 2017). Drought is one of the primary environmental factors limiting the productivity and growth of potato plants. Potato is very sensitive to drought, and yield reductions occur even at moderate soil moisture deficits (Gervais et al., 2021; Hill et al., 2021). In this study, we applied bioinformatics to identify 25 *NHX* gene family members across the whole potato genome. We also studied the chromosomal locations, gene duplication events, evolutionary history, and promoter elements associated with each identified *NHX* gene. Furthermore, the expression levels of NHX genes in various tissues/organs and under abiotic stress cinditions was also analyzed using publicly available potato RNA-sequencing datasets in double monoploid (DM) potato. Quantitative real-time PCR (qPCR) was used to further analyze the *StNHX* expression patterns, after which the key gene related to tissue-specific expression and drought tolerance were identified. Overall, these findings should inform the further characterization of *StNHXs* and provide valuable information for functional elucidation of these genes in potato.

## 2 Materials and methods

### 2.1 Identification of *NHX* genes in potato

The genome sequences, protein sequences, and coding sequences (CDS) of potato (Stuberosum_448_v4.03) were obtained from the Potato Genome Sequencing Consortium (PGSC; http://solanaceae.plantbiology.msu.edu/pgsc_download.shtml). In the Pfam database (http://pfam.xfam.org/), the Hidden Markov Model (HMM) profile for the *NHX* conserved domain (PF00999) was downloaded as the search model (Finn et al., 2016). HMMER 3.1 (http://hmmer.org/download.html), with default parameters, was used to obtain candidate genes containing the conserved domain (Finn et al., 2011). SMART (http://smart.embl-heidelberg.de) was used to confirm the existence of the domain, and redundant sequences without *NHX* family protein domains were removed (Letunic et al., 2015).

### 2.2 Sequence and structural characteristics of *StNHX* family members

The characteristics of StNHX proteins, including the number of amino acids, molecular weight (MW), and theoretical isoelectric point (pI), were estimated using Expasy (https://web.expasy.org/compute_pi/) (Bjellqvist et al., 1993; Gasteiger et al., 2005). Subcellular localization of proteins was predicted using CELLO (http://cello.life.nctu.edu.tw/) (Yu et al., 2006). StNHX protein motifs were analyzed using MEME (https://meme-suite.org/meme/), with a maximum of 20 motifs (Bailey et al., 2009). The exon-intron structures of *StNHX* genes were examined using the Gene Structure Display Server (GSDS; http://gsds.gao-lab.org/index.php) (Hu et al., 2014) via alignment of the CDSs with their corresponding genomic DNA sequences. The GSDS was also used to graphically display the structure of *StNHX* genes.

### 2.3 Chromosome localization and gene duplication events of *StNHX* family members

MapChart software was used to create the chromosomal position map and examine the relative distance of *StNHX* genes (Voorrips, 2002). Gene duplication events were analyzed with MCScanX (Yu et al., 2012). Tandem repeats were determined according to two conditions: 1) the length of the short sequence covers more than 70% of the length of the long sequence; 2) the similarity of the two aligned sequences is greater than 70% (Yang et al., 2008; Zheng et al., 2020). Specifically, tandem repeats refer to two genes located in the same chromosomal segment, where the distance is less than 100 kb and the number of genes between them is less than or equal to five (Wang et al., 2010).

### 2.4 Evolutionary analysis and classification of *StNHX* family members

AtNHX protein sequences were obtained from The Arabidopsis Information Resource (TAIR; http://www.arabidopsis.org). MEGA7.0 was used to construct a root-free phylogenetic tree using the maximum likelihood (ML) method and 1,000 iteration bootstraps (Kumar et al., 2016).

### 2.5 Cis-acting element prediction

The *StNHX* gene structure was analyzed, and the gene structure map was drawn based on the gene location information from the whole genome GFF annotation file. The 1,500 bp upstream promoter sequences were extracted in batches by perl script. The *StNHX* gene promoter sequences (1,500 bp upstream of the ATG translation start codon) were used to predict cis-acting elements using PLANTCARE (http://bioinformatics.psb.ugent.be/webtools/plantcare/html/) (Lescot et al., 2002).

### 2.6 Protein-protein interaction network analysis and synteny analysis

Network analysis of protein-protein interactions (PPIs) among all identified *StNHX* genes was performed using STRING v11.5 (https://string-db.org/), with a combined score of ≥0.700 (high confidence) (Szklarczyk et al., 2017). The interaction network was visualized by Cytoscape v3.9.1 (Shannon et al., 2003). Repeated events in potato genes were analyzed by MCScanX (Yupeng et al., 2012).

### 2.7 RNA-seq data analysis

To study the expression of *StNHX* genes in different tissues (leaves, roots, shoots, callus, tubers, sepals, stamens, stolons, flowers, petioles, petals, carpels, and fruit) and under different stress conditions (mannitol, salt, and high temperature), publicly-available transcriptomic data of DM potato were downloaded from the PGSC (accession no. SRA030516) (Xu et al., 2011). Gene expression was visualized using TBtools (Chen et al., 2018).

### 2.8 Plant materials and experimental treatments

Sterile tissue cultured seedlings of ‘Jizhangshu NO.8.’ potato were grown for 25 days at 22°C and with 16 h light per day. To simulate drought stress, experimental seedlings were treated for 0, 6, 12, and 24 h with 15% PEG6000. Each treatment consisted of three biological replicates. Subsequently, shoot and root tissues were collected and frozen in liquid nitrogen, and stored at −80°C until futher use.

Potatoes (‘Jizhangshu NO.8.’) were field-grown at Hebei North University, China. During the flowering stage, tissue samples (flowers, roots, stems, leaves, tubers, and stolons) were collected and frozen in liquid nitrogen, and stored at −80°C until further use.

### 2.9 RNA extraction and qRT-PCR analysis

To study stress-induced and tissue-specific gene expression, total RNA was extracted using a Plant RNA Reagent Kit (SENO, China) and reverse transcribed into cDNA using a First-Strand cDNA Synthesis Kit (SENO, China). Primers for quantitative fluorescence expression were designed using Primer Premier 6.0 (Supplementary Table S1), with elongation factor 1-alpha (EF-1α) (Tang et al., 2017) used as a reference primer. The real-time PCR system (20 μL) included 1 μL of upstream and downstream primers, 7 μL of ddH_2_O, 10 μL of SYBR Premix, and 1 μL of cDNA. The reaction procedure was as follows: 30 s at 95°C, 40 cycles of 5 s at 95°C and 30 s at 60°C, and 65°C–95°C melting curve detection. Gene expression was analyzed using the 2^−ΔΔ^Ct method (Livak and Schmittgen, 2001). Three replicates were performed for each sample. The results were displayed by means ± standard deviation (SD).

## 3 Results

### 3.1 Identification, Classification and Structure of *StNHX* genes

**FIGURE 1:**
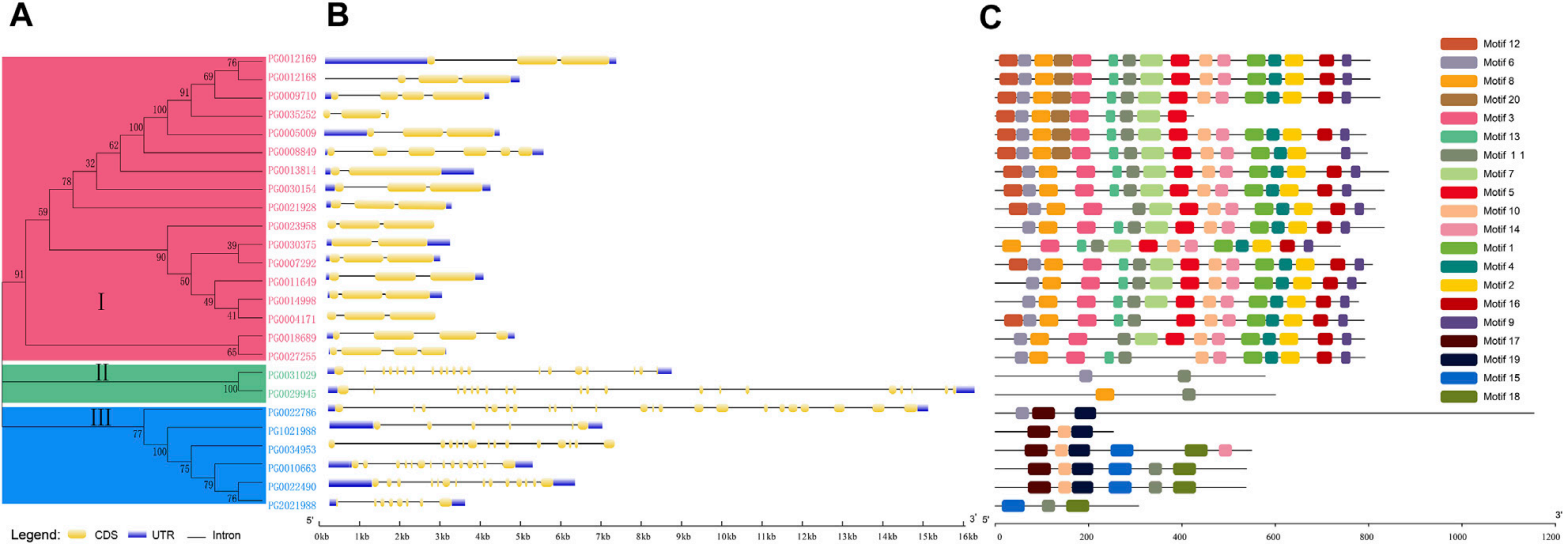
Phylogenetic relationships, gene structures, and conserved motifs of *StNHX* genes. **(A)**: Multiple alignments of 25 full-length amino acid sequences of *StNHX* genes. The phylogenetic tree was constructed using the neighbor-joining method, with 1000 bootstrap replicates. Pink: Class I. Green: Class II. Blue: Class III. **(B)**: Exon/intron structure of *StNHX* genes. Exons are represented by yellow boxes. Introns are represented by black lines. The UTR is marked in blue. **(C)**: *StNHX* protein motifs. Each motif is represented by a different colored box.

A total of 29 candidate *NHX* genes were identified by Hidden Markov Model. Using SMART (http://smart.embl-heidelberg.de), a total of 25 *NHX* family members were found to contain the complete *NHX* conserved domain. The 25 *StNHX* genes were divided into three subgroups based on phylogenetic analysis, with 17 genes clustered in subgroup I, two genes clustered in subgroup II, and six genes clustered in subgroup III (Figure 1).

Gene structure was analyzed by GSDS (Figure 1B). Significant differences were observed in the number of introns of *StNHX* gene family members, with a large gap between the introns in Class I, Class II, and Class III. Class I contained between one and five introns, Class II contained 19 introns, and Class III contained more than five introns. Furthermore, the exon-intron structures were relatively conserved within the same class, indicating that intron loss and gain occurred in the *NHX* gene family during evolution. This may explain the functional diversity of closely related NHX genes.

MEME was used to analyze the amino acid sequences and predict the full-length protein sequences of StNHX members. The 20 identified motifs were named Motif 1-Motif 20 (Figure 1C), and motifs were found to be conserved within each subgroup. Motifs 1, 2, 3, 5, 8, 12, 13, and 20 all contained 41 conserved amino acids; motifs 4, 6, 10, 11, and 14 all contained 29 amino acids; motif 9 contained at least 21 amino acids; motif 16 contained 33 amino acids; motif 19 contained 47 amino acids; and motifs 7, 15, 17, and 18 contained a maximum of 50 amino acids (Figure 1D). Motif 12 dominated the n-terminal of the first subgroup, and motif 9 dominated the C-terminal in class I, which contained between 9 and 16 motifs, the largest number of any subgroup. The second subgroup contained only two motifs, and the third subgroup contained between three and six motifs. The conserved domain contained motifs 12, 6, 8, 20, 3, 13, 11, 7, 5, 17, 19, 20, 15, and 18. Motifs 3, 5, 7, 12, and 13 were found only in Class I. Motifs 10, 15, 17, 18, and 19 were found only in Class III. Other motifs, including 1, 4, 2, 16, and 9, were found only in Class I. The motif diversity among the NHX classes implies that the functions of these proteins may have changed during evolution. Motifs 6, 8, and 11 were found in all classes. Conserved motifs have a high degree of similarity and genes containing the same motif are likely to be functionally similar, indicating that these conserved features may play crucial functional roles. In general, the motif composition of each class was relatively conserved and the gene structure was similar, which demonstrates the reliability of the evolutionary analysis.

A: Multiple alignments of 25 full-length amino acid sequences of *StNHX* genes. The phylogenetic tree was constructed using the neighbor-joining method, with 1,000 bootstrap replicates. Pink: Class I. Green: Class II. Blue: Class III.
B: Exon/intron structure of *StNHX* genes. Exons are represented by yellow boxes. Introns are represented by black lines. The UTR is marked in blue.
C: StNHX protein motifs. Each motif is represented by a different colored box.

### 3.2 Chromosomal location and duplication events of potato *NHX* genes

Pink: Class Ⅰ. Green: Class Ⅱ. Blue: Class Ⅲ. The scale located on the left panel indicates chromosome size in bases. The chromosome number is indicated at the left of each chromosome Blue box: tandem repeating gene pair.

**FIGURE 2:**
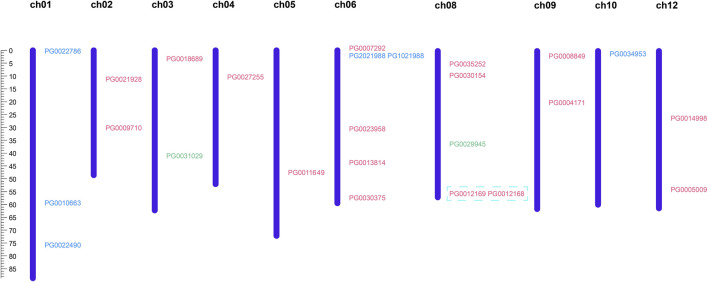
Analysis of chromosomal distribution and duplication events of *StNHX* genes. Pink: Class I. Green: Class II. Blue: Class III. The scale located on the left panel indicates chromosome size in bases. The chromosome number is indicated at the left of each chromosome Blue box: tandem repeating gene pair.

The chromosomal distribution of candidate *StNHX* family genes was predicted using GSDS (Figure 2). A total of 25 *NHX* genes were unevenly distributed across 10 chromosomes, with chromosome 6 containing the most genes (6), followed by chromosomes 4, 5, and 10 (1), and chromosomes 7 and 11 (0). Two Class II genes were located at the top and bottom of chromosomes 3 and 8; three Class III genes were located on chromosome 1; and two Class III genes were located on the distal ends of chromosomes 6 and 10. Members of Class I were mainly concentrated on chromosomes 6 and 8, the rest of the chromosomes contained between one and two genes.

In order to clarify the evolutionary history of the *StNHX* gene family, we used MCScanX to study duplication events. In plant genomic evolution, tandem duplication and fragment duplication help to expand both the number of members and functions of a gene family (Niu et al., 2021). While no large fragment replication events were identified, tandem repetition was detected in potato. One tandem repeating gene pair was identified: *PG0012168*:*PG0012169* (Figure 2).

### 3.3 *StNHX* evolutionary analysis and classification

**FIGURE 3:**
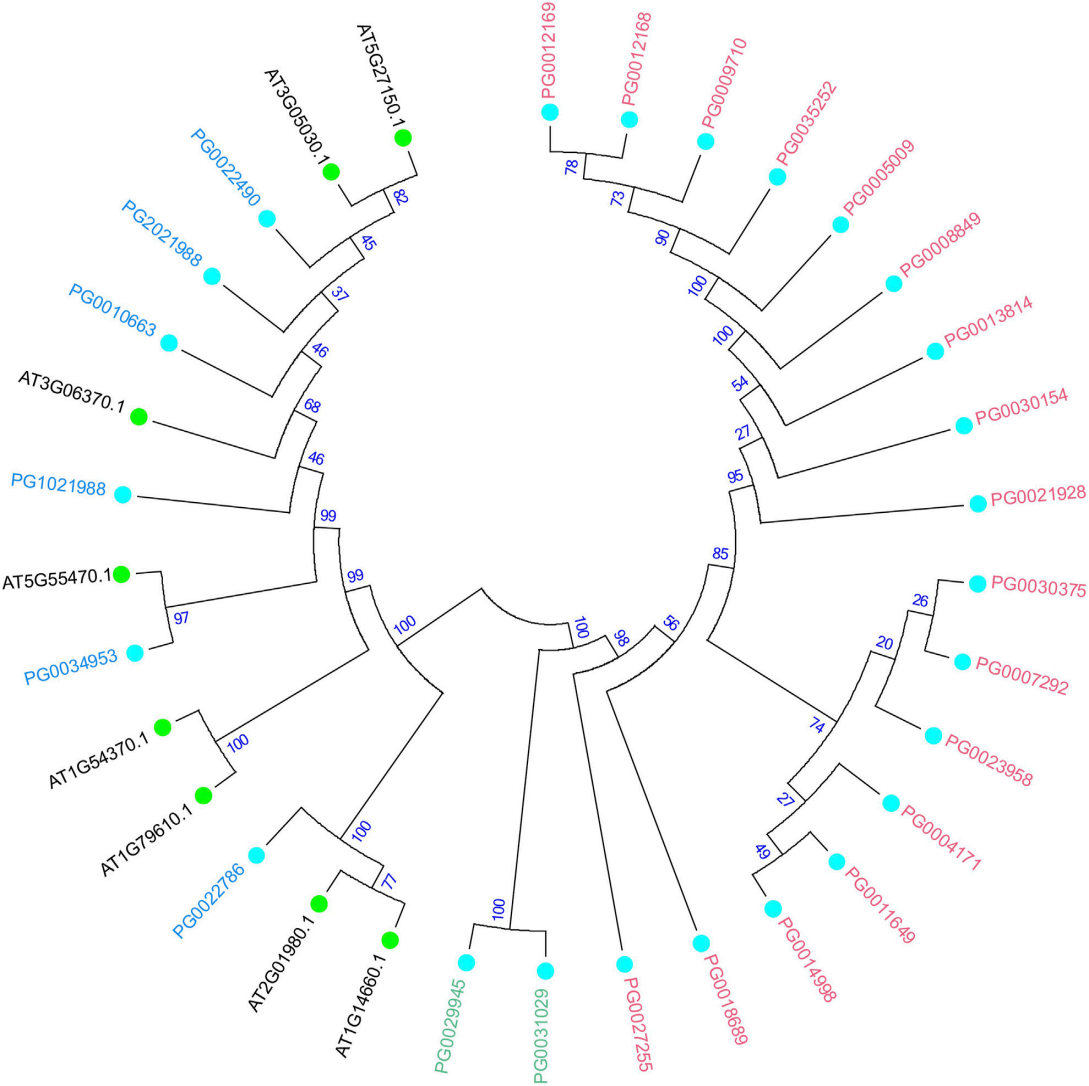
Phylogenetic analysis of NHX proteins in *Arabidopsis thaliana* and potato. The number at the branch of the evolutionary tree indicates the confidence of the branch, with higher values indicating higher reliability. *A*. *thaliana* and potato are shown the different colors, with light blue circles representing *StNHX* genes and green circles representing *AtNHX* genes. Pink: Class Ⅰ. Green: Class Ⅱ. Blue: Class Ⅲ.

In order to further study the potato *NHX* gene phylogenetic lineage and functional characteristics, the NHX protein sequences from *A*. *thaliana* were analyzed using the neighbor-joining (bootstrap = 1,000) method. The *StNHX* gene Class III was closely related to *A*. *thaliana* in one cluster, and Classes I and II were closely related in one cluster. Cluster analysis indicated that the Class III genes of *A*. *thaliana* and potato shared high homology, which suggests that these homologous genes might have similar functions (Figure 3).

### 3.4 Physicochemical properties and subcellular localization of StNHX proteins

The StNHX proteins ranged in length between 252 (*PG1021988*) and 1,153 (*PG0022786*) amino acids. The molecular weights ranged from 27516.32 (*PG1021988*) to 127860.87 (*PG0022786*) kD. The isoelectric point (pI) values ranged from 4.96 (*PG1021988*) to 9.30 (*PG0022786*). The isoelectric points of eight StNHX proteins (*PG1021988*, *PG0018689*, *PG0004171*, *PG0011649*, *PG0021928*, *PG0030154*, *PG0013814*, and *PG0022786*) are in the acid range (<7). The isoelectric points of 17 StNHX proteins were in the basic range (>7). CELLO v.2.5 subcellular localization analyses indicated that all 25 family members were located in the plasma membrane (Supplementary Table S2).

### 3.5 Cis-acting elements of potato *NHX* genes

**FIGURE 4:**
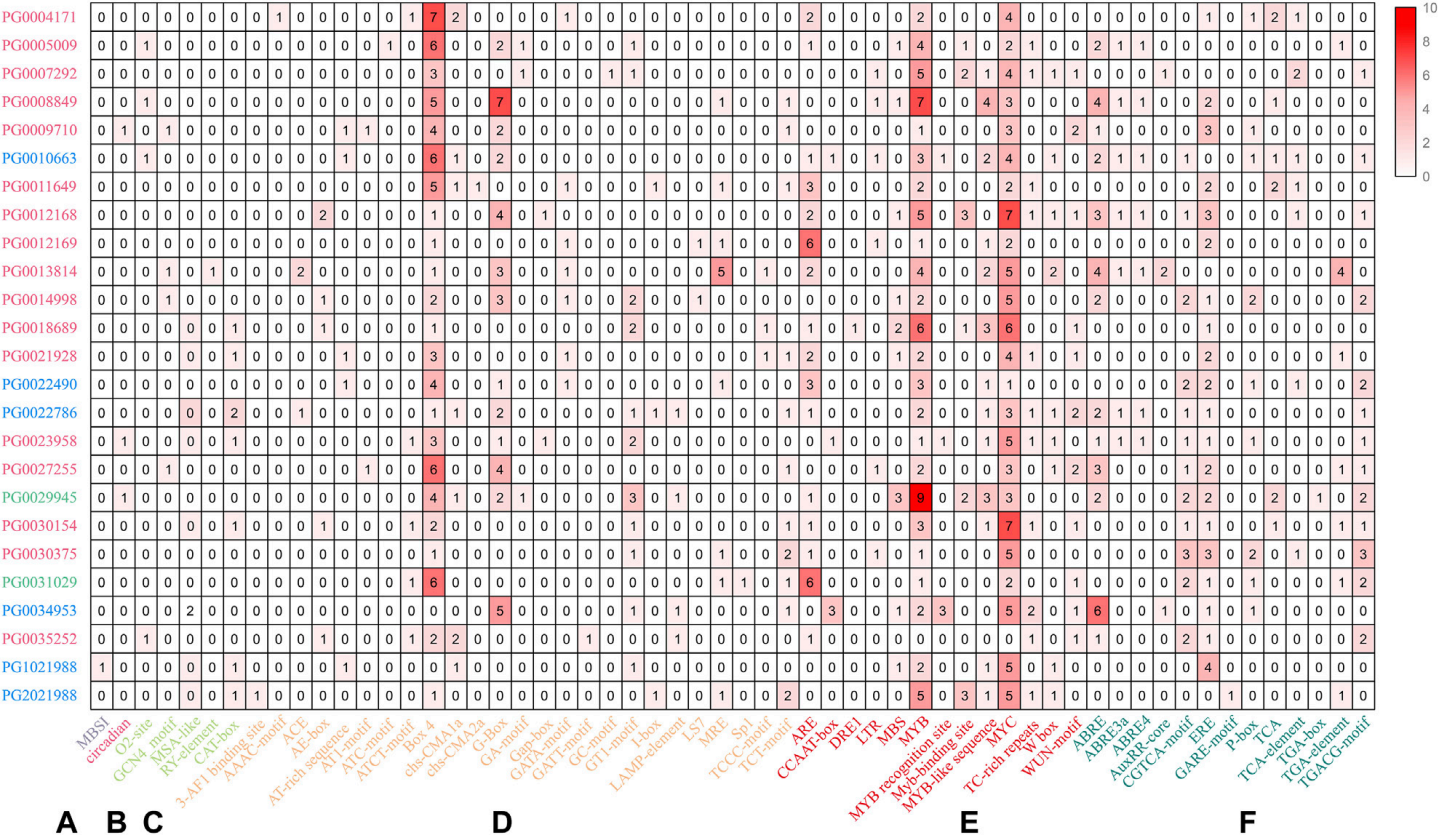
The promoter regions (1,500 bp upstream of the ATG translation start codon) of *NHX* genes were analyzed. Numbers of cis-acting elements are indicated in red, with darker red shading representing higher numbers. Pink: Class I. Green: Class Ⅱ. Blue: Class Ⅲ. **(A)** biosynthesis regulation. **(B)** circadian rhythm regulation. **(C)** growth and development regulation. **(D)** light response regulation. **(E)** plant hormone regulation. **(F)** stress response regulation.

In plants, cis-regulatory elements control the expression of target genes by interacting with transcription factors (Priest et al., 2009). Therefore, identification of potential cis-regulatory elements in the promoter regions of *NHX* genes can provide information regarding their transcriptional regulation. The 1,500 bp upstream promoter sequences of *NHX* genes were selected and analyzed using PLANTCARE. The results showed that besides the two conventional promoter elements (TATA-box and CAAT-box), the *StNHX* family genes possessed 58 cis-acting elements. These cis-acting elements were divided into six categories: biosynthesis, circadian rhythm regulation, light response, plant hormone regulation, stress response regulation, and growth and development (Figure 4). One cis-acting element, MBSI, is a MYB-binding site involved in the regulation of genes for flavonoid biosynthesis. Another cis-acting element, Circadian, is involved in circadian control in plants. Growth and development-related regulatory elements include O2-site, GCN4_motif, Box 4, RY-element, and CAT-box. Notably, all the selected NHX genes contained the MYB and MYC motif except PG0025352, which are cis-acting regulatory elements involved in involved in environmental adaptation. Twenty-five light response elements were identified, and 13 stress and hormone response elements. The promoters of the *NHX* gene family were found to all contain these three related elements, suggesting the functional diversity of this gene family.

**FIGURE 5:**
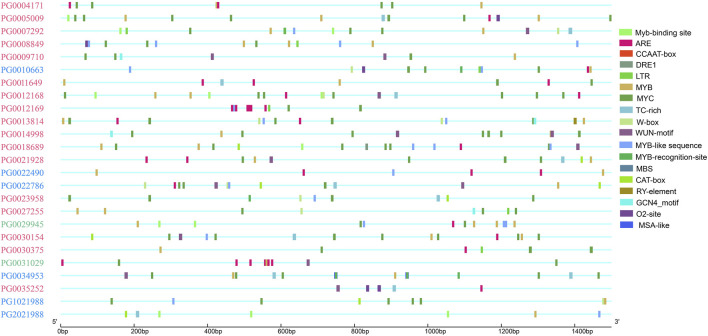
Distribution of cis-elements related to stress response and growth and development in the promoters of *NHX* genes. Pink: Class Ⅰ. Green: Class Ⅱ. Blue: Class Ⅲ.

Both stress response and growth and development elements were selected for further analysis (Figure 5). The results showed that stress-related elements were widely distributed, while growth-related elements only appeared in some *StNHX* family member promoters. These results suggest that the *StNHX* gene family plays an important role in stress response, and a few of these genes may play a regulatory role in growth and development.

### 3.6 Protein-protein interaction networks of potato *NHX* proteins

**FIGURE 6:**
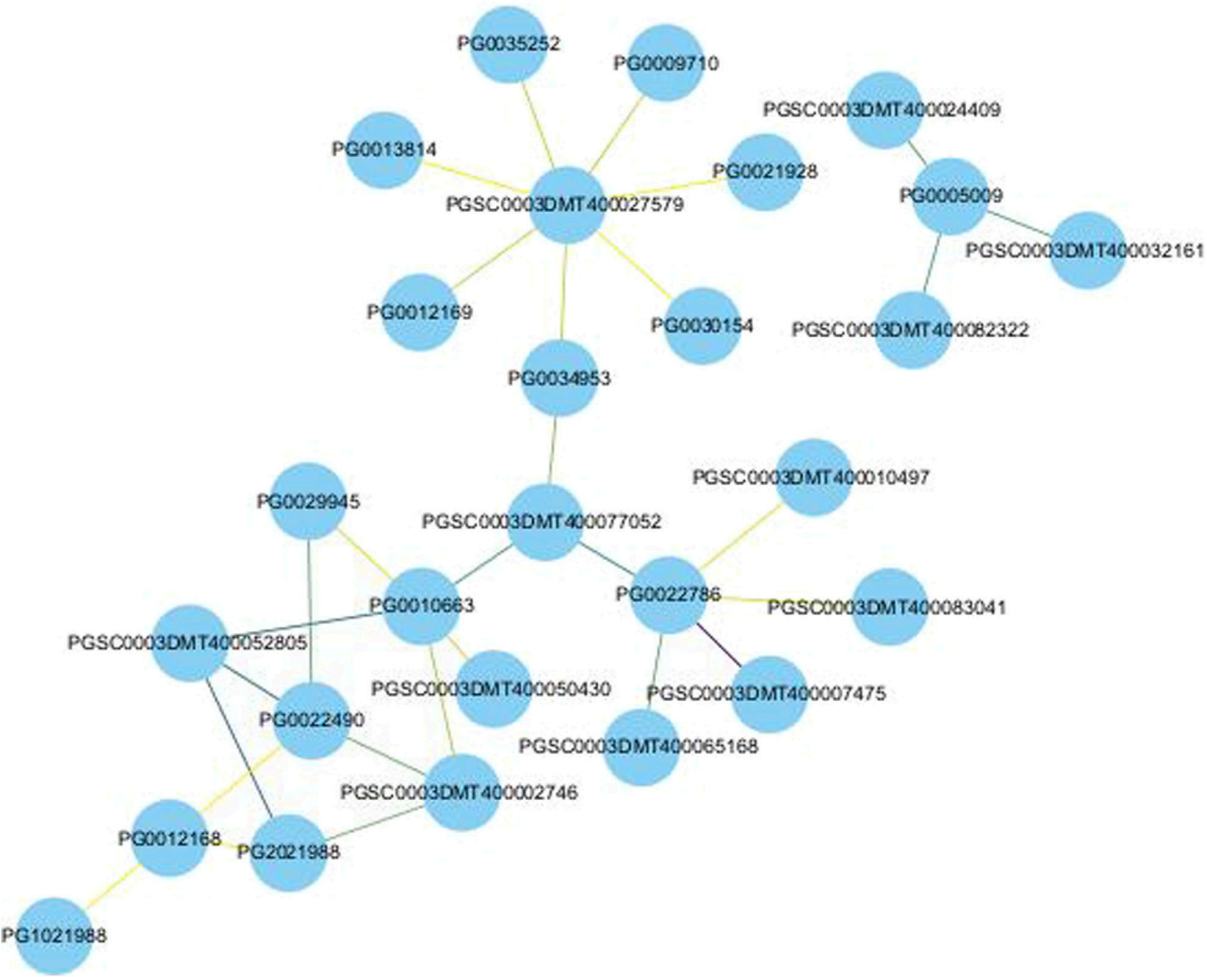
Schematic representation of *StNHX* protein-protein interaction (PPI) network. Different colored nodes indicate different proteins. Darker colors indicate higher core PPI values. The edge color is shown from yellow to purple in accordance with the combined score (>0.700).

In order to identify the function and the mechanism of action of NHX genes, the protein interaction networks (PPIs) of NHX proteins were built using STRING. The results showed that the PPI networks contain 15 StNHXs and can be divided into two groups. For example, PG0005009 was predicted to interact with PGSC0003DMT400024409, PGSC0003DMT400032161, and PGSC0003DMT400082322. PG0013814, PG0021928, PG0022490, PG0030154, PG1021988, PG2021988, PG0029945, PG0012169, PG0035252, PG0034953, PG0010663, PG0022786, PG0012168, and PG0009710 exhibited strong or weak interaction relationships. As shown in Figure 6, some of these proteins interacted strongly, with darker lines indicating higher core PPI values. There are 27 nodes and 29 groups of interaction relationships in the interaction network. PG0022786 was predicted to interact with five proteins, of which three belonged to plasma membrane ATPase (PGSC0003DMT400065168, PGSC0003DMT400010497, and PGSC0003DMT400083041) and one belonged to the protein kinase superfamily (PGSC0003DMT400050430), which play important roles in plant development and stress response. PG0010663 was also predicted to interact with five proteins, of which two belong to the serine/threonine-protein kinase family (PGSC0003DMT400050430 and PGSC0003DMT400052805), indicating that StNHXs may be involved in abiotic stress response. In addition, we found that PGSC0003DMT400027579, which is a sodium/hydrogen exchanger 8-like and salt overly sensitive gene, was predicted to interact with seven StNHXs, indicating that these StNHXs may participate in salt stress response. PG0005009 was predicted to interact with PGSC0003DMT400024409 and PGSC0003DMT400032161, which are heavy metal ATPases and likely participate in copper transport. PGSC0003DMT400082322 belongs to the cytochrome P450 CYP82 gene family, which is involved in growth, metabolism, and stress response regulation. Interestingly, PGSC0003DMT400077052 was located in the center of the whole protein interaction network, and may represent the core of potato StNHX protein family interaction.

### 3.7 Synteny analysis of potato with other crops

**FIGURE 7:**
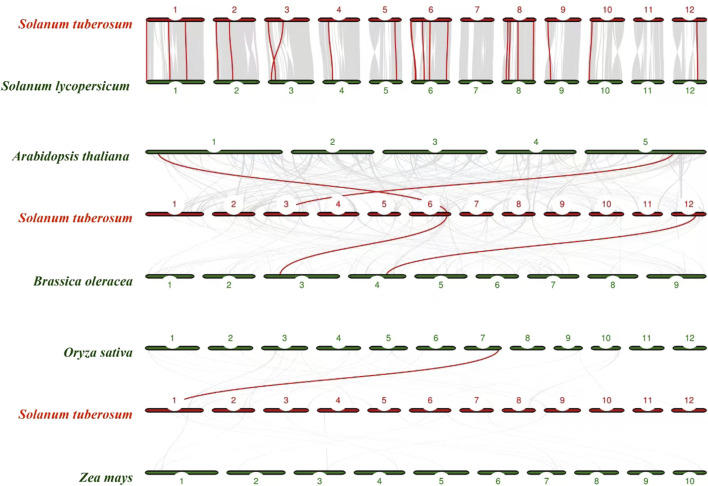
The collinear relationship between potato and other crops. Red lines indicate the collinear gene pairs of potato and other crops.

We further constructed a synteny analysis between *NHX* genes in potato and *NHX* genes in many plants, including *A. thaliana* (L.), *Brassica oleracea*, Oryza sativa、Zea mays (Figure 7). The *StNHX* genes were homologous to genes in other plants, and syntenic conservation was observed among Solanum lycopersicum (22 orthologous gene pairs dispersed on all chromosomes except chromosome 7 and chromosome 11), *B. oleracea* (2 orthologous gene pairs dispersed chromosome 6 and chromosome 12), *A. thaliana* (L.) (2 orthologous gene pairs dispersed chromosome 3 and chromosome 6), Oryza sativa (1 orthologous gene pairs dispersed chromosome 1).

### 3.8 Expression analysis of *StNHX* genes in different tissues

We also studied the tissue-specific (leaves, roots, shoots, tubers, stolons, and flowers) expression patterns of *StNHX* genes during the flowing stage in field-grown potatoes. Overall, the expression levels of *StNHX* genes were different across tissues, and there were also great differences among genes (Figure 9). The expression patterns of *PG0023958* and *PG0007292* were similar in roots and stolons, with *PG0023958* exhibiting the highest expression and *PG0007292* exhibiting the lowest. *PG0034953* exhibited high expression in flowers and stolons, but low expression in other tissues. In flowers, *PG0034953* exhibited the highest expression, followed by *PG0005009* with relative expression levels greater than 1, while other genes were weakly expressed. The expression level of *PG0030154* was relatively high in stems, but was weakly expressed in other tissues. *PG0005009* and *PG0022490* were only highly expressed in flowers and stolons, respectively, but were weakly expressed in other tissues. *PG0009710* exhibited the highest expression in tubers. The expression level of *PG0008849* was higher than other genes, and *PG0022490* exhibited the lowest expression in leaves.

**FIGURE 8:**
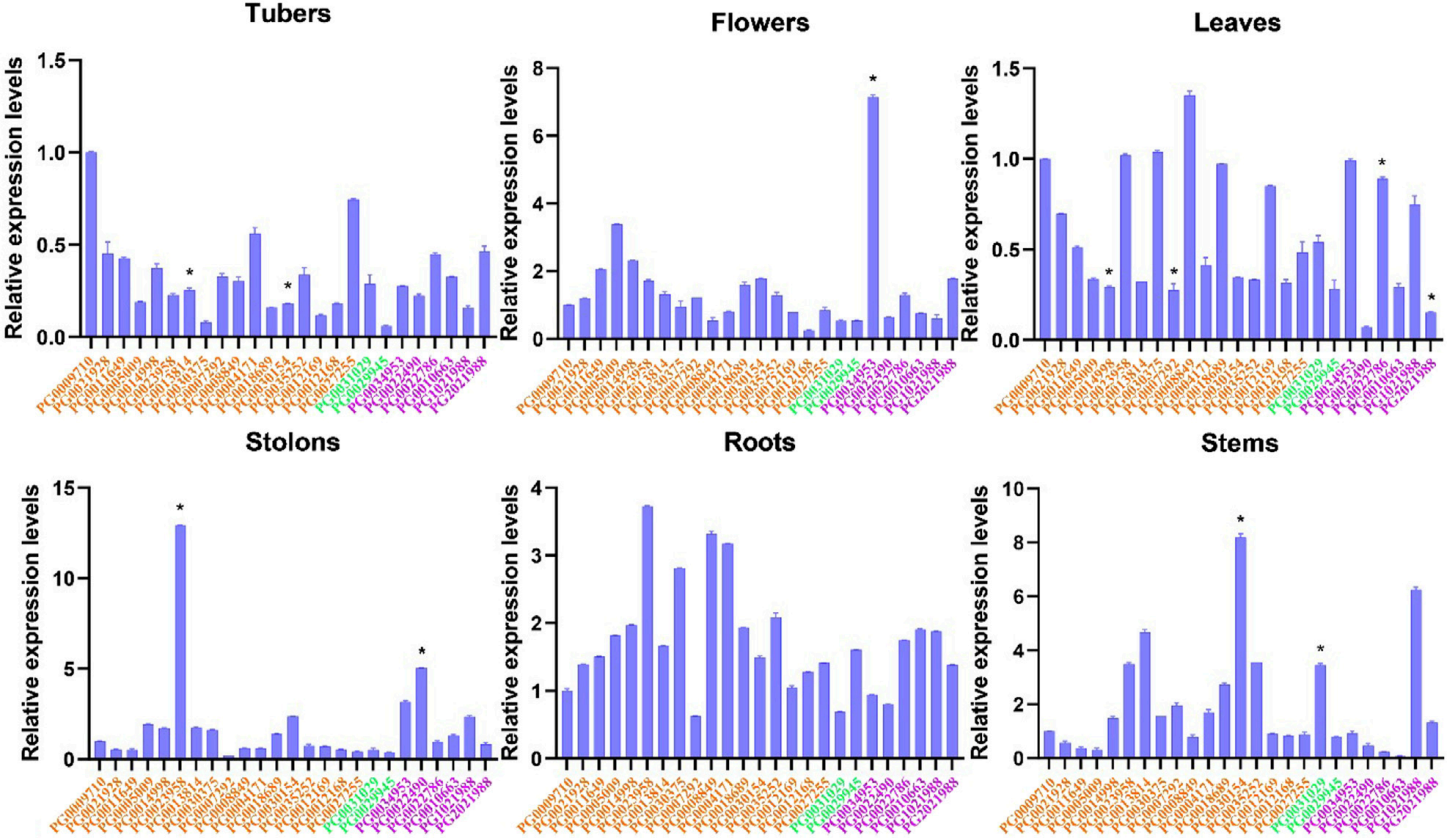
Expression analysis of *StNHX* gene family in various tissues of ‘Jizhangshu NO.8.’ potatoes at the flowering stage. Expression levels of *StNHX* genes in various tissues (leaves, roots, shoots, tubers, stolons, and flowers). The x-axis indicates the different genes. The y-axis indicates the relative expression levels. Relative expression values are presented as means ± standard errors based on three biological replicates with three technical replicates. Significant differential expression is indicated by an asterisk (*, *p* < 0.05).Yellow: Class I. Green: Class II. Purple: Class III.

**FIGURE 9:**
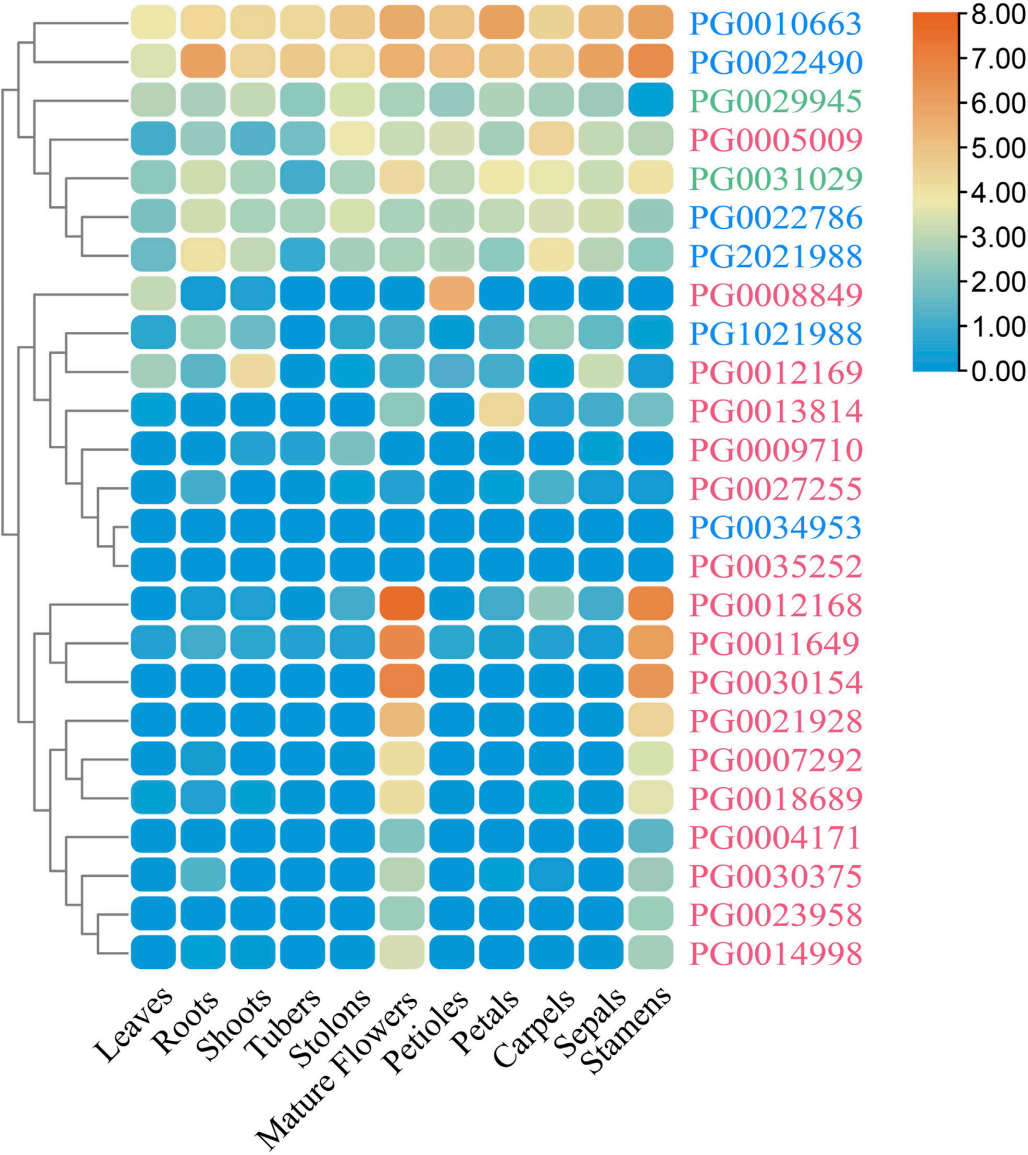
The expression profiles of *StNHX* genes in various tissues. The relative expression levels of 25 *StNHX* genes are taken as the logarithm with base 2 for standardization. Different colored patches indicate the relative expression levels of genes in different tissues (DM potato). Red represents high expression and blue represents low expression. Pink: Class I. Green: Class II. Blue: Class III.

### 3.9 Expression profiles of *StNHX* genes in different tissues

To study the roles of *StNHX* genes in potato, the expression levels of *StNHX* genes in different tissues were analyzed using publicly-available RNA-Seq data. Of the 25 identified *StNHX* genes, 23 were found to be expressed in at least one tissue (FPKM > 1). The 25 identified *StNHX* genes were divided into two major groups based on their expression characteristics. The first group was composed of seven genes (*PG0010663, PG0022490, PG0022786, PG2021988, PG0029945, PG0005009*, and *PG0031029*) with relatively high expression in the majority of tissues. The remaining 18 *StNHX* genes formed the second group, which contained ten genes (*PG0004171, PG0023958, PG0030375, PG0014998, PG0007292, PG0018689, PG0021928, PG0011649, PG0030154,* and *PG0012168*) with high expression only in stamens and flowers, and eight genes (*PG0034953, PG1021988, PG0035252, PG0009710, PG0008849, PG0027255, PG0012169,* and *PG0013814*) with low expression in most tissues. In addition, some of the *StNHX* genes exhibited tissue-specific expression. For instance, *PG0011649, PG0030154*, and *PG0012168* exhibited high expression in stamens and flowers (FPKM > 20). *PG0004171, PG0023958, PG0030375, PG0014998, PG0007292, PG0018689*, and *PG0021928* were only expressed in stamens and flowers (FPKM > 1). Both *PG0012169* and *PG0013814* were highly expressed in shoots and petals (FPKM > 15), respectively, with low or no expression in other tissues (FPKM < 10). *PG0034953* and *PG0035252* were not expressed in any tissues. These results suggest that *StNHX* genes are differentially expressed in various tissues. Furthermore, relatively high expression within the same tissue indicates functional conservation, showing the importance of these genes in potato plant growth and development.

### 3.10 Expression profiles of *StNHX* genes under abiotic stress

**FIGURE 10:**
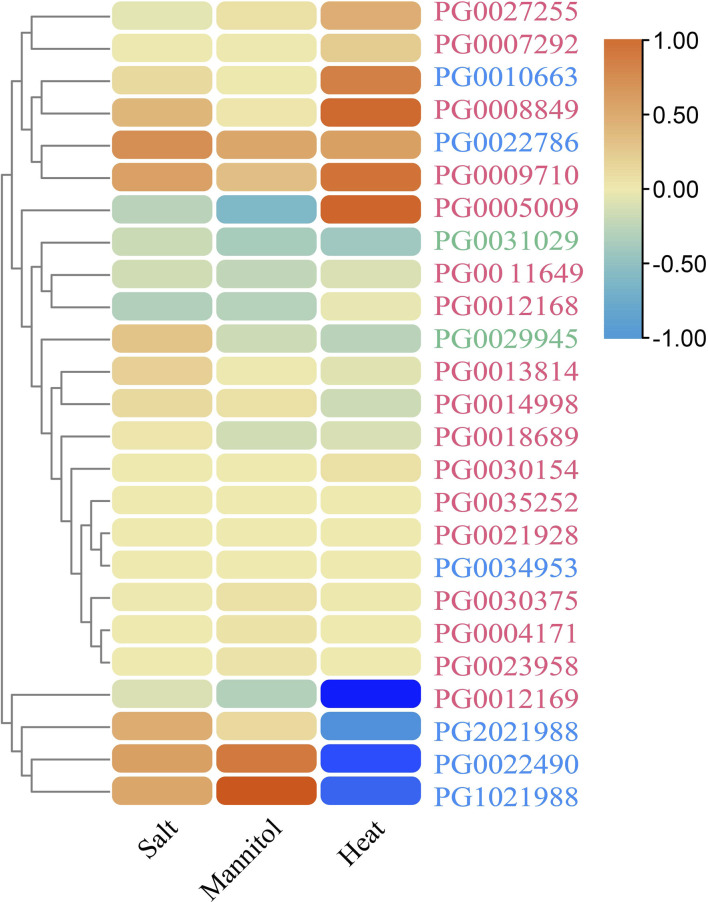
The expression profiles of *StNHX* genes under salt, drought, and heat stress based on transcriptomic data. The relative expression profiles of *StNHX* genes under NaCl, mannitol, and heat stress in DM potato. The log2 mean of each gene FPKM+1 was used to draw a color scale. Pink: Class I. Green: Class II. Blue: Class III.

We next analyzed the transcriptomic data of salt-, drought-, and heat-stressed DM potato (accession no. SRA030516) to identify potential stress-responsive *StNHX* genes. According to the log2(FC) value, it was found that *StNHX* gene expression was induced by salt, drought, and heat, although the expression patterns were different under different stressors. A total of 11, 11, and 8 genes were differentially expressed under NaCl (150 mmol L^−1^), mannitol (260 μM), and heat (35°C) stress, respectively, with |log2(FC)| > 1 and FPKM >1, of which only 6, 4, and 7 genes were not expressed. The 25 identified *StNHX* genes were divided into two major groups (Figure 10). The first group was composed of 21 genes. The second group was composed of *PG0022490, PG1021988, PG0012169*, and *PG2021988*, which exhibited relatively high expression under abiotic stress.

### 3.11 Expression analysis of *StNHX* genes in shoot and root under drought stress

**FIGURE 11:**
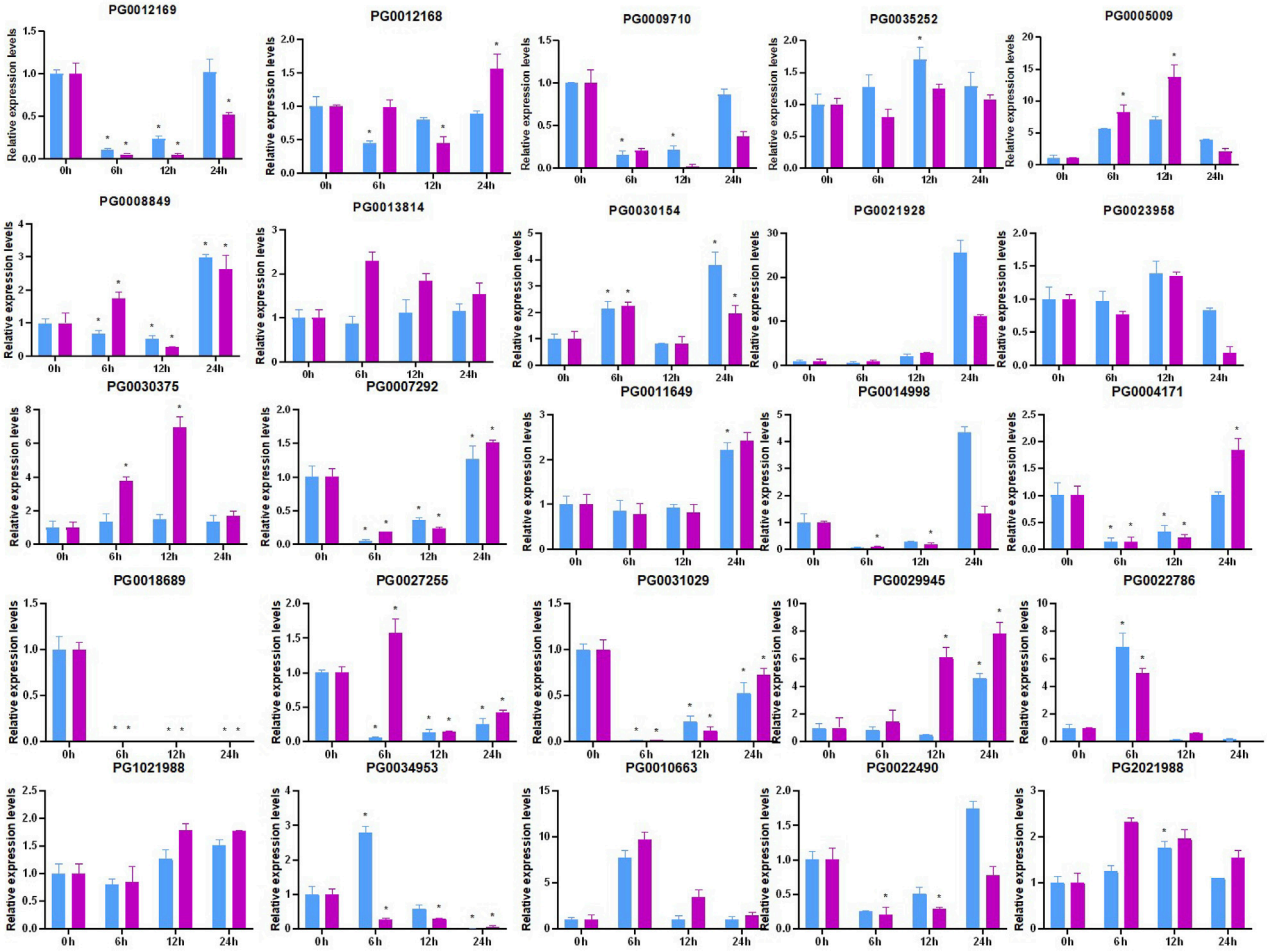
Expression analysis of *StNHX* genes in drought-stressed‘Jizhangshu NO.8.‘potatoes.Blue column: shoots. Purple column: roots. Expression levels of *StNHX* genes under dehydration treatment (15% PEG6000) for 0, 6, 12, and 24 h. The *x*-axis indicates the examined time points after the drought treatment. The *y*-axis indicates the relative expression levels. Relative expression values are presented as means ± standard errors based on three biological replicates with three technical replicates. Significant differential expression is indicated by an asterisk (*, *p* < 0.05).

Environmental stress can cause osmotic stress in plants, which can negatively impact both plant growth and yield. In order to further investigate the expression patterns of these *NHX* family genes in shoots and roots under drought stress, RT-qPCR analysis was performed (Figure 11). Several *StNHX* genes were found to be regulated by drought stress, exhibiting increased differential expression in response to drought treatment. Several *StNHX* genes (*PG0018689*, *PG0031029*, *PG0009710*) were significantly downregulated by short-term drought treatment, compared to control. Others were upregulated and peaked at 24 h, including *PG000417, PG0022490*, *PG0007292*, *PG0014998*, and *PG0012169*, exhibiting a delated response to drought stress. Certain *StNHX* genes (*PG0030375, PG0005009,* and *PG2021988*) were simultaneously upregulated after 6 h of drought stress. Others were induced after 12 h or 24 h, including *PG1021988, PG0011649, PG0029945,* and *PG0021928*, suggesting that some genes were actively responsive to drought stress and might be more sensitive to drought. *PG0022786* and *PG0010663* exhibited similar expression patterns in shoots and roots, with relatively high expression levels only at 6 h. Some genes (*PG000971*, *PG0031029,* and *PG0018689*) were inhibited under drought stress and were downregulated or not expressed. Some *StNHX* genes (*PG0035252* and *PG0027255*) were upregulated in root tissue, while other genes (*PG0008849* and *PG0034953*) were highly upregulated in leaves. The expression of *PG0021928* and *PG0029945* gradually increased from 2 h to 24 h in roots, exhibiting a late response to drought stress. These results demonstrate that *StNHX* genes play crucial roles in drought stress and exhibit different responses in various tissue, and may be involved in the early basal resistance of potato under drought stress.

## 4 Discussion

In this study, 25 potato *NHX* genes were identified, which is a considerably larger number than has been found in other plant species. For example, six members have been in spinach (Li et al., 2021) and sweet orange (Yang et al., 2022), seven in peach (Li et al., 2021) and pepper (Luo et al., 2021), eight in grape (Lu et al., 2019) and *A*. *thaliana* (Gaxiola et al., 1999), 11 in banana (Xu et al., 2021) and 16 in *P. betulaefolia* (Wang et al., 2019). Thus, the potato *NHX* gene family appears to have expanded substantially during evolution.

The physical and chemical properties, gene structure, conserved motif composition, chromosomal localization, and promoter cis-acting elements of potato *NHX* gene members were further analyzed. The isoelectric points of StNHX proteins ranged from 4.96 to 9.30, and most proteins were alkaline, which was consistent with the results of previous studies in pepper (Luo et al., 2021) and banana (Xu et al., 2021). It is speculated that *StNHXs* mainly play a role in alkaline subcellular environments.

The 25 *StNHX* genes were found to have different number of introns, with the number of introns ranging from 1 to 22 and the number of amino acids ranging from 252 to 1,153. The structural differences were relatively large, which may determine the functional diversity of the *NHX* genes (Skolnick and Fetrow, 2000). The cluster analysis indicated that the gene structure of closely related members was similar. By analyzing the phylogenetic relationship between *A*. *thaliana* and potato, it was found that there were two clusters in total. Class III was in the same clade as the members from *A*. *thaliana*, with high homology. It is speculated that the *NHX* genes in potato and *A*. *thaliana* have similar functions. In addition, *StNHX* genes with similar motif composition tended to cluster together, suggesting that *NHX* genes with similar evolutionary patterns have similar protein structure and function.

Gene duplication is a common phenomenon in plants (Wendel, 2000; Kondrashov et al., 2003; Conant and Wolfe, 2008). The evolution of duplicate genes and their subsequent differentiation provide the original genetic resources for adaptive evolution, while also contributing to the development of new genes (Ramsey and Schemske, 1998; Flagel and Wendel, 2009). We found that there were no large fragment duplication events in the *StNHX* gene family, although tandem repetition was detected. A total of one tandem repeating gene pair was identified (*PG0012168*:*PG0012169*), which likely contributed to the expansion of this family.

The *StNHX* promoter regions contained a variety of stress response elements. However, the number and types of elements were different among different genes, indicating that the types and degrees of *StNHX* response to stress are different, and thus the function of the *StNHX* genes is different. The identified cis-acting elements present in the potato *NHX* gene family may play roles in light response, hormone induction, growth and development, and stress response, among other roles. The functions of some of these elements have been confirmed in other species (Yokoi et al., 2002; Adler et al., 2010; Almeida et al., 2017), but the role these elements play in potato remains unknown. Some *StNHX* genes were found to contain multiple hormone response elements, demonstrating that StNHX proteins may act as intermediate transcription factors to respond to hormonal signals and regulate plant growth under stress. Previous analyses of the cis-acting elements of *NHX* gene promoter sequences in chili pepper and sweet orange (Li et al., 2021; Luo et al., 2021) were similar to the results of this study. In this study, PPI network analysis also indicated that NHX proteins exhibited has strong or weak interaction, indicating that *StNHXs* may interact with these proteins and participate in the regulation of potato growth and stress.

Analysis of *StNHX* gene expression in different tissues indicated that the expression of this gene family was different in different tissues. We speculate that *StNHX* genes may play regulatory roles in the development of potato flowers, leaves, and fruits, although the specific functional mechanism needs to be verified in subsequent experiments. Tissue-specific gene expression patterns usually reflect their corresponding biological functions. Previous studies postulated that the *NHX* genes play an important role in plant development and stress response (Wu et al., 2019). Based on the publicly-available RNA-Seq data, StNHX genes exhibited differential expression in the different tissues, suggesting that they may participate in growth and development. In this study, we found that *StNHX* gene expression was tissue-specific. Therefore, we speculate that *StNHX* genes may regulate potato development and play a role in the growth and development of different potato tissues.

The *StNHX* genes play essential roles in the regulation of gene expression to cope with environmental stress (Kang et al., 2022). We found that the *StNHX* genes were differentially expressed under NaCl, mannitol, and heat stress. Several *StNHX* genes appeared to take part in the response to only one stress, suggesting that there are different signaling pathways related to the response to abiotic stress. In addition, some genes exhibited opposite expression profiles under different stresses, implying the presence of a complex abiotic stress signal transduction pathway (Li et al., 2020). Previous studies confirmed that the expression of *NHX* genes could be induced by drought treatments, and that these genes have key functions related to the drought tolerance of various plant species. For example, under 10% PEG stress, the expression of *NHX* genes in grape leaves was significantly increased to five times that of control (Lu et al., 2019). The expression levels of maize *NHX* genes increased in leaves and roots under drought and salt stress. Meanwhile, functional identification showed that maize *NHX* genes play an important role in drought and salt tolerance (Dai et al., 2003). In sweet potato, the expression level of *NHX2* was positively correlated with the drought tolerance of transgenic plants (Wang et al., 2016).

In order to further investigate the expression patterns of these *NHX* family genes in potato shoots and roots under drought treatment, RT-qPCR analysis was performed. We found that several *StNHX* genes are regulated by drought stress. Most of the studied *StNHX* genes responding to PEG treatment, with many of these upregulated, although the degree of induction was different. These results indicate that all *StNHX* genes are involved in the response to drought stress in potato, but different treatment times may have different effects on the expression of these genes. Similarly, shoot- and root-level gene expression was also significantly induced by PEG. However, the regulation pattern was different between the leaves and roots, with some genes upregulated in leaves but downregulated in roots. Such differential expression was attributed to the different ways roots and leaves respond to drought stress. Hence, the specific mechanism should be studied further. Similarly, we observed that the expression of some genes, such as *PG0004171*, was unaffected in the early stage of drought stress, suggesting these genes may not contribute substantially to the physiological responses associated with drought tolerance.

In summary, a total of 25 *NHX* genes were identified in potato in this study. The *NHX* genes were analyzed in depth, including molecular characterization, chromosomal distributions, phylogenetic classification, gene structures, protein domain compositions, conserved motifs, as well as cis-regulatory elements. The *StNHX* genes exhibited diverse exon/intron structures, motif distributions, and phylogenetic relationships, all of which potentially contributed to their diverse functions. Protein-protein interactions network analysis indicated that these *NHX* genes had strong or weak interactions, indicating that the *StNHXs* may interact to be involved in stress responses and potato development. Based on RNA-seq data, *NHX* genes were found to exhibit distinct tissue specific expression patterns. Additionally, the *StNHX* genes responded differently to various abiotic stresses, including drought treatment. The qRT-PCR results further confirmed that the *StNHX* genes are involved in the response to drought stress and showed their importance in potato plant growth and development. These studies and analyses provided an overview information regarding the NHX genes in potato and determined the cadidate NHX genes that regulting drought potato for further investigation.

## Data Availability

The original contributions presented in the study are included in the article/Supplementary Material, further inquiries can be directed to the corresponding authors.
