# Supplementary material for: Genome-wide identification and drought stress-induced expression analysis of the NHX gene family in potato
Source: Front Genet. 2024 Jul 11;15:1396375. doi: 10.3389/fgene.2024.1396375 (PMC11269226; doi:10.3389/fgene.2024.1396375)
Supplement: Supplementary file 1 [file Table1.pdf]

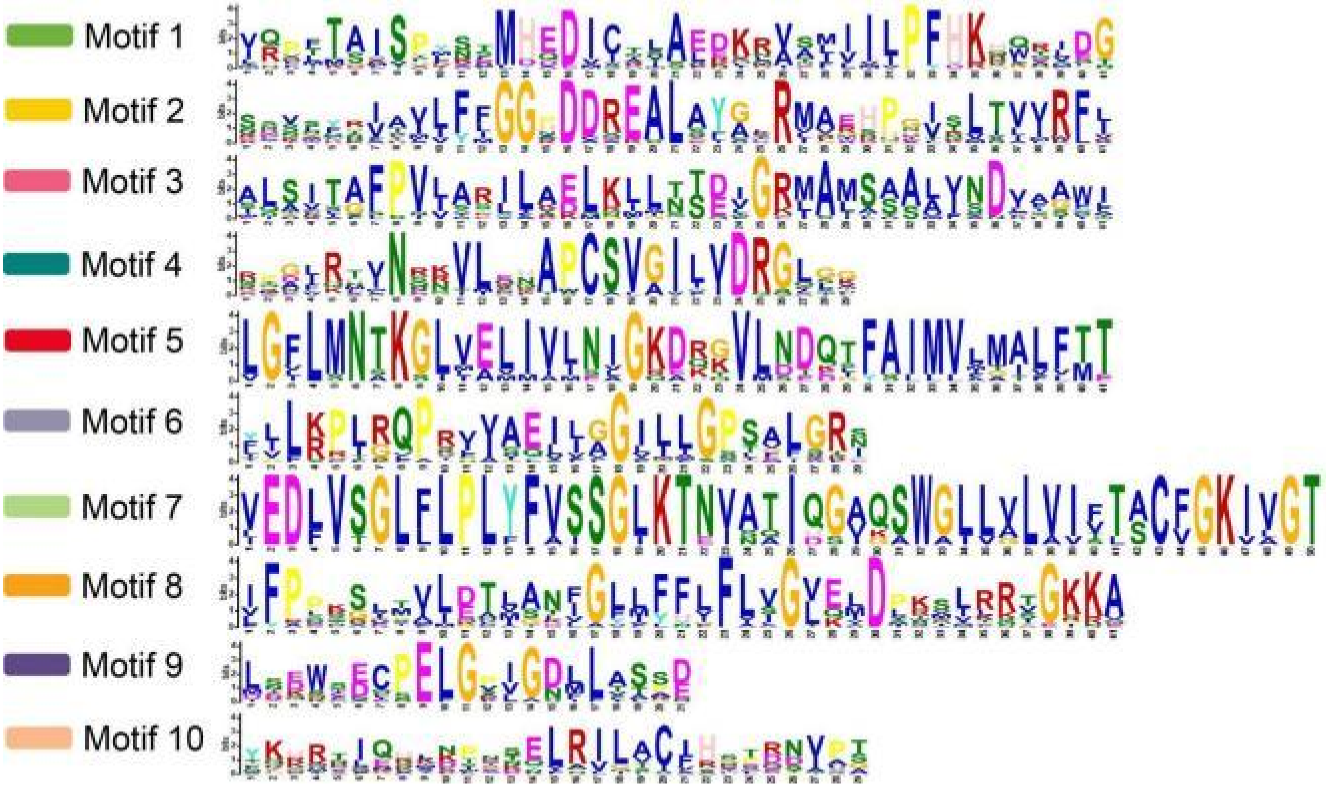
Motif 1
Motif 2
Motif 3
Motif 4
Motif 5
Motif 6
Motif 7
Motif 8
Motif 9
Motif 10

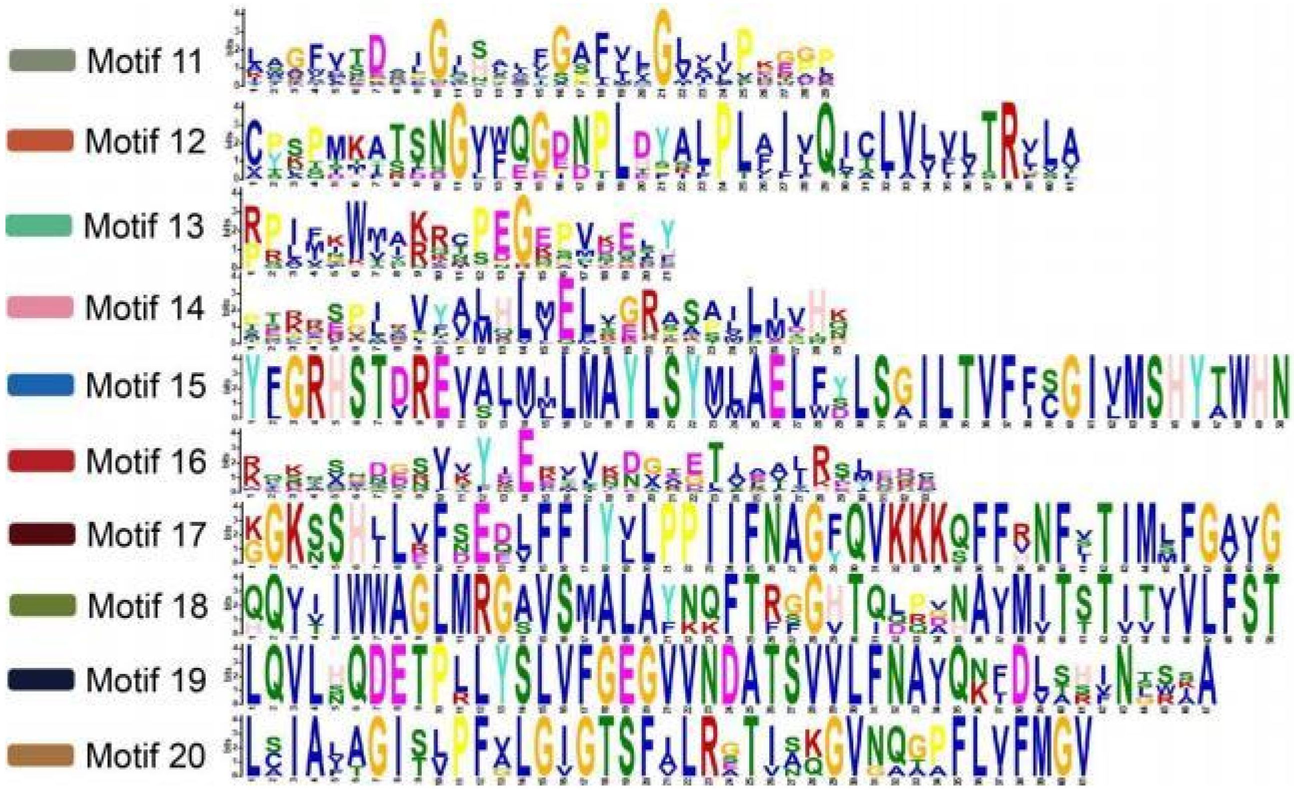

Supplementary Figure 1: Motif logo, the amino acid composition of each motif.

Supplementary Table 1 Primers used in this study.

Gene name	Forward Primer	Reverse Primer	Forward Position	Reverse Position
PG0009710	ATGCACGAAGACATAATTGCT	GAGGACTCTTCGATTCACGTT	1642	1759
PG0021928	CTGCACTTTTATGGATGCTT	AAATACAAATGCACCGAGA	794	924
PG0022490	TTATCTTGCTCGTAAGTGGT	ACCCTGCATTAAATATGATTGGA	194	273
PG0022786	ATACTTTCAAGGCTATGCAAC	GAAAACTTCTCGGACGTCT	3026	3145
PG0010663	TACACCTGGCATAATGTGAC	CTGTCGCTTACAAATCTCC	865	992
PG0011649	ATTTGGTTCTCAAGCGCATC	ATTGTGGGAATAAATTCTCCGTA	197	294
PG0005009	TAGATGTCAAGTCATTGCGTCA	AAGAATAAATGAAGTGCCGAT	326	409
PG0014998	CTTATAATAACAGGGCGACGAA	TTTGAGGCAAGCATATCACC	2167	2257
PG1021988	CCATCATATTTAATGCCGGTT	CCAATAACTCCTTTGCACCA	272	387
PG0023958	ACATTAAAACAAAGACCGGAT	GCCTAATAAAAGTGCTCCGAA	781	895
PG0013814	AAAAGGCAATACCTATAGCTC	GTAACAGAAAGTGCAACACC	395	517
PG0030375	CCTCCTTGGTTAATGACGGTT	GGAATGTGCCTATCCATGCAA	383	482
PG2021988	CATGTTTTGTGTTCCCGTTG	TTGTCATGCTCAGATCGCTTC	383	423
PG0007292	TCTTTTCACTTCTACCGTCT	ATGAATCCATCAATTCGTCCT	198	288
PG0008849	ACCCACAATGGCTATCTCG	TCATTTCCTTCCATTTGCCAA	1650	1752
PG0004171	CTTAATCTCCTTAGCTCCGAA	GCCATACTTTTATGCTCCC	580	686
PG0031029	TTGGTCATTTGTTCCACGTT	GCACCAAGCAGATAATAAGCA	1080	1168
PG0018689	AGGGCAAATATAACTAAGCTG	CAACAATCTTCCCTCCGAT	955	1024
PG0030154	ACGACGAGTTCTTAAACAGG	TAGTTTTATGGCCTTGACAGC	2048	2137
PG0035252	TTGCCCTTTCAGGTACTGGTC	CTCAGGACAACGTTTAGCCAT	620	730
PG0012169	CCCTTTTGGAACACCGAAC	CTAGCGACTATGTCCTCGT	1534	1658
PG0012168	GCTCTATGTATTGCCATTGCT	AACCCCTTGTGATATAGTTGC	376	448
PG0029945	TTCAAGTCCAAGTATCCGGTA	ATCCTGCTAGTAAGTATCCAGT	484	604
PG0027255	GTCAACGCTTATTTAAACGAGA	ATTCGTCGTAAGCTAACACC	1558	1588
PG0034953	TTGACAAAGCCACTCATTCACC	TGTGGCAGATTCATCAAACGA	1384	1483

Ef1a: elongation factor 1- α PG: PGSC0003DMG40

Supplementary Table 2 List of 25 NHX genes identified in potato with protein sequence physiochemical data

Gene	Transcript ID	Chromosome localization	Start position	End position	Strand	Subgroup	Amino acid length	Molecular weight(kD)	Point isoelectric	Subcellular localization
PG0004171	PGSC0003DMT400010686	9	20087848	20090453	+	I	789	87812.88	6.8	PlasmaMembrane
PG0005009	PGSC0003DMT400012865	12	53857871	53862232	+	I	793	86342.04	8.16	PlasmaMembrane
PG0007292	PGSC0003DMT400018809	6	364074	366906	+	I	807	89343.9	8.19	PlasmaMembrane
PG0008849	PGSC0003DMT400022808	9	2015092	2020514	+	I	796	87693.27	8.71	PlasmaMembrane
PG0009710	PGSC0003DMT400025130	2	30230206	30234280	+	I	823	89104.35	8.82	PlasmaMembrane
PG0011649	PGSC0003DMT400030419	5	47574278	47578190	+	I	793	87845.12	6.74	PlasmaMembrane
PG0012168	PGSC0003DMT400031717	8	55473532	55478357	-	I	802	86628.46	8.57	PlasmaMembrane
PG0012169	PGSC0003DMT400031718	8	55458830	55466058	-	I	802	87004.02	8.64	PlasmaMembrane
PG0013814	PGSC0003DMT400035881	6	43663002	43666691	+	I	841	91994.54	6.61	PlasmaMembrane
PG0014998	PGSC0003DMT400038812	12	26207363	26210203	+	I	777	85907.88	8.4	PlasmaMembrane
PG0018689	PGSC0003DMT400048101	3	3262049	3266705	-	I	790	87296.33	5.97	PlasmaMembrane
PG0021928	PGSC0003DMT400056443	2	11322656	11325774	+	I	813	89811.56	5.69	PlasmaMembrane
PG0023958	PGSC0003DMT400061554	6	30590167	30592834	-	I	832	91616.28	7.08	PlasmaMembrane
PG0027255	PGSC0003DMT400070102	4	10403553	10406464	+	I	791	87482.86	7.89	PlasmaMembrane
PG0030154	PGSC0003DMT400077544	8	9537623	9541723	-	I	832	91881.95	5.37	PlasmaMembrane
PG0030375	PGSC0003DMT400078102	6	57441666	57444726	+	I	738	80338.58	7.1	PlasmaMembrane
PG0035252	PGSC0003DMT400085681	8	5000625	5002260	-	I	424	45078.18	9.3	PlasmaMembrane
PG0029945	PGSC0003DMT400076993	8	36226462	36242516	+	II	599	64771.97	7.6	PlasmaMembrane
PG0031029	PGSC0003DMT400079669	3	41054206	41059832	-	II	577	62964.48	7.14	PlasmaMembrane
PG0010663	PGSC0003DMT400027656	1	59438947	59444014	+	III	537	59450.57	8.55	PlasmaMembrane
PG0022490	PGSC0003DMT400057911	1	75833734	75839851	+	III	536	58815.16	7.7	PlasmaMembrane
PG0022786	PGSC0003DMT400058653	1	250845	265748	-	III	1153	127860.87	5.87	PlasmaMembrane
PG0034953	PGSC0003DMT400085382	10	1065667	1072774	+	III	548	61767.3	7.73	PlasmaMembrane
PG1021988	PGSC0003DMT400056555	6	818762	825564	-	III	252	27516.32	4.96	PlasmaMembrane
PG2021988	PGSC0003DMT400056557	6	815313	818044	-	III	306	34272.03	9.11	PlasmaMembrane

Supplementary Table 3：The sequences (gene, proteins, promoter) of StNHXs

Supplementary Table 3 ： The sequences (gene, proteins, promoter) of StNHXs

>PG0009710

ATGGCGGTTAAATGTGCATCATCGCCGATGCATGCCACCTCTAGCGGATTACTACAAGGGGATAATCCGTTGCATTATTCACTCCCTTTGGTGATTGT
ACAAATATGTTTGGTGCTTGTACTCACCCGAGTCCTCGCCTATATTCTCCGCCCACTAAGACAACCGCGTGTTGTTGCTGAGATTATTGGAGGTATTT
TACTAGGTCCATCTGCGCTCGGACGCAACAAAAATTATCTAAACACGATATTTCCACCAAATAGCCTCCCAGTGTTGGACACATTGGCTAACCTTGG
CCTTCTATTCTTTCTTTTTCTCGTTGGGACTGAGTTAGACCCAAGATCTCTTCGTCGAACCGGAAAGAAAGCTCTATGTATTGCCTTTGCTGGAATTA
CTCTCCCTTTTGTATTAGGAATTGGGACATCTTTTGCTCTTCGATCCACAATTGCCCAAGGGGTTAATCAAGCCCCTTTTCTAGTCTTTATGGGAGTTG
CCCTTTCTATCACTGCCTTCCCTGTCTTAGCTCGTATTTTAGCTGAACTCAAGCTATTAACAACTGATGTTGGTCGAATGGCTATGTCCGCCGCGGCG
GTTAATGACGTGGCAGCATGGATTCTACTAGCTCTTGCTATTGCACTTTCAGGTTCGGGGTCTCCAATTGTTTCATTGTGGGTCCTGTTGAGTGGGAC
AGGTTTTATCCTTCTTTGCATAGTGATTGCACCTAGAATATTCAATTGGATGGCAAGGCAATGTCCTGAGGGAGAGCCGGTGAATGAGTTGTATGTT
TGTGCTACATTGGCGATTGTTTTGGCCGCGGGATTTGTCACTGATGCTATTGGAATTCATGCTTTATTTGGGGCTTTTGTGGTTGGTGTTCTTGTACCA
AAAGAAGGGCCATTTGCAGGTGCTTTAGTGGAAAAAGTGGAGGATTTGGTCACGGGGTTATTCCTACCGTTATACTTTGTGTCTAGTGGATTGAAAA
CAAATGTAGCTACTATTCAAGGAGCTCAATCTTGGGGTCTTCTTGTTCTAGTCATAACTACGTCTTGCTTTGGGAAAATTGTTGGTACTATTTGTGTA
TCTCTCATGTGCAAGTTGTCTGTTCAAGAATCGTTAGCACTTGGTTTCTTGATGAATACTAAAGGTCTAGTGGAACTCATTGTCCTTAACATTGGCAA
AGATAAAGGGGTATTGAACGATCAAATATTTGCCATTATGGTGTTGATGGCACTCTTCACAACATTCATGACAACACCGCTAGTTATAGCGACCTAC
AAGCCAGCCAAAATGGCCGTAACAGAGTACAAGCACAGAACAATAATGAGGAAAGACACAACCAAGCAACTTCGAATCTTGACATGTTTCCACGG
CACAAGAAACATTCCCACACTCATAAATCTCATCGAAGCCACTCGTGGAACAGAGAAAAAAGAAGGACTCCGCGTCTACGCGATGCACCTCTTGGA
GCTCACCGAAAGACCCTCAGCAATTCTAATGGTCCACAAGGCCCGAAAAAACGGACTCCCCTTATGGAATAAAGAGAAAGCAGGCGAATCAAACC
AAGTGATTGTCGCATTTGAGACATTCGGACAACTCAGCAAGGTGTCTATAAGACCAACCACAGCAATCTCCCCTATGTCCAGTATGCACGAAGACA
TAATTGCTAGCGCGGAAAGAAAAAGAGTTTCGATGATAATTGTACCATTCCACAAACATCAGAGACTCGATGGACATTTCGAAACAACTCGTGCTG
ATCTCAGAAACGTGAATCGAAGAGTCCTCGAACACGCACCATGTTCAGTTGGAATAATAATTGATCGAGGACTCGGTGGGGCATCTCATGTGTCCG
CTAGCGAAGTTAACTATACGGTCCTGGTTTTATTTTTCGGTGGCCATGATGACCGCGAAGCGCTTGCCTACGGCATGCGCGTCGCAGAGCACCATGG
CATTACATTAAACGTGGTTCGTTTCATAATTGATCCGGCAGTTATTGGAGCGAGTGTCCACGTGGACATCGCTCAGAACTCCGGTCCTGTGCCGGAG
TCATCACAAGAGGACGATATTTATCTTTCTGATCAGAAACAGAAATCAAGCGGTGACAGTTCTATTGTTTTCCAAGAGAGTATTGTAAAGGATGTAA
GAGAAACTATAGAAGTTATTCGCGGATTTAAAAAGTGTAATTTGTTTATAGTTGGGAGGATGTCTGAGGGACAACTGGTTTCAGCATTTGATTCAAA
AAGTCATGATTGTCCAGAATTGGGGCGGTTGGGGAATTTGCTGATTTCCGGTGAAATTTCAACATCGGCATCAGTGTTAGTTGTGCAGCAATATCGA
AGCGAATTACCTCAAGAATCACTTAGGTCTTTGAGGGTTGGAGATTCATCAAGGGTTGGATCATCAAGGATTGGAAATTCAGCAAGAATTCGGCAT
TCAACAAAAGGAGATGATGGTGATGAAGAGGTAACTGAAATTTAA

>PG0022490

ATGGCTTCTGTGCTGGCTTCTCTGTTTCCAAAACTGGGATCTTTGGGTACTTCAGATCATGCTTCTGTTGTATCCATCAACCTATTTGTGGCACTCCTT
TGTGCTTGCATCATCATTGGTCATCTCTTGGAGGAGAACCGCTGGATTAACGAGTCCATTACTGCCCTCATAATTGGTTTGGGTACAGGAGTGGTTA
TCTTGCTCGTAAGTGGTGGAAAGAGCTCACACCTTCTGGTTTTCAGTGAAGATCTCTTTTTCATATATGTACTTCCTCCAATCATATTTAATGCAGGG
TTTCAGGTAAAAAAGAAGCAATTTTTCGTGAACTTCATAACTATAATGATGTTCGGAGCCATTGGTACCCTGGTCTCATGTGCCATTATATCACTAG
GTGCCATTCAAACTTTCAAGAAGTTGGACATTGAATTTCTAGATATTGGGGATTATCTTGCAATTGGAGCAATATTTGCTGCCACAGATTCCGTCTG
CACATTGCAGGTCCTACATCAGGATGAGACACCCCTCCTTTACAGTCTTGTATTTGGAGAAGGAGTTGTAAATGATGCTACATCGGTGGTGCTTTTC
AATGCTATTCAAAACTTCGACCTTACCAGTGTGAATCTCAGTATAGCCCTCAGTTTCCTTGGCAACTTCTTCTATCTGTTCCTTGCTAGCACTTTACTG
GGAGCAGGAACCGGTCTTCTTAGTGCTTACATTATCAAGAAGCTGTATTTTGGCAGGCACTCCACAGATCGTGAGGTTGCCCTTATGATGCTCATGG
CTTACTTATCATACATGCTGGCTGAACTATTCTATTTGAGTGGGATTCTCACTGTATTTTTCTGTGGTATTGTAATGTCTCATTACACTTGGCACAATG

TGACCGAGAGTTCAAGAGTCACTACAAGGCACGCTTTTGCAACTTTGTCATTTCTTGCAGAGACTTTCCTCTTCCTCTATGTCGGCATGGATGCTTTG
GATATCGAGAAGTGGAAATTTGTTGGTGACAGGCCTGGATTATCAATTTCCGTGAGTTCAATACTGATGGGACTAATCTTGCTGGGGAGAGCTGCCT
TTGTTTTTCCATTATCATTCTTCTCCAACTTAATGAAGAAATCCTCGGAGCAAAAAATTACCTTTAGGCAGCAAGTGATAATATGGTGGGCAGGTTT
GATGAGAGGCGCAGTGTCCATGGCACTGGCATATAATAAGTTCACTCGTGGGGGACACACTCAACTGCAGGACAATGCAATAATGATTACCAGCAC
GATAACCATTGTTCTATTCAGCACAATGGTATTCGGTTTAATGACAAAACCCCTTATAAGTCTCCTGCTGCCACCACAGAGGCAATTGAGTACAGTG
TCATCAGGTGCAAATACTCCAAAGTCTCTAACAGCCCCACTCCTAGGCAGTCGAGAGGACTCTGAAGTTGATTTAAATGTTCCAGATCTTCCTCACC
CACCAAGTTTGAGGATGCTACTTACCGCACCAAGTCATAAAGTGCATCGGTACTGGCGCAAGTTTGACGATGCATTCATGCGCCCTATGTTTGGTGG
TCGGGGATTTGCTCCTCCTGCCCCTGGTTCTCCAACGGAACAGGGTCCATGA
>PG0022786
ATGGAGTTGTTAGTAGAAACGAGGCCGTTTAGAGTAGTAGAAGAGTCAATTTCAGCTGAAAGTTCAGGTTCAGATCCTACAAATGCTGTCATTTTTG
TTGGAATCAGTTTGTTATTGGGAATTGGTTGTAGACATTTGTTGAGGGGTACTAGGGTACCTTACTCTGTTGCTTTGCTTGTTCTTGGTATTGGACTT
GGGGCTTTAGAGTATGGAACACATCATGGGTTGGGAAGGATTGGCGATGGTATCCGTATCTGGGCAAACATTGATCCTGATCTTTTGTTGGCGGTTT
TCCTTCCTGCTCTTCTTTTCGAGAGTGCTTTCTCAATGGAAATTCACCAGATAAAGAGGTGTGCAGTACAGATGCTTTTACTTGCTGGGCCTGGCGTG
CTTATTTCCACTTTTTTTCTTGGAGCTGCCCTCAAGATTGCTTTTCCTTATAACTGGAGCTGGTCAACATCATTGTTGCTTGGTGGACTTCTAAGCGCT
ACTGATCCTGTGGCTGTTGTAGCCCTTCTGAAGGAGCTTGGTGCCAGCAAGAAATTGAATACCATAATTGAAGGAGAGTCCTTGATGAATGATGGG
ACGGCAATTGTTGTCTATCAGCTACTTTTAAGGATGGTAACTGGTTGGACCTTTAACTGGGGAGCTGTCATCAAATTCCTAGTACAAGTTTCCCTTGG
GGCAGTCGGCTTTGGTATTGCTTTTGGTATAGCGTCTGTGTTATGGCTGGGATTCATTTTCAATGACACGGTGATTGAGATCAGCCTGACTCTTGCCG
TGAGCTATGTTGCTTACTTCACAGCACAACAAGGGGCTGATGTCTCTGGTGTCTTGACTGTGATGACTTTAGGGATGTTCTACTCAGCTGTAGCTAA
AACTGCTTTCAAGGGAGAGAGTCATCAAAGCTTGCATCACTTCTGGGAAATGGTTTCCTATATTGCAAATACATTAATTTTTATCTTAAGTGGAGTC
GTCATAGCTGAAGGAATTCTTGGTGGTGACAATATTTTCAAAATCTATGATAATTCGTGGGGCTACCTCATCCTTCTTTATGCGCTTATCCTTGTCTC
GCGGGCCGTTGTAGTTGGTGTATTGTATCCATTTTTACGATACTTTGGCTATGGTTTGGACTTGAAAGAAGCTTTTATTCTTGTCTGGGGTGGTCTTC
GAGGTGCTGTAGCATTGTCTCTTTCATTGTCAGTCAAGCGTTCCAGTGATGGCTCTCAATATATCAGTCCTGATACTGGAACTCTGTTCGTTTTCTTG
ACTGGTGGGGTTGTATTTTTGACGCTCATCATCAACGGTTCAACCACACAGTTTGCTTTGCATTATCTGGGAATGGATAAGCTATCAGCAGCAAAGA
AGCGCATATTGAACTACACAAAATATGAGATGTTGAACAAAGCTCTGGAAGCATTTGGTGATCTTGGAGATGATGAGGAACTAGGACCTGCTGACT
GGCCAACTGTTAAAAGATATATTACTAGCCTAAATGATGTGGAGGGTGAACCAGTGCATCCTCATACTTCATCTGAAAATGATGATAATGTGGACC
ATATGCATTTAGAAGATATTCGGATCCGCCTGTTAAATGGTGTTCAAGCAGCATACTGGGAAATGCTCAATGAGGGCAGGATCCCACAAACTATAG
CAAATCTTCTAATGCAATCTGTGGAGGAAGCAATTGATGTAGTATCGCATGAGCCCTTATGTGATTGGAAGGGTTTAAAATCTTATGTTAATATCCC
AAATTATTACAAGTTTCTTCAAACAAGTTTTGTCCACCGGAAGCTTATTACATATTTCACTGTTGAGAGGTTAGAGTCTGCATGCTATATCTGTGCTG
GATTTCTCCGGGCGCATAGAACTGCACGACAGCAACTAAATGAATTTATCGGTGAGAGTGAAATTGCTTCCCTTGTTATCAAGGAAAGCGAGGAAG
AAGGAGAGGATGCACGGAAGTTTCTGGAAGAAGTCGTGTCTCATTTCCTCAGGTTCTCCGAGTTGTGAAAACCAGACAAGTTACTTACGCAGTGTT
GAATCACTTGATTGATTATGTCCATAATCTTGAGAAGATTGGTATATTGGAGGAAAAAGAGATGACACATCTTCACGATGCTGTTCAGACTGATTTG
AAACGGCTTGTAAGAAATCCACCACTGGTTAAATTTCCGAAAATTCGTGATTTGATTAGTGTCAATCCTTTGTTGGGAGCTCTACCTCCTACGGTGC
GTGAGACACTAATTGGTTCAACCAAGGAGATAATGAAGTTACGCGGTGCGACCCTCTATGAAGAGGGATCAAAGGCAACTAGAGTTTGGCTTATTT
CAAATGGTGTAGTGAAGTGGTCAAGTAAGAGCGCGAGTAATATGCATCTACTGCATCCAACTTTTTCACATGGGAGTACACTAGGTTTATATGAAG
TGCTTGTGGGAAAGCCTTACATCTGTGACATTATAACAGATTCCGTGGCTCTCTGCTTTTCTGTTGATAGTGAGAGGATACTTACTGCTCTCAGATCT
GATCCAGCTGTAGAAGATTTCTTTTGGCAGGAAAGTGCACTTGTGCTTGCTAAAGTCTTGCTTCCTCAAATGTTTGAAACGACAACAATGCAAGATA
TGAGAACTCTGGTTGCAGAAAGGTCAACTATGAGTGTATACATAAGAGGGGAAAGTTTTGAATTGCCTCACCATTCCATTGGTTTTCTGTTGGAGGG
ATTTGTTAAATCTCATGGCTCAAATGAAGGGCTTCTTTCAGCTCCTGCACCGTTACTGCCTTTGGCATTGGAACAACAAAGCTTCCACAACACAGAA
GCATCAGTTGTCCATGCAGCTAGTTTTTCTCATCAACCTTCTCAGTATCAAGTTGAGACAAGGGCAAGAGTGATTATGTTTGATATTGCGGGATTTCT

ATCAGGCAGGGGTCTCCAGAGAAGATCGTCTTCGCTATTATCGCACTCAATTGATCATCCCTCAAGGTCTTTTAGTAGAGAGCTTGGTGGTTTAATG
AGCTGGCCAGAAAATACTTTCAAGGCTATGCAACATAGACAGGATGTGGAACAAACTGGTCAACAGGAAATGAATATGTCTACCCGCGCAATGCA
GCTAAACATCTTTGGAAGCATGATAAGCAACACAAGAAGACGTCCGAGAAGTTTTCCTGGAATTAGTGCAGCCAAAACCTCTCACAGTCAGTCATA
TCCAGAAGTTCGATCAGATCGCGCACAAACACTTGTTTCTGTTCGATCTGAAGGATCTACCACATTGCGAAAGAATGCTCAAGTGCAAGGAGAGAA
TAAAGATATGAGTATACAACTTCCAAGCGCACCAATTGAACAAAGTGACACAAGAGAGTATCGAGTGATGATTCTGGTGGCGAGGATGAGCATCTC
ATCAGAATTGATTCGGGAAGACCAAGCTTTCCTCAATGGATCACAGAGGAAGATAAAACATCATAG
>PG0010663
ATGGTGTTTGACTTTGGGACGCTGGTGGCAAGTTTGAACAGATTGTCAACTTCTGATCATCAATCTGTTGTGTCGATTAACTTGTTTGTTGCGCTTAT
CTGCGCGTGTATTATTATCGGTCATCTGCTCGAGGAAAATAGATGGATGAATGAGTCCATTACTGCCCTTGTTATCGGTCTTTGCACTGGAGTTGTCA
TTCTACTAATAAGTGGAGGAAAGAACTCACATATTTTAGTCTTCAGCGAAGATCTTTTCTTCATTTACCTTCTTCCACCTATCATTTTTAATGCTGGGT
TCCAGGTGAAAAAGAAATCATTTTTCCGCAATTTCAGCACTATCATGCTTTTTGGGGCAGTTGGCACCTTGATATCATTCATTATCATATCATTCGGT
GCTACTAGCATTTTCAAGAAATGGAATATTGGAAACCTTGAAATTGGAGATTACCTTGCTATTGGAGCAATCTTCTCTGCAACAGATTCTGTTTGCA
CCTTACAAGTGCTTAGTCAGGATGAAACACCCTTACTGTACAGTCTAGTTTTTGGGGAAGGTGTTGTGAATGATGCCACATCTGTAGTTCTGTTCAA
TGCTGTCCAGAACTTTGACTTATCTCATATCAACACAAGCAAAGCTCTGGAATTAGTTGGAAACTTTCTGTACTTGTTTGCGTCGAGCACCATCTTAG
GGGTTGCTACTGGTCTACTGAGCGCCTATATAATTAAAAAACTCTACTTTGGAAGGCACTCCACTGACCGTGAGGTTGCGATAGTGATACTTATGGC
TTACCTATCATACATGCTTGCTGAATTATTCTATTTAAGTGCAATCCTCACTGTGTTTTTCTCTGGGATTGTGATGTCTCACTACACCTGGCATAATGT
GACTGAGAGCTCAAGAGTCACCACCAAGCACGCTTTTGCTACATTATCATTTATTGCTGAAATATTCATATTCCTTTATGTTGGTATGGATGCTTTGG
ACATGGAGAAGTGGAGATTTGTAAGCGACAGCCCACAATTATCACTTCAGGTTAGCTCCATACTGTTGGGTCTTGTTTTGGTTGGAAGGGCAGCATT
TGTTTTCCCCCTGTCATTTTTGTCCAACTTGATGAAGAAGTCTCCGGAGGAGAGGATTAGCTTTAACCAGCAAATTATAATATGGTGGGCTGGACTT
ATGCGAGGTGCTGTTTCAATGGCTCTTGCTTATAATCAGTTTACCAGGGGAGGCCATACTCAGTTACGTGCCAATGCCATAATGATCACAAGTACTA
TCACTGTTGTCCTTTTCAGCACAGGGGTTTTCGGGTTGATGACAAAACCTTTAATTAGATTACTACAACCCTCACCAAAACACTTGAGCAGAATGAT
CTCTTCTGAACCGACGACCCCAAAATCCTTCATTGTGCCACTTCTTGAAAGTACACAAGACTCAGAAGCTGATCTGGGCCCAAATGTACCCCGTCCC
CACAGTCTGCGGATGCTCCTATCAACACCATCTCACACTGTGCATCGTTACTGGAGAAAATTTGACAATGCATTCATGCGTCCCGTTTTCGGTGGAC
GAGGTTTTGTACCTTTTGTTCCAGGATCACCAACTGAACCAAGTGGTCATTAA
>PG0011649
ATGGCGACAGCGGCGGCAACATCAGTGAAATTAACCGGGGAATTACCCGGTGCCGTTAATGAAAAATGTTATGCAGAAATACCTCTTCATTCACCT
GGTATATGGGGTTTACCCAATCCGAGTTTATTCCTCGATTTCAATTTGCCGCTGTTGCTTCTCCAATTAGCCGTCATTTTTATCCTCACACAATCCCTT
CATTTGGTTCTCAAGCGCATCCGTCTTCCCCGTCTTATCTCTGAGATTTTGGCAGGTATCATACTAGGACCAACAATGCTAGGAAAGATCCCGAACTT
TACGGAGAATTTATTCCCACAATCAGGAGAAATTTTCATAGATTTAATGTCAAAAATTGGGTATATCTTCTTTATATTCCTAAGTGGAGTGAAGATG
GACCCAAAAGTGGTGTTGAGAAGTGGTTCAAGGGCGTGGACAGTAGGATTACTTGCTGTCATTCTACCAGTTGCCTCTTTTGCGAGCTTGTATTTTG
GATTCTTTTCACAAGATGCAAATATGCACAGATATCGACAACCAGCCACCCAAAGTATTTTCTTGATACAAGGTTTGATTGCATTTCCTGTGGTTGCA
TCTCTCCTTGTTGACCTGAAAATCATGAATTCTGAGCTTGGTCGTCTTGCTCTGGCATCAAGTTTGATCAGTGACTTATTTAGTAATCTGGGGTTGAC
TATCTTCTCCACCCTTAGAATTGGATTATTGGCAGAGATTACGACGGTTATCTCTGTACAGTCTTTTGTTCTACTTCTTGGAATAATCTTGCTTATTGT
GTTTACTGTGCGACCTATTTCACTCTGGGTCATCAAAAGGACTCCAGAAGGCAGGCCTGTAAATTCCGTTTACATTACTTGGGGATCTGTCTGTGTCC
TGTTAGCTGTGATTCTTGTTGATAATGCAGGGCTTAATTACCAATATGGTCCTTTTATCCTAGGTCTTGTCATACCAGACGGTCCACCTCTCGGGTCA
ACATTGGTAGATAAATTAGAAACCTTAGTATCTGGTATGCTTGCACCTCTTCTGATTACTTATTGTGGTATGAAAGTAAATCTTGTTGATTTGTATGA
TCTCGTCTTCCTGAATTGGGTATGGGTTATGGTGTTCTTTTGTCTCACCGTGAAGTACGCATCAGTTTTCCTTCCAGCATTGGCCTGCAAGGTTCCCCC
AAAAGATGCTGCGGCTCTTGCCTTCATCATGACCTCACAAGGTGTTATTCAGATGTCTTTTTACTTGAACAATGTTATTAATCAGACAGTTGATGGAG
AAACCTTTTCCATGCTAACTGCATCTGTATTACTCATAGCTGCACTCTCACATTTCTGCGTTGGAACACTCTATGATCATACAAGGATATATGCCGGT

TATCAGAAAAGAGACATCCAACATGCTTCAAGCAATTCTGAGTTGCGCTTGCTTTCATGTGCTCATCGATTTGATGATGTCGTAGGTGCCCGAAAGA
TCTTAGATGCTTCTTTCCATGTAAAGAAAGTCCTTTATCTGTGTATGCGCTACACTTGGTGGAACTTGCTGGTCGAGCATCCCCAGTTCTAATTGAT
CATCAGCTTGGCCAAAAGAATACTTCTGGCGTTGCTCGATCGCAAAAGATGGTGGAAGTTTTTATAGCATTTGAAACTCAGTTCTTGGGATCTGCTA
GTACTCATTTCTTCACCTCAATGTCACTGCCAAGGTTCATGCATCAGGACATTTGTTCACTTGCATTCGACAAGTTA
GCGTCCATGATCATACTCCCGTTTCACAGGAAATGGAATCAGCAGGGGAAGATTATCTTGGACAGCAGTAACTTGAGAACAATCAACAATAATGTA
TTGGACTTGGCCCCTTGTTCTGTAGGTATCCTAATTGACCGTCAGAAAATAAAACGTCTAGCAAGTCAATCCGGTAATGAGTCATCAATGTATCAAG
TATCCGTGGTTTTCATGGGAGGCAATGATGATCGTGAGGCGTTAGCTTATGCTAAACGTATGTCAAGATCACCTGAACTTCAGCTGACTGTGGTTAG
ATTCGTGTCATGGGACATCGACGTACGTGAAAACCAGTGGGATGCTGTACTTGATGCTGAGATGTTGAAGGAGGTAAGACTTCTTGGTCAGCACCA
GGATAACATTGTATACAGAGAGGAAAGAGTTAAAGATGGAGCTGAAACAGCTTTAATCATACATGCTATGGAAGAAGCCTTTGATCTTATCATGGT
CGGAAGGCGTCATCGCGACGATCTACAACAACTATTAGGGCTTAATGAGTGGAATGATTTGCCAGAACTTGGACCAGTTGGTGACATGCTTGCAGC
TGCAGAGATCAATAGACCAGTTTCAGTCTTGGTGGTGCAGCAACAAATTGTTAAGAACAAATGA
>PG0005009
ATGGATCATCAGTGTCCATCTCCAATGAAGGCCACGTCTAATGGCATTTTTCAAGGTGATAATCCCCTGGATTTTGCACTGCCGTTGGCCATTTTACA
GATATGTTTAGTTCTTGTCGTCACGAGAGGTCTTGCCTTTTTGCTGCGGCCGCTACGACAACCACGTGTAATTGCTGAGGTTATTGGAGGTATATTGC
TGGGACCATCAGCTCTTGGTCGAAACAAAGGCTATTTGAATGCAGTATTCCCACCGAAGAGCATCACCGTCTTAGACACTTTGGCAAACATTGGTCT
CCTGTTCTTTTTATTTCTAGCTGGCTTAGAACTAGATGTCAAGTCATTGCGTCAGAGTGGGAAAAAAGTCCTTGCCATTGCTGTTACAGGAATTACTC
TTCCGTTTGCTTTGGGAATCGGCACTTCATTTATTCTTCGAGGAACTATAAATAAAGGTGTAAATGCTACCGCATTTCTTGTATTCATGGGTGTAGCG
CTTTCCATAACTGCATTTCCTGTTCTGGCACGCATTTTGGCTGAGTTGAAACTCTTAACTACTGATGTTGGGCGAATGGCCATGTCTGCGGCAGCAAT
TAATGATGTGGCAGCTTGGATTTTACTTGCACTTGCCATTGCCTTATCTGGTGATAATCTATCTCCTGTTGTGCCACTTTGGGTTTTCCTCTGTGGTTG
TGGTTTTGTCATTGGCGCCTATCTTATTGTGCCACCAATTTTTAAATGGATTTCGCGACGTTGCCACGAAGGTGAACCTGTGGATGAGCTGTATATAT
GTGCTACTTTAGCTGCTGTACTTGCAGCTGGACTTGTCACGGATGTTATTGGGATTCACGCGATGTTTGGGGCTTTCGTCATTGGAGTTCTACTTCCA
AAAGAGGGGCCTTTTGCAGGTGAACTGGTTGAAAAAGTTGAGGACCTTGTATCTGGTCTTTTCCTCCCATTATACTTTGTCTCAAGTGGTCTAAAAA
CGAACGTTGCTACAATCCAAGGTATACAATCATGGGGTTTGCTAGTTCTGGTCATATTTACAGCTTGTTTCGGGAAGATTGTTGGTACTTTTATAGTT
TCCCTTCTCTGGAAGATTCCCAAAAATGAAGCTCTTGCTCTTGGATTTCTTATGAACAGTAAGGGACTAGTGGAACTGATTGTCCTTAATATTGGTAA
AGATCGAAAGGTATTGAATGATCAGACTTTTGCTATCATGGTTATGATGGCTCTCTTCACGACCTTTATCACCCACACCTCTGGTTCTGGCTGTTTACA
AACCAGCAAAAATGCTAAGCAAAGGCGACTATAAACATAGGAGAATAGAAAGGAAAAATCCGAATACTGAACTGCGAATACTAACCTGCTTCCGT
AGTTCTAGAAATATTCCATCGGTCATTAATCTCCTTGAGGCCTCACGTGGAACCGAGAGGGGAGAAAGGCTTTCTGTATACGCAATGCATCTCATGG
AGTTCTCGGAGAGACCATCAGCTATCTTAATGGTACACAAGGCTAGACATAATGGCTTGCCCTTCTGGAATAAAGGTCAGCGGTCAGCTAACCACG
TTGTTGTGGCGTTCGAGGCTTTCCAACAATTAAGTCAGGTCTCTGTGCGGCCAATGACATCAATCTCATCACTCTCTGACATGCATGAAGATATTTGC
ATTACTGCTGAGAAGAAAAATATAGCAATGATAATTTTACCATATCACAAGAATCTGAGGCTTGACGGCTCATTTGAATCAACTCGACCTGATTTTC
ACTTGGTTAACAGGAGGGTTCTTGAACATGCTTCATGTTCAGTGGGGATATTTGTTGATCGCGGACTTGGTGGTACTGCACAGATATCTGCAAGTAA
TGTTTCCTTCTCTATTATCGTTCTATACTTTGGGGGTCATGATGATCGTGAAGCACTTGCTTATGGGACTCGTATGGCTGAGCATCCAGGAGTCGAGT
TAACAGTCATTCGCTTCCTAGTGGAGTCAGATTCCTCAGAAGAGATAGTAACAATACATACAGAAGGCACCTCTGCTGCAACATTAGTCTCTGCTGA
TGAGGGGTTTCTCGCTGCTTTCAGAACGAGTATATCAGATGACAGTTCCATCAAATATGAAGAGAAGACTGTTAGAAATGTGTCGGAAACAATTAC
TATCCTACGTGATTACAGTCGTTGCAGTTTGTTTCTGGTTGGTCGAAGGCCTGATGGCGTACTACCTCTTGCATTGAGCCAGAGGATTGATTGTCCTG
AACTCGGGCCAGTCGGGAGTTTGTTGACTTCACCGGAATACACCACAACAGCATCAGTCCTGGTGGTGCAACAGTATTATGATAACTTGTATGCAAA
TAGTTCACAACAGAGGGATGTAGCATCAGTTCCAGAGAATTAA

>PG0014998
ATGAGCAATAGCACGGAAATCATCTCTACCCAAAAGGTTCTTCAATGTGTAACATTCCCTCCAAGAGGATTTTCACTTGGATTTTTAAAAAAGGTT
CAACTCCATGGATATATTCTGTCCCAACAATTGAATCTCAAATACTCATTATATATCTTCTCACACAACTTTTTCATTTTCCTCTCAAGCGCATTGGAT
TTCCCAAGATCGCTTCGGAGATCTTTGCGGGACTAATCCTTGGATCTACTTTTCTTGGGCGTTATAAGAGTTACCAAGAAAAGTTGTTTCCTCTTCCA
AGTCAATCAATTCTGGGTGCACTAACAACATTTGGTTTCCTACTTTTCCTTTTTCTAAGTGGTGTCAAAATGGACACTAGCATGACAAGGAAAATAG
GGAAAAGGGCACTAGTAATAGGTTTTCTTAATCACTTAGCTCCATTGATAACGGGTATGATAACCGTATTTGCATTATCAAGTGATTTCTATCAAGA
AGGTGTAACACCATTATCCATTCCAGTTGAAGTCATAAGTATTGCTAAGACGTCTTTTCCTGTCATCTCTTACCTCCTTAAGGATCTTGGACTCCTTA
ATTCTGAACTTGGAAGATTAGCACTTTCTTCAGCACTTATTAGTGACTTAGTTGGTCTCGCTATCCATGCTTTTATCTTTCTAATTGTTATAGGTGCAA
AGAATACAATCCAAAGAGCAATCACTGATGCAATACTTTTAATTGCTTTCATTATTGTCGTTATTTTTGTCTTTAGGCCACTCATGATGTGGATAGTC
AAAAGGACCCCGGAAGGGAGACCTGTTAAAGACCTTTACATTCTCATGATTGTTCTTGCTGTTTTACTTTCTGGTGTCTTCTCTGCTTGGTTTGAACA
ATCAGTTCTTTTTGGCCCTTTAATCTTTGGTTTGGCCGTCCCTGAGGGACCGCCTTTAGGATCTACACTAGTAGATAAACTTGATCCATTTACATCAG
GATTTCTGTTACCTATTTTTGTAACTGTAATGTCCTTAAGAACAAATCTCTCTGCCATAAATCCCTCTGCTTCTTATACATTTGCAAATATTATCCTAT
TGTGTGTCGGCAGTATAACAAAAATACTAGCATGTTTACTTCCCATGCTATATTGCAAAATGCCCTTAAATGATGCTGCAGCTATCAGCCTCATCAT
GTCTACAAGAGGTGTCGTCGACTTGGCTTCCTACAGCTTTCTAAGAGATGATAAAATCATCAATCAGGCTAGTTTTGCTTTTATGGTAATAGCAACA
GCCGTTACATCAATATTTGTGCAAATCATGGTGAAATGGCTTTATGATCCATCTAGGAAATATGCTGGATATCAAAGAAGAAACTTAATGAATTCCA
ACAACAAGTTACCCATACTAGTTTGCATTCACAATCCTGATAACACTGCTGCTATACTTAGATTGCTGGAGAAATCTAATCCCACTAGGGATTTTCCT
ATAGTGTCCAATGTTCTCCACCTTATAGAGCTACGCGGTCGAGCATCTTCTGTTTTCATCTCTCACCAAGTTCAGACAAAGGCTATTACTGATGTAGC
TTATTCAGAAAATGTCATTCTTGCGTTCCAGGGGTTTGAACGCAATAACTATGGTGCTGTGACCATTCAAGCTTTTACAGCTATCTCCCCACGCAATT
TAATGCATGAGGATATATGTACTCTTTCCCTTGATGTCCTTGCATCCATCATTATATTACCCTTTCATCGAAAATGGGCCGTGGATGGATCAGTAGAA
GTTGAAGATCATGGTTTGAGAACTTTGAACTCTAGTGTTCTTGAAAGGGCTCCTTGTTCTGTTGCAATCCTAGTTGATCGAGGCCAGTTGAAACGTTC
TACCTCTGTCCGTGCATCAGAGAATGCATATTGTATTGCTATTTTATTTTTGGGAGGAAATGATGATCAAGAAGCATTAGCATTTGCTAAACGAATG
GCCATTAGTGGGACCATTAGCCTCACGGTGATACGTTTGATATCCAAGCAAGATGTGAGTTGTGATGTGGATGAAGTAATTGATTTGGATATCGTAG
GTGATTGGAAACAAAGCCGAAGTAGTTGGGAAAATGTGAAGTATATTGAACATTACGTGCATGAAACAACAGAGACAGCATTGTTAGTTCGTTCAC
TAGTAGATGATTATGACCTTATAATAACAGGGCGACGAAACAATACACATTCACCTCTAACAGCAGGACTTGAAGAATGGACTGAAATCCCAGAAT
TAGGAGTCATTGGTGATATGCTTGCCTCAAAGGATCTTAAGACAAGGGCTTCTGTCCTGGTCATTCAACAACAACAAACAACCCTATAG
>PG1021988
ATGGGGTTGGATGCTGTGGCGAGATTGGGGGTGAGTTTATTGTCGGATGATGATCAGGTTTCTGTGGATTCCATAACTCTGTTTGTGGCTCTTCTTTG
TGGATGCATTGTGATTGGTCATCTTCTTGAAGAGAGTCGTTGGATTAACGATTCCATCACTGCTCTGGTTATCGGTTTATGTACAGGAGGCATCATTT
TGTTAACTACTAAAGGAAAGAGCTCTCATCTCTTAGAATTTGATGAACAACTCTTCTTCATTTATGTTCTGCCACCCATCATATTTAATGCCGGTTTTC
AGGTGAAAAAGAAGCAGTTCTTCAGGAATTTTGTCACCATTATGTTGTTTGGTGCCGTCGGCACATTGATATCTTTCAGCATCATATCATTTGGTGCA
AAGGAGTTATTGGGAAAGCTTGATATTGGCTTTCTGGAGCTACGAGATTATCTCGCAATTGGAGCAATTTTTTCAGCAACAGACTCTGTTTGCACTTT
GCAGGTGCTTAATCAGGACGAGACGCCTCGACTCTATAGTCTAGTCTTTGGGGAAGGGGTGGTAAATGATGCAACATCTGTGGTGCTCTTTAATGCG
ATCCAGAAGTTGGACCTCTCCCACATCAATTCAAGGGCTGCTTTAGTGTTCACTGGAAATTTTCTTTACCTGTTTCTTGCAAGCACTTTCCTGGGAGT
TTTGGTAAGTAGCACACTTTCTTGCTTTATGTTTTACGCGCATTTGCCAATCCTTTCTACGTTTGTCCTCTAG
>PG0023958
ATGGCGAAATCGGCATATTTGACGGATAATGCATTTACTGAGCGGAATATTGTATGTTATGATCACTCAGACGTTATTTCAAATGGCTATAAAGTTC
GTAATCCTTTAAGATTTCCTGCACCTCTTATGGTATTTCAGCTCTCCATTATCTCATTAACATCGCTCATCATTGGTGTCGGACTTAAGCCTTTGGGTC
AACCTATCCTTGTTGCACAAGTTCTTGGTGGTATATTGTTTGGACCGTCAGCATTGGGACGTTCAGAAGTATTGAGAGAAACAATATTCCCTCCACG

AGGCGTTATGGCATTAGAAACTGCAGCAACATTTGGTGTTTTCTTCAATCTTTTTGCTATAGGAGTTGAGTGTGACTCGAAAAGGATGTTCAGGCCA
GGGAAAAAAGCAGTTATCATAGGCATCTCTGTATTGTTTTCATCACTTATATCGAATATGGGAATGGCAACACTAATGCAAAGTTTCATC
>PG0013814
ATGGCAACAGACGTTGTAACGGTGGGAAATAAAACAGAGGAAAATATAGTATGTTATTCACCATCCATGATTACAACGAATGGAGTATGGCAGGGT
GAAAATCCACTTGATTATGCATTGCCACTCTTCATTTTGCAATTGACATTAGTTGTTATCTTTACTCGTATTCTCGTTTTCATCTTGAAACCATTTCGA
CAACCTCGTGTTATTGCTGAGATTCTTGGTGGTGTAATTTTGGGGCCATCGGTATTAGGAAGAAGTAAAAGGTTTACTGATACAGTGTTTCCTCTACG
AAGTGTAATGGTGCTTGAGACAATGGCAAATATAGGCCTTCTTTACTTTATTTTTCTGGTTGGAGTAGAAATGGACATTGCTGTTATCCGAAGAACA
GGGAAAAAGGCAATACCTATAGCTCTAGCAGGGATGGTAGTGCCATTTCTTATAGGGGTTTCATTTTCCTTCATGTTGCATAAAAGTACACAAGAGA
CAAAACATGGGACTTTCGTGCTCTTCCTTGGTGTTGCACTTTCTGTTACTGCATTCCCTGTTCTTGCACGAATTCTTGCAGAGCTGAAACTTATCAAC
AGTGAAATTGGTAGGATAGCCATGTCGGCTGCTCTTATAAATGATATTTTCGCGTGGATTCTCTTGGCGTTTGCCATTGCCTTTTCTGAGAACAAAAC
TATGGCTTTGACTTCTGTTTGGGTACTTCTTTCTACAGCAGCGTTTGTTGTTTTCTGTGTTATAATTATCAGGCCTTTAATTGGATGGATGATAAAGCG
AACTCCAGAAGGCGAATCCATAAGTGAGTTCTCTATATGTATCATTCTCTCAGGAGTCATGATATGTGGATTTATAACAGATGCTATTGGAATGCAT
TCTATTTTTGGGGCTTTTATGTTTGGTTTGGTTATTCCTAACGGTCCTCTTGGCCTTACACTTAACGAAAAGTTAGAGGACTTTGTTTCGGGGCTTTTG
TTGCCTCTTTTCTTTGTCATTAGTGGTCTCAAGACGGAGATTAACGCCATTGATGGAGTTGGTTCATGGGCAATATTAGCTCTGGCCATAGTCTTAGC
TTGTGTTGGAAAGATTGCTGGAACAGTCCTTATAACACTGTATTACAGAATACCAATCCATGAAGGCATTACTCTTGGTCTCCTCATGAATGCCAAA
GGACTCATTGAGATGATTGTGATCAATGTTGGTAAAGACCAAAAGGTTCTTGATGACAAATCTTTTGCAATTATGGTTGTGGCAACTGTGCTTATGA
CTGCAATTATCATCCCATTCGTGACGCTGATTTACAAGCCAGCAAGAAAGTTCGCGCCATATAAAAGAAGAACAGTTCAAAGTACAAAACCAGATA
GCGAATTCAGGGTACTGGCTTGCATCCATACACCTAGAAATGTTCCAACAATCATCAATCTTCTTGAAGCATCTTGTCCTACTAAGAAATCTCCAAT
ATGTGTATATGTCCTCCACCTTGTCGAGCTCACTGGACGCTCGTCTGCCATGCTAATTGTCCATAATACACGAAAATCTGGCAGGCCAGCTCTTAAC
AGAACTCAAGCTCAATCGGATCATATTATCAATGCATTCGAGAACTTTGAGCAACATGTTGGAAGTGTCTCCGTGCAACCCCTCACTGCCATCTCCC
CTTACTCCACCATGCACGAAGACATTTGCACCGTGGCTGAGGACAAACGAGTGGCGTTTCTAATCATTCCTTTTCACAAGCAGCAAACAGTTGATGG
TGGAATGGAAATCACAAATCCAAATTTTCGAACGATAAACCAAAATGTATTAGCAAATGCACCTTGTTCTGTTGGGATACTTGTCGACAGAGGACT
AAGTGGTTCCACAAGGTTAGCAGCAAATCAAGTTTCTCACCATGTGGCTGTACTATTCTTTGGTGGACCGGATGATCGTGAAGCATTGTCCTACGGA
TTGAGAATGAGAGAACATCCCGGGATCAACCTCACCGTCATGAGATTCCTCCCTGGAGTATCTGCACTTGAAGAGGCAAGATCAGGTTCAAGAAGA
AGTAATATGAATGATCCAAATGTACTAACAATAGTAACAGATGACGACAAAGAAAAACAGCTAGACGAGGACTATGTCAGCGCGTTCAGGTTAAG
AACAGCTAATGATGATTCAGTTGTATACATTGAAAGAGTAGTGAATCACGGAGAAGAGACAGTAGCAGCAATAAGGACTATAGATCAATCACATG
ACCTGTTCATAGTTGGCAGAGGACAAGGCACCATATCGCCGTTGACAGCAGGACTTACTGATTGGAGCGAGTGCCCGGAGTTAGGTGCAATTGGAG
ATCTATTGGCATCATCAGATAATGCAGCAATTGTTTCAGTGTTAGTAGTGCAACAATATGTAGGAATGGGGCATGGGGATCATATTCTCACACCAGA
CAGTCCTGGACAACAACTACATGAACATTTCAACTTCGGCAATACGAATAATCGGACACAAATCAGAGGACAACAACCTCAATTCCATACACAACC
CTGA
>PG0030375
ATGGGGCGATCAGGCTTTGGTCGGATCCGTGCGTATTCATCCTTAATATTTCGACCAGAAGGCAGAATGGTACTACAGACAATCTCAGATGTTGGTT
TCATGTTTCATGTTTTTGTTCTTGGAGTTCAAGTAGATCCAACAATGCTGAGAAGAGCGGGGAGAAACGCGGTTCTAATAGGTGTTTCATCTTTTGTT
ATGCCTTTTGCACTTGGAGGATTAGCCTGTTACGTATTACCTCATTTGACTGTCATAGATGACGCGACAGCGCATTTCCTGCCTTTGTTATCTGTAATT
AACTCTGCTTCATTCTTTCCTGTGATCACTAGCCTTCTTAGTGATCTTAAGATTCTAAATTCGGAAATTGGTCGAATAGCTACATTAGCCTCCTTGGTT
AATGACGGTTGTATATATGCTGCTTCCATACTTTTAACAACAATAGATGCATCATCAAGTTATTCAAAGTGGAATGGAGTAATGGCCATTGCATGGA
TAGGCACATTCCTGATAGTAATCGTGTTTGCTGTAAGGCCATTGGTGAAACATATTGCTAGGACTATACCGGAAAGAGGGGCTATGAAGGAAAGCC
ATTTTCTAATGATAGCTGTGCTAGCTCTCCTCTGTGGATTTGTGTCCCAGTCTATTGGACAGCCTCCTGCTGTTGGTACTTTTATTTTGGGAGTAGTGG
TGCCAGAGGGACCGCCTTTGGGCTCATCGATGGTCTACAAGATCGATTCTTTATGTACTGGATTGCTGCTTCCTGCTAAGTTTGCTATCAGTGGGTTG

ACATTGGACATATTCTCCCTAGGAAGAGGAAAGAGTCTTTTAGGGGTCGAAGCAGTGATCCTATTGGGTTATTTAGGTAAGTTTGCTGGCACTCTTG
TTTCAGCTGTTCACTTTGCTGTTTCCTTCCAAGATGCAGTACCTCTTGCTCTCATCATGTGCTGCAAAGGAATTATCGAGGCATCCTTTTATATCGGCT
TAAAGGATACTGGAGCAATAACAAGTGAAGCATATGCGCTTTTGTTAATCACAATGTTGGTCATAACAGGAATTGTGAGACCTTTAATTTGGTACCT
CTATGATCCATCAAGAAGGTACTTAGGCTACAGAACAAATAGTATACAACATTTGGATCCGACCAGTGAGCTTCGGGTACAAGTGTGTATTCACAA
CGAAGATAATGTCCCAAGCTTAGTCAATCTTCTTGATGTGTCCAATCCATCTAGAAGGAGACCGATTGCAGTGTTTGTTCTTAATCTAATGGAGCTC
AAAGGCAGCGCAGCTGCTTTACTGGTGCCAACTCATAATAGAAAAGGCAAACCGAAACTCAAGTCTCTGCCTAGCAGAACTGAACATATATCTAAT
GCATTCAACATTCTCGCACATAGAAATCACGGTTCTATGGTTGTTCAACACTTCACCTCCATTGTTCCGTATGCCACTATGCACGATGACATATGTAC
AATCGCGGTGGACAAAGGTGTCAATATTGTGATCATCCCATTCCACAAGCAGTGGGCTATTGATGGAACAGTAGGAGCCAATTTCCCTGCAATTAG
GATGGTGAACCAACAGGTACTCCATAAAGCACCTTGCTCAGTCGGGATCCTAGTTGATCGGGGACAATTAGCCGATAACACACAAATCTTGTTTGG
CCATTCTCTTTTCCGCATCACAATGCTTTACCTAGGTGGTCCTGATGATGATGAGGCACTTGCCTATTGCTGCAGGATGCTAGGGCATCCCCACATCA
CCATGAGTCTCGTCTGGCTCAAACATTCGAGTGACAATATCGAGAAGAGTATGGAGTCACATATGATACAATGGTTCAAGGCTAATAATGTGGATG
CAGGGAGAGTGAGTTACAAAGAAGAAGTGGTGAATGATGCAGTGGGGACAACTCAGGTTCTTCGTTCACTCGAGGACAGTTGTGATCTTTGTATAG
TTGGTAGAGACCACGAACAGTCCGAGTTAACACTAGGGATCAATGAATGGATCGAGTGTCCAGAACTAGGATTTATTGGAGACATGTTAGCTACTT
CGGATTATAGTTTTTCATTGCTCGTTGTGCAACAAACACCACCCGGGACCGAGTTTATAAACATCCAGCCACTGCAGCCTGTTGCTAGCAGTTTTTAT
TCTGGTTCAGGTAAATATAGTCAGCATTCTGGTTCAGGTAAATATAGTCAGCATTCTGGTTATGGCCCTTTTGGGTAG
>PG2021988
ATGCAGATTGGATTGCTAAGTGCGTACCTAATAAAAAAGATATATCTTGGCAGGCACTCAACTGATCGTGAAGTTGCTCTCATGATTCTCATGGCTT
ACCTTTCATATGTGATGGCAGAACTGTTTGACTTAAGCGGAATTCTTACAGTATTTATCTGTGGTATTGTCATGTCACACTATACCTGGCATAATGTG
ACAGTTAATTCTAAAGTGACAACAAGACATGCTTTTGCAACTCTGTCATTTATTGCGGAGATTTTCATTTTCCTTTATGTTGGTATGGATGCCTTAGA
TATTGAGAAATGGAGATTCGTTAAAGACAGTCCTGGAAAATCTGTTGGGGTAAGCGCTGCGCTGCTTGGCCTGGTTCTGGTTGGAAGGGCATGTTTT
GTGTTCCCGTTGTCTCTGTTATCTAATTTCTTGAAGCGATCTGAGCATGACAAGTTTGGTCTCAAACAACAGGTTACAATATGGTGGGCTGGTTTAAT
GCGAGGATCTGTTTCCATGGCATTGGCTTATAACCAGTTTACGAGGTTTGGCCACACCCAACAGCCAGGAAATGCAGTTATGATCACCAGCACAATT
ACAATAGTCCTCTTCAGTACAGTGGTGTTTGGGTTGATAACCAAGCCTCTTGTAAGGTTCTTGTTGCCTTCATCACAAGGTTTCAACAACCTGATCTC
TTCCGAACAATCGTTTGCACGCCCACTTCTCACTAACGGGCAAGAACTCGAAGTTGAGATGGGAAATGTTGATCCCTTTCGACCATCCAGTTTGAGC
ATTCTACTGAAGGAACCTTCTCACACTATTCATAACCATTGGAGAAGGTTCGATGATGCTTTTATGCGACCCCTATTTGGAGGCAGGGGATTCGTGC
CCGATGCACCTGAGTTGTCAAAGGGAGGATGTGATCAATATTGA
>PG0007292
ATGGGTTCACACATGATGGAACCAGATGACTTAGCAACGTATGCTAAAATATTTAGACATGGACAAAATGATTCATCAATTTGCATGTCAATTGGCA
AAATTCAATCCAAAGGTTCCATCTTATTCCATAACACGAACCCATTAGATTACTCTGTTCCTCTCCTACTCTCTCAGCTCTCCTTGGCTTCCCTCTTCA
TTCTTTTCACTTCTACCGTCTTCAAACCCCTCGGTCAACCATCTAATGTCATTCAAATTTTTGGTGGCTTACTACTAGGTCCGTCCTTTCTAGGACGAA
TTGATGGATTCATACAGCTTTTCTATCCTTACAGAAGTCTGGTAGTTATCGATGCTGTTGCATTATTTGGTTATATGTTCTTCTTCTTCTTGATTGGAG
TGCAAATAGATCCTTGGATTCTCAAGAGAGTTGAGAAGAAAGAGTTCATCATTGGTGTATCAACTGTAGCAACTGCTCTTGTGCTAAGCATTAGTAC
CTCTTTTATTCTCATCACTTTTCATATACATATTGATCCATTAGTAGCTGAATCACTCCCTGTAGTAGCTACCATGTCTTCTGTACTCGGATTCCCAGT
CATCGCTCACTATCTCACCGAGCTTAGGATGGTCAATTCTGATTTTGGAAGGATGGCACTCTCTTGTTCCCTTGTTAGTAATATGTTTGGGTTTTTAAT
CATAGCTATTACCAGCCTCTCAAGTCAACCA
TCCGTCGAGAAATTCATGTTTCTACAAAGTATTACTTCTGGTATAGGCTTTACCATGTTTGTATTTCTTGTTGTGAGACCTCTAGTCATATGGAGCAC
GCGAAGAAATCCACCAGGAGAGCCATTGAAACAAAGTTTCATTTGTATGGTATTTATTGGGGTCTTGCTTTCTGGATTTTGCAGCAAGGCTTTAGGA
CTAAACCTCTTCTACGGGCCACTAGTTTACGGTCTAGCCATACCTGCTGGACCACCACTAGGCTCTGCCCTCGTCGAGAAGCTTCAATTCATTGTTTC
ATGGCTCTTTATGCCTATCTACTTTGTCAAAACAGGTTTGGTTACTGACATATTTCTGTCAAACTGAAGAATTATTTGGTTCTGCAATCGATTATTCT

CGTTGCTTGTTTGGGGAAATTCCTTGGAGCACTTATCTCTTCAATCTACAACCAAGTTTCCCTCCGAGATGCAATCTCAATTGGCCTCGTATCCAATG
TTCAAGGTGTTCTAGAGCTAGGCATGTTTAAAATGATGAAGCAAAATGAGGCAATAGCGGATGAAGCTTTTGTTGTGTTGTGCATATCCCTGTTAAT
TGCAACTGCAATCGTGACACCGATACTGAAGTCCCTGTATGATCCACATAAAAGATATGCAGCACACAAGAACAAGAATATCCAGCATATGAAGCC
CCACTCTGAGCTTCGGGTGCTTGCATGTATACATGATCAAGAAAATGTTCCTTCCACAATAAACTTGTTGGAAGCACTTCACCCGTCAAACCAAAGC
CATATGGATATCGCGGTGCTTCATTTAATAGAGATGGTTGGTCGTGCACATCCTCTTCTCATAAACCACAAACTCCCATTAATGATGAAGCACACAA
ACGAGGCGTCAGCTTCAAAAAGAATCATCAATGCTTTTAAAGTGTTTGAGAAGAACTTCTGTGAGACAGTAACCATGCATCCTTTTACAGGTATATC
TCCTTATGTAATGATGCATGATGAAGTTTGCACAATGGCGCTTGAAAGGAGGGCATCTCTCGTAATGATTCCTTTCCACAAGAGGTTAACAAGTAGT
ACTTCTTCTTCTGTGAATCAGAAGAGGGCATCGAAGATGGGGATTAAGACTATGAATGACAAGATCCTTCAGACAACACCTTGTTCAGTTGCAATAA
TTGTAGACAGATCACTTGTCAACACATCAAGGCCAATACTAGACGCGTGGTCATTGTACCGTGTTGGTGTACTATTCTTGGGAGGACCAGATGATAG
GGAAGCACTAGCTCTGGGAGAGCGTATGGCTGGAAAACAGAACATTAGCCTGACAATAGTGCGGTTAGTACTCCTTCATGAAAGTGGTAATTACAG
CAATAACAACACAAGTAGTAGCGATTATGAGACGATACAGAAGATGATGGACAATGAAATGTTAAGTGAAGCAAGGAGCGATATGGCAGGAAATT
ATCGTGTCAAGTATGTGGAGAAGTTGATAAGAGATGGAACAGGTACAGCTGCTGTTATGCGATCCATGGAAGACGAATACGAACTAATCATTGTTG
GGAGACGTCATGACTCACAATCTCCTCTATTACTTGGTCTTTCTGATTGGGTTGAAGAGTCTGAATTAGGCCCTGTAGGAGACATGTTTGCTTTAGCA
GATTCACAAAGTAATTCCACAATATTAGTTGTGCAACAACACAATGGAGGATGA
>PG0008849
ATGGGTTTTAATGTAACCAATTCCATAAAAACATCATCAGATGGAGTATGGCAAGGAGAAAATACTTTACACTATGCTTTTCCATTATTAATTATTC
AAACAACGTTAGTTGTGTTCCTTAGTCGATTACTTGCTTTCCTTCTCAAGCCCCTCCGCCAACCTAAAGTCGTCGCGGAGATCCTCGCAGGGATTATG
CTTGGGCCATCAGCATTTGGGAGGAACAAAACATTCACAAATTGTATTTTTCCATCATGGAGTACTCCAATTCTTGAATGTGTATCAAACATAGGCC
TATTATTTTTCTTATTTCTTGTTGGTCTTGAATTGGACTTAAACACCATACATAAAAGTGGAAAAAAAGCTATTGGAATAGCATTTGCTGGAATTTCA
TTGCCCTTTCTATTTAGCATTGGAGTTGCTTTTGTTCTAAGAAAGATAATCAAAGGGATTGATAGTGTTGGATATGGAGAATTTTTCTTGTTCATTGG
TGTTTCACTTTCAATCACTGCCTTTCCTGTTCTTGCTCGAATCCTCGCGGAATTAAGGTTACTGACTACACAAATTGGTGAAATGGCTATGGCAGCAG
CTGCATTCAATGATGTTGCTGCATGGATTTTACTTGCTCTTGCAATTGCTCTAGCAGGAGGAGGAGGAGTTCATCATAGTCCTTTAATTTCACTATGG
GTTTTTCTAAGTGGAATTGGATTTGTGGTTTTTATGTTCCTAATTATTAGGCCAATTATGATTTGGGTGGCAAAAAAATCATCAAATGGGAACAACA
ATATTGTTGAAGAAACTTGTATATGTTTAACCCTAGTTGGAGTTATGTTATTTGGTTTTATGACTGATTTTATTGGTATACATGCAATATTTGGAGGA
TTTATATTTGGATTAATTATTCCAAAAAATGGGGACTTTTCAGAAAAATTGATTTTGAGAATTGAAGATTTTGTGTCTGGATTATTATTACCACTTTA
TTTTGCATCAAGTGGTATTAAGACAAATATTTCACAAATTCATAGTGTTAAGGCTTGGGGACTTGTGGTTCTTGTTGTGTCCACAGCTTGTGTAGGCA
AAGTTTTGGGGACTTTTGTTGTGGGAATAATGTTGTGTTCAATGCCAATGAGAGAAGCTTTAGCACTTGGATTTTTGATGAATACAAAAGGGTTGGT
GGAGCTAATTGTTCTAAATATTGGAAAGGAGAAAAAGGTTTTGGACGATGAGACGTTTGCAATATTGGTGATCATGGCACTTTTTACAACCTTTATC
ACAACTCCTATAGTAATGGCTATTCATAAACCATCATCAACACAAAATCCTCAACTCGAAAAACCACAAAAAAAGATCAAAAAACAAAACAATCTT
CGAATCCTAGCGTGCTTACGTGGCCCGAGGGATGCACGTGCACTCATTAACCTCATCGAGTCACTAAGGTCTGAAAAAAATAACAATAACTACGCC
TCAGTCACAAAACTCTATGTGATGAGACTCGTGGAGTTCACCGACCGACTCTCATCCATATCGATGGTCCAACGTGCCCGAAAAAATGGTTTTCCGT
TCATCGGCCGAGTGCTTTTCCGAGATGACGCGACCGATCAAGTGGGCGCGGCGTTCGAGGCGTATAGCACGCTAGGAAAGGTCATAGTCCGACCCA
CAATGGCTATCTCGGGTTTATCGGATTTGGATGAGGATATTATTCATGTTGCGGAGAAAAAACGAGTTGAACTGATTATTTTGCCGTTCGATAAATA
TTGGCAAATGGAAGGAAATGAAGAAGTGGAGATTCATGCAGGGCACGGATGGAGGATGGCAAATGAGAGAGTGATGAGTCAAGCGTGGTGCTCGG
TGGCGGTGGTGGTTGATCGTGGATTACAATTGGTTGATAATGGGATGAGAATTTGCATTGTGTTTTTTGGTGGAGCTGATTGTAGTAAAGCTTTGGA
AATTGGTAGTAGAATGGTTGAACATCCTGCTATTAGGGTTACATTAGTAAGATTCATTCATCATGGAAGTACTAATTTTGATGAAGTTGAAAGGACT
TTGGATGATTCTACTATTGCAGAATTCAAGATGAAGTGGGGTAAACAAATTGTATATAGTGAGAAAGAAGCAAATAATTTGGTGAATGAAGTGTTG
GAAATTGGAAAAAGTGGAGAATTTGAACTTATGATAATAGGGAATAATAAAAGTAAGTTTCCACAAGGCATAATGGCAAAACTATTTGATGAGCA

ACAATTGAATAATTCAGAATTTGGGCCATTGGCTAATTTGTTGGCTTCATCAGACAAAGGGATAAAAAGTTCTGTTCTAGTGATTCAACAACAACAA
CAAGAAGCTAAGTTTGGGAACTCAAAAGTGGCTAGTAGCTTTATTGATAAGGATATTGTGTGA
>PG0004171
ATGTCAAAGTTGAACTTGACAGAAGACCCTTACCTCCTTAGCAAAGGGCTAGTAATATGTCGGGGTATCCATCCTCCACACACCTTTGGTATTTTTTC
AGGAGAAAATCCATTGGAGTTTTCTTTTTCCCTTGTCTTGTTAGAGATATCAACCATCATTGCCATCTCTCGTTTCATTCGTTATCTTCTCAAGCCTCT
CCGCCAGCCAAGAATCATTTCTGAGCTTCTAGGTGGAATAATCATAGGCCCATCTGTTTTGAGCCGAAGCAAGGGATTTCGCAACTTTATCTTTCCG
GATACTGCTGATTATGCACTCAAAAATATTGGACTCATTGGTTTTATGTATTTTCTTTTCATATCTGGTGTAAAGACAGATTTGACACAGATAAAGAA
TGTTGGCAAGAAACAATGGTACATAGCCATATTTGGGGTGTCCATTCCCATGTTATGTAGTCTTTTTATTGGATTAGCTCTTCAAAAATCAATGGAAA
AAGAATTAGCCAAAGCTTCATCCATGTTAGGAGTAACGTCAGAATTGGCAATCACGGCTTTCCCAGTTATCTATCCAATTATCAGAGAGCTTAATCT
CCTTAGCTCCGAAATTGGAAGAATGTCACTATCCACTGCCTTAATAAGTGATATAATTGGAATTCAATTTGTTGTTATATTTGAGGCAGCTAAACAA
GGGGAGCATAAAAGTATGGCTGCCTTGTGGTTTCTCATTTATTCATTTTTGATTGGAGCATCCATTTTTGGAGGTGTTAGACAGATTATGATATGGAT
AATAAAAGCAACCCCGGAAGGGAAGTCAGTGGAGCAAATTTATGTTGTTTTCATACTCTTGGGAGTATTGCTTACTGGTTTCTTATGTGATTTGGGT
GGTATTGCAGTGGCTAATGGACCACTTTGGTTGGGACTCGCCATTCCAGATGGCCCACCATTAGGAGCTACATTGGTCGAAAAGACAGAGACAATT
GTCATGGACATTCTCATGCCATTTTCATTTGCATATGTTGGAATGTTCACTGATATTTCATCTATCTATACTCATTGGCCACATCTTCAGCCTATATTT
TTTATGGCTCTCACAGCTTATCTTGTTAAAATGGTTACTGTCCTCTTTACTTCCTATTTTTTTAATATGCCATTCAGAGATTGTCTTGCTCTTAGCCTTG
TATTGAGCCTAAGAGGTGAAGTTGAGCTTTTGATCTTCATTCACTGGATGGATTTGAAGATGATAACGAGACCATATTTCACAATGCTAGTGCTAAT
GACAATTGGGGTGACATCTATTGTGACTCCCTTGATCAGTATGGTTTATGATCCAACGAGGCCTTATATGATTAACACAAGAAGAAACATTCAACAT
ACTTCTTTAAACACTGAGTTGAACATTATAGCTTGTATACATGACGAGGAAAATGTGCCTGGTATAATAAATATTCTATTTGAAGTCTCTAATTCAAC
TGCTCCTAGGACTTCCATGGTTCATGCCTTGCACCTAATGGAACTAGTTGGTCGTGCTGCCCCTATTTTTATCGATCACCAAGAATCGGTAAATATAG
ATCAAAATCCAATCCATAATGCATTGAAGCATTTTGGTGGAGAAAATATCACGATTAATTCGTATACATCATATTCTCCTAAGAGGAGCATGTATCA
AGACATATGCAAGTTGGCACTAGAGAAGAAAGCCTCCATTATTATACTTCCCTTCTATAAAGGCACTCAAGTACTAACAAGACAAGGGGTTCAATT
AGTGAACTCTAATGTCTTAAACCATGCTCCTTGCTCAGTTGGGATTTATGTGGACCAGGGTGCTTCCCCTACTAGTAATAATTATAATGTAGGTAGAC
GGTCATCAATTAACAAATTTGCTCTACTTTTTATGGGAGGATCAGATGCTAGGGAGGCACTTTCTTATGCTGATAGAATTGCTGCAAATCCAGATGT
ATCACTTACTGCTATTCGTTTCCTTTCCCATAATGGTGAAGGGGATAACGAGATGGAGAAGAAATTAGATGATGGTCTTGTAACATGGTTTTGGGTG
AAGAATGAAGGTAATGAAAAAGTTGTGTATAGGGAAGTTGTGGTGAAGAATGGGGAGGATACAATCGCAGCACTTCACACATTGCAGAATGAAGA
ATTCTTTGATCTTTGGATACTTGGGAGAAATCAAGGCATAAATCCTGTGCTATTACAGGGATTAACACATTGGAGTGCTCAAAATGAACTAGGAGTT
ATAGGAGACTTTCTTGTCTCAATGAATTCAGGTACCACAACTTCTATTTTAGTGATGCAACAACAAATTTTAAGAGGCCAAGAACCCACTTCTCTTG
GT TTTCTAAAAAAAAATTGCAACTTGTCGATAG
>PG0031029

ATGGAGAGGATTGTGGTGAGAAGAGAGAAGCTTCGAGCATGGATATTCCTCTTCCTGTCGGTTGTTTATTCTGGGAGGATGACCTTAGCGGCCAGAT
CTGAAAAGGAGATTAGGGCAAGGTTTACGGTAATTTAGTAAATTCGTCCGCTCCCGTAACTAATGATGGCACTATTGCCAAAATGTTCGATCGTGTT
CTCGAGAAAGAGTTTTCTGAAAACGATCAGCCTGAAGGTTCTGATGGACGCAGCTTTAACAGTACGGTTGCTGATGAGACGGGAGTCCTGGAGACG
GTAGCCAAAATTACCCATGAGAAGATCAAGAAGAATGAGACACAACAGACAAATGATACTAGATCATTCAAATTACAAGACGTTTTCTCCCTTGAA
AATGAAGGTTCTGATGATGTTACTACATTGATTGACAAAAAGGATAATGTGTTTGTCATGTCAAATAAGAAATCAAAATATCCGGTGCTTCAAGTTG
ATGTGAGACTTATCTCAGACTTGGTGGTGGTTATAGTCTCTGCTGCCATTGGTGGAATCATCTTTTCTTGTTTAGGACAACCGGTTATTGTCGGTTAT
CTTCTTGCTGGCTCATTAATTGGACCAGGGGGTTTGAAATTCATCAGTGAAATGGTACAGGTTGAGACCGTTGCTCAGTTTGGAGTTGTCTTCCTTCT
ATTTGCTTTAGGTCTGGAGTTTTCCTTGACAAAGCTAAAAGTTGTTGGTCCTGTTGCTGTTCTTGGAGGGTTGCTTCAAATTGTTATCCTTATGTTTCT
GTGTGGCACAACTGCAATGTTATGTGGTGCAAACTTGTCAGAGGGAGTGTTTGTTGGTTGCTTCCTATCAATGTCATCAACGGCAGTGGTGGTGAAA
TTTCTGGTTGAGAAAAACAGCAACAATGCTCTTCATGGTCAAGTGACAATCGGAACATTGATTTTTCAGGACTGCGCTGTTGGCTTATTATTCGCTTT
GCTTCCTGTTCTTGGAGGTAACAGTGGGCTTTTGCATGGAATTATCTCTATGGGAAAAGTGCTGCTGATCCTATCCATGTACCTCAGTGTTGCATCAA
TTTTAACTTGGTCATTTGTTCCACGTTTTCTGAAGTTAATGATTCGGTTATCTTCTCAAACCAATGAATTATATCAGCTTGCGGTGGTGGCATTCTGCT
TATTATCTGCTTGGTGCAGTGATAAGCTGGGCCTTAGTCTTGAGCTGGGTTCATTTGTAGCCGGAGTTATGATATCCACAACTGACTTTGCAAAACAT
ACCTTGGACCAGGTGGAACCAATCCGTAATCTCTTTGCAGCTCTCTTTCTCGCTAGTATTGGAATGCTGATACATGTACAGTTCCTTTGGACACATGT
GGATATCTTGCTTGCCTCCGTTATCCTGGTTATAGTTTTTAAGACTACTGTAGCCACTGTGATTACAAAGGTTTTTGGATATAACATTAGGACGTCAC
TTATTGTTGGACTTTTGCTTGCACAAATTGGGGAATTCGCATTTGTGCTCTTGAGTCGTGCCTCGAACCTGCATATTGTTCAGGGCAAGATGTACCTT
CTTCTTCTCGGAACAACTGCTCTAAGTCTTGTCACAACCCCCGTCTTGTTCAAGTTGATACCTGCTATCATGCACCTGGGAGTTCTTATGCACTGGTTT
CCTGTGGAAAACGTCGCACCAGATGAGGAGAAAGTTGCAATGATTGTTGATACGCACAACAGAGTATTGTAA
>PG0018689

ATGGCTGCTGCTACAGAGCAAATGGCCGCAGCGGGTTCTTGTAATCAAGAATTGTTCAATCCTATTATTAGTATGGGATGGCAATATTCTTTGATTCT
TGTGATTTCTCATATTCTTCAGATTTTGCTCAGGCCATTAGGACAAGCTTCACCCATTGTTCAAATTCTCGCGGGGTTCTTGATGGGTCCCTCGGGGT
TTTCGCGAATCAAAGCAGTAGAGGAGTTCTTCATTCAAAGTTATAATTCGGGTTACTATGAATTCATGGCGTTAATTTTTAGAACTATAATCATGTTC
CTGATTGGTCTTGAGACGGACTTCCCTTATCTTATGCGCAACATACGCCCTGCAAGCATTATTGCATGTGGCAGCAGTTTAGGATGTACTGTCTTTGC
AAGTGCTGTCACTTTCTTAGTATTTCAAGAAACAGCTTCCCATGGTTCTTCATTCATAATGGCCTTAATGATCATAATAACCTTAGCCAATGCAGCAT
CACCGATTGTTGTCCGCGTGGCAGCGGACCTTAAGTTTGGAACTTCTGAGACTGGCAAGTTGGCTATATCTTCTTCCTTGATAGCCGATGCATATTCG
GTGTTTCTATTGTTTATACTTTCAGAGTTCAAGTCAACTTCAATTGCAAAATGGATCTTTTTCTTTTTCTCTATTTTCTTATTGTTGGTGTAGTTATTG
TAATCAATATGTATCTAGCTAATTGGTTGAATAGGAGGAATAGGAACAAAAAGTACCTTGGAAATACTGAAATATTTATACTTGTAGCGATTCTCTA
TATCGCTGCTATGGCTCTCGAACAGCTTGGATTCAGCAGCATTATAGCTTCTTTCCTCATTGGCTCAATGTTCCCTAGAGGAGGGAAAGCAGCTCGA
ACTTTGTTGATCAAACTCACATATCCAATTCACAATTTCATATTTCCAATCTACTTTGGCAACCATGGTTTCAGGGCAAATATAACTAAGCTGAAAAA
TCTGCGTAACTTTATGGTATTCTCTATCCTTATTTTGTCAAGCATCGGAGGGAAGATTGTTGGAACACTGGCAGCTTGCTTTCACCTAAAGATCCCTT
ACAGAGAGGGGGTGCTTCTTTCTTTTATGATGAATCTAAAAGGTCATGTTGATATTCTAGCATTGACGATTGGCTTGGCGAATGACCTTGTTTCGAGC
CAGAATTTCTATGACGTGATGATAGCAACAATTATTGTGAATACATTGATATGGGGACCAATAGTAGCTTTCATGGTGAGAAGAGAAAGTGATATC
ATTGGCTACAGGCAAATATATTTTGAATCTCATAATCCAGAAACCGAACTACGAATACTTGCTTGTGTGCATAGTCCGCGACCAGTGGCAACTATGC
TAGGACTTGTTGCAGCCTCCAGAGGGCCAAGAGAAGTTCCAATAACCCCTTACTTGATGCATCTCGTTGAGCTGCCAGGTAAAAAAAAGACTAATTT
GATGTACAATCAGCGAGAGGACGATGAACTAAGTGATGAAGATGATTATGGTGGGAATGATGTTGTTGAGATAAATGATGCCATGGATATGTTCAC
TAGTGAGACAGGGTTATTGGTTCAACAGATTAAAGCTGTGTCTCCGTTTTCACGTATGCATGCAGATGTTTGTAACACTGCTGAAGACATAAGAGCA
TCTATCGTTGTCCTTCCTTTCCACAAACACCAGAGAATCGATGGGAAGCTAGAGAACGGTAAACAAGGCATACGAACTACAAATCAGAAAGTTCTT
CGTCATGCCCCATGTTCAGTTGCCATTCTTATTGACAGGGGACTTACAGCCGGGTGCTTAAATCCTTCGGGTTCTGACTCTCTGCAACACATTGCTAT
CTTATTTTTTGGCGGACCTGATGATCGTGAAGCATTAGGCTTTAGTAAACGTCTTGGTATGGATCATCACGTAAATCTCACCATAATTAGGTTCCTTC
CCTCGTCTTCAAGAGGACAAATTTCAGGTGTCAATATTGCTCACAAAACGGATGATGTCTTGATGGCAATACCAAATGACGAAGTAGAGAAAGAAA
CAGATAGCGCAATTTTGGCAGATTTCCATAGCAGGTATGTGGCGACAGGTCAAGTAGGATATGTGGAAAAGGTTGTAGAAAATGGCGCAGACACA
GCGTCTGCGTTGAGAGACATGGCTGAAATGTATTCATTGTTCATAGTAGGGAAGGACGGGAGAGGACATTCAATACTAACAACTGGAATGAGTGAT
TGGGAAGAATGTCCAGAGCTCGGTAAAGTAGGCGATTTTTGGCTTCTCCAGAATTCGATATTAGTGGTTCAGTTCTTGTTGTTCAGCAGTATAGAC
CTTCAAAGAATGATGACGACGATGACGATGACAAATAA

>PG0030154

ATGGCAGATCCAGAGCCATTGATTGATGTTGGAAAATTAAATGAAAAAGTTCTATGTTATGCACAAACAATTTATAGATTTAATGGAGTTTGGGAA
GGACCAGATCCTTTGACACCAATTATCCCTCTTTTCTTTATTCAAGTTTCATTAGCTATATTGATCACTCGTTTTGTCACCTTTGTCTTAAAACCCACC
AAGCAACCTCCTTTTGTTGCTGAGATTATTAGTGGCATACTTTTAGGTCCTACAGCACTTGGAAGGATCATGAGGTTCAGGAGGCTGCTTTTCCCAA
ACTACAACTTCCATGTTATTGAGACAATGGCTCATGTAGCCCTTGTGTTCTATGGGTTTCTAGTGGGATTGCAGATGGATATGAAATCCGTTCTTCGA
ATTGGAATAAAGGCTAGAAATGTTGCTATTATCGGTATCATCATCCCTTTTGTCATGGGAACAATATTATATTTCTCACTTACTCGCGATGAAGAAGT
TAGAGGCTTCATTTTCTATGGTGGTGCTCTTACTATAACAGGGTTCTCTGTCCTGTCTAAGATACTTGATAAACAAAAGATCCTCCAAACTGACATAG
GGAAAATGGCTATGTCTTCAGCTGTAATCAATGATATTGGTGCATGGTTTATTCTTACACTAGGATATGTTGTCACAGGGAGTACAGCTAATATCCA
TTGGGCATTAATTTGCACGATTGCTTATGCCTTGTTCTGTGTCTTTTATCTTCGTCGTGCCATTGGCTGGACCATTAGAAAAATGCCAGAAGGACAAG
GGTATAGTGAGTTCTTTATATGTTCAATCCTTGCTGGAATGGCTATTTCCGGAGTTATAACTGATGCTTTAGGGACACATCCTATTATTGGAGCTTTT
CTCTTTGGACTTAGTATACCTAATCAAGTGCTTCAAGCAGAAATTATCGATAAGCTTGATGACTTTGTGACGGGCATTTTTATGCCAACTTTCTTTGT
TGTTTGTGGACTCAGAACCAATTTCGGACAAATGGGCAGCATTTACGAAATTGTTGGCTACATACTTTTATTTGTTTCAGCCAAAATCTTGAGCTCAA
TAGCTGCCACTTTCTTCTCTGAAATGACTATTAAGGAGGCCCTGGCTGTTGGAGTACTTAGCAACACCAAGAGTATCATGGCCTTGATCATTATCGA
AGCTGGTCAGGCACAACAGGTTTTGAGCACGCAATTGTACTCCCTTATGGTGGCTGGTATTTTGGTGATGACGGCGATAGTCACACCTATGACTATG

CTCCACCGTCCCTCGCAGGAAATTGCTCCCCATAAACGAAGGACTATACAGAAGGCAAGAATGGAGGAGGAGCTTCGGGTCCTTGCTTGCATCCAT
GGCACACATGACATCCCTTCAGTCATCAACCTTCTTGGCTCGTCACATTCTACGCCAGCATCCCCAATAACTGTCTTTGCTCTCCAAGTAGTAGAATT
AGTCGGGCGTGGATCATCTATGCTAGAAGTACACAACTCAGGGAAACGAGGCTCGCGAAGTCTTGGACACGAGGAGACACAAACAAGACAGATCA
TCACTGCTTTCGACAACTATGAGCTACGATCAGATGGAGTGATGGTACAAGTACTCACAGCAAGGTCTGCTTTGTCCACCATGGATGAAGATATGTG
CAACATTGCCAAAGATAAGCGTGTAGCTTTCATCATTCTACCTTTCCACAAACAACGAGGTATCGAGGGTGAAATGGAGGATGTCAACCCTGAAAT
TCGGGCTGTCAACGAAGGTGTGCTAGCTAATGCCCCTTGTTCTGTGGGAATCCTCATCGATCGTGGCCTCTCTGAAACAAGTGACTATGCAAAAAAT
ATCGTTGTCCTCTTTTTCGGAGGTGCTGATGACAGGGAAGCTCTAGCTTATGCTTTGAGAATGGTTGATCGTCCAGATACACGCCTAACTGTTGTAAA
GTTCATTCCAGACGAAGGTGCTTCTGATATAGAGCAAACGGAATTTGCTGATGAAAGTCATGTGAATGTCCAGATTGACAAAGAGAGTGAAAAGTT
GATGGACGACGAGTTCTTAAACAGGTTCAAGATCAGCACAGCCAATGACAAATCAGTAACATACATAGAACTGTTGCTGAATGATGTGGAAGAAGC
TGTCAAGGCCATAAAACTAATGGATCAGCATAACTATGATCTCTATATCGTAGGAAAAGGCCGAGGTGTGGTTTCACCACTAACAGCTGGCCTAGT
CGATTGGTGTGACTGTCCTGAGCTCGGGGCTATTGGTGATCTTTTAGTGACATCAGAATTCGACTCCACATTCTCCGTGCTGGTGATGCAACAGTATG
TTAAGCCAATTGGAGATGGTTCAGTAAATTCTTATGGATCAATGAGTGAGAGGATAGCAGGCATTGGGAATATGGACATGGACTTGGACATGCAAC
GCGCGGATAGTGAATCAGGGGACGTGTTCTCCAGTTTTAGAAGGCGAACGGAACATATGCCACGAGTCTAA
>PG0035252
ATGGCATGTCCAAAACCTATGAAAGCTACATCTAATGGAATATTCCAAGGAGATAATCCATTAGACTATGCACTTCCTCTTGCGATTGTACAAATAT
GCTTGGTGCTTGTACTTACTCGAGTCCTCGCCTATATTCTCCGTCCTCTAAGACAACCTCGCGTCATTGCTGAGATTATTGGAGGAATTCTACTTGGT
CCATCTGCTCTTGGCAGGAACCTGAAGTATCTGAATGCAATATTTCCACCAAAGAGCCTCACAGTGTTGGATACTTTAGCAAACTTTGGCCTCCTCTT
CTTTCTTTTCCTTGTTGGGCTCGAGTTAGATCCAAGGTCTCTTCGTCGTACTGGAAAGAAAGCTCTTAGTATCGCCCTCGCGGGAATTAGCCTCCCTT
TTGGACTAGGAGTAGGGACTTCATTCATTCTTAGAGGAACTATTGCTAAAGGTGTTGGTCAAGGCCCTTTTCTTGTCTTCATGGGAGTAGCTCTTTCT
ATCACTGCCTTCCCTGTCTTGGCGCGGATTCTAGCTGAGTTAAAGCTCTTAACGACTGATGTTGGTCGAATGGCTATGTCTGCTGCAGCCGTGAATGA
CGTGGCTGCTTGGATTCTACTTGCACTTGCTATTGCCCTTTCAGGTACTGGTCATTCACCTCTTATTTCACTATGGGTACTTTTGTGTGGGACGGGATT
TGTCCTGCTTTGCATAGTCCTTGTTCCTCCGATATTCAACTGGATGGCTAAACGTTGTCCTGAGGGTGAGCCGGTTGATGAGTTATACATCTGTGCTA
CACTTGGAGCCGTGCTGGTTGCAGGATTCGTTACTGATGCAATTGGTATTCATGCCTTATTCGGGGCTTTTGTGCTTGGAATTCTTGTACCAAAGGAA
GGGCCTTTTTCTGCTGCTCTGGTGGAAAAAGTTGAGGATCTTGTTTCTGGTTTATTCCTTCCATTGTACTTCGTCTCCAGTGGACTGAAGACGAATGT
AGCTACTATTCAAGGGGCACAATCATGGGGTCTTCTTGGTTTAGTCATATTCACATCGTGTTTTGGAAAGATCGTTGGCACTTTTTTGGTCTCGCTAT
TGTGCAGAATGCCTGTTCAGGAGGCTGTTACTCTTGGTTTCTTGATGAACACTAAAGGTCTAGTGGAGCTTATTGTCCTCAACATTGGAAAAGATAG
AGGGGTACTGAATGATCAAACATTTGCCATCATGGTTCTGATGGCACTCTTCACAGAACGATTGAGAGGAAAGACACGAGCAAAGAACTCCGAATC
ATGA
>PG0012169
ATGGCTTCAAATGGAACTATGAAGTGTCCATCACCAATGAAAGCAACATCTAATGGAGTTTTTCAAGGGGATAATCCATTGGATTATGCACTTCCTT
TGGCTATTGTTCAAATATGTTTAGTACTTGTGCTTACTAGACTTCTTGCTTATATTTTAAGGCCATTGAGACAACCTAGAGTCATTGCTGAGATTGTT
GGAGGGGTTCTACTAGGCCCATCTGCTCTAGGACGCAACCAGAAGTATCTACACGCTATATTTCCACCGAAGAGCCTTACAGTGCTAGACACCTTAG
CCAATTTTGGACTCCTCTTCTTTCTTTTCCTTGTTGGACTGGAGCTAGATCCAAAGTCCCTTCGTCGGACTGGAAAGAAAGCTCTAAGCATTGCTCTT
GCAGGAATCAGTGTTCCTTTTGCATTAGGAATTGGGACATCCTTTGTTCTCCGGGAAACTGTTTCTAAAGGAGTTAACCAAGGCCCTTTTCTCATCTT
CATGGGAGTAGCCCTTTCCATCACTGCCTTCCCCGTTTTGGCTCGTATCCTGGCTGAACTCAAGCTTTTAACAACGGACGTTGGTCAAATGGCGATGT
CTGCTGCAGCTGTCAATGATGTGGCTGCATGGATTCTACTCGCTCTAGCTATCGCCCTCTCAGGTGTTGGTCGTTCTCCTGTTATCTCGTTATGGGTCC
TTTTGTGTGGAACTGGTTTCGTGCTACTCTGCATATTCATTGCTCCTCGTATATTCAAATGGATGGCTAGACGTTGTTCTGAAGGTGAGCCAGTGGAC
GAAAAATACGTGTGCGCTACACTTGCAGTTGTTTTGGCTGCATCATTTGTGACTGACATGATTGGTATTCATGCATTGTTTGGGGCTTTTGTGCTTGG
AGTTCTCGTCCCAAAGGAAGGGCCTTTCGCGGGTGCTCTGGTGGAAAAAGTTGAGGATCTCGTGTCTGGTCTATTCCTTCCTCTGTACTTTGTGTCTA

GTGGATTGAAAACGAACGTAGCCACCATTCAAGGGGCTCAATCATGGGGTCTTCTTGTTCTAGTCATAGTTACAGCATGTTTTGGGAAGATTGTTGG
CACTATCGTGGTGTCACTACTGTGCAAATTGCCCATCCAAGAGGCTGTAACCCTCGGTTTCTTGATGAACACTAAAGGTTTGGTGGAGCTCATTGTTC
TCAACATTGGCAAAGATAGAGGAGTACTTAATGATCAAACATTTGCCATCATGGTGTTGATGGCTCTCTTCACGACGTTCATCACAACACCTATAGT
GATATCAGTATATAAGCCAGCAAAACTGGCTGTGACAGCATACAAACACAGAACTATACAGAGGAAAAACACGAGCAAACAGCTCCGAATCTTGG
CCTGCTTCCACAGCACAAGGAACATTCCTGCAATGCTCAATCTCATTGAGGTTTCTCGGGGTATTGAGAAGAGGGAAGGGCTTCGTGTCTACGCAAT
GCACCTCATGGAGCTTTCAGAAAGATCCTCTGCAATCCTGATGGTCCACAAGGCTAAAAAGAATGGATTGCCCTTTTGGAACACCGAACAGGTGCA
AGACTCGAATCAAATTGTTGTAGCTTTTGATACATTCTCAAATCTGAGTAAGGTATCTATTCGACCAACTACTGCAATCTCTCCTATGAACAGCATGC
ACGAGGACATAGTCGCTAGTGCAGAGAGAAAAAGGGTTGCAATGATAATCCTCCCGTTTCATAAGCACCCGAGACTCGATGGACATTTGGAAACAA
CCCGAGGCGAACTTAGGCATGTGAACAGGAGAGTTCTTCAGCATGCACCATGTTCAGTTGGCATATTAGTAGATCGTGGTCTCGGTGGAGCTTCTCA
TGTATCCTCAAGTAACGTTGACTTCTCAGTGACTGCCTTGTTCTTCGGTGGCCATGATGATCGCGAAGCACTTGCTTATGGTGTACGTATAGCTGAAC
ACCCTGGCATTAGTTTGATTGTGGTTCGGTTCATAGTAGACCCTGAGGTTTCCGGGACAAGTGTCAAGGTAGAAATGAATGACAAAACTAACCCCG
AGGCTCAATCTGATGATGAAGAGTTCCTTGCTGATGTTAAACAAAAATCTTCAATAGATGGATCGATCAAATTTGAGGAGAGGCTCGTGAAAGATG
CTCGTGGGACTATAGAAGCAATTCGTGAGTACAATCGTTGCAATCTGTTTCTGGTTGGCAGAATGCCTGAGGGACAAGTAGTTGTAGCATTGGACAA
AAAGAGTGACTGCCCTGAGTTAGGATCCTTGGGAAACTTATTGACATCACCAGAATTTTCGACTACAGCATCAGTGTTGGTGGTGCAGCAGTACCGC
AGCCAGTTGCCTGAAGAATCTCTCAGTTCTTTGAAGGAAGGAGAGTCATCAGACGGCGATTGTGATTCAGAATAA
>PG0012168
ATGGCTACACCTGCATCACCCTTGACTTGTCCAAAGCCCATGAAGGCTACCTCTAATGGAGTATTCCAAGGGGATGATCCATTAGATTATGCACTTC
CTCTTGCCATTGTGCAGATATGTTTAGTACTTGTACTCACCCGAGTCCTTGCCTATCTTCTTCGTCCATTGAGACAACCGCGTGTCGTTGCTGAGATTA
TTGGAGGAATTTTACTAGGTCCATCTGCTCTAGGCCGCAGTGAGAAGTATCTGCACACAATATTTCCACCAAAGAGCCTGACAGTGTTGGATACTTT
AGCCAACTTTGGCCTCCTTTTCTTTCTTTTCCTGGTTGGGCTGGAGTTGGATCCAAAGTCTCTCCGTCGGACTGGAAAGAAAGCTCTATGTATTGCCA
TTGCTGGAATCAGTGTCCCCTTTGTATTAGGAATAGGAACATCCTTTGCTCTGAGAGCAACTATATCACAAGGGGTTAATCAAGGCCCTTTTTTGGTG
TTTATGGGAGTGGCCCTCTCTATCACTGCCTTTCCTGTATTGGCTCGTATTCTAGCCGAACTCAAACTTTTAACAACAGATGTTGGTCGAATGGCCAT
GTCTGCTGCAGCAGTCAATGATGTGGCTGCATGGATTTTACTTGCTCTTGCCATTGCCCTCTCAGGTAATGGTAGTTCACCCTTTATTTCGCTTTGGGT
CCTTTTGTCCGGGGCTGGTTTTGTGCTACTCTGCATACTCATTGGTCCTCCTATATTCACCTGGATGGCTAAACGTTGTTCGGATGGGGAGCATGTAG
ATGAGATATATGTGTGTGGTACATTGGCAGCAGTTTTGGCTGCAGGATTTGTCACTGATAGTATTGGTATTCATGCCCTATTTGGGGCTTTTGTGCTT
GGAGTTCTTGTACCAAAGGAAGGACCATTCGCAGGCGCACTAGTGGAAAAAGTTGAGGACCTTGTATCCGGTTTATTCCTTCCACTCTACTTTGTCT
CCAGTGGACTGAAAACAAATGTCGCCACTATTCAGGGGGCTCAATCATGGGGTCTTCTTGCTCTTGTTATATTTACATCATGCTTCGGGAAGATTGTT
GGCACGATTGTGGTCTCACTCCTCTGCAAGATGCCTGTACAAGAGGCTCTGACTCTTGGCTTCTTGATGAACACTAAAGGATTAGTCGAGCTCATTG
TTCTCAATATTGGCAAAGACAGAGGGGTACTGAATGATCAAACATTCGCCATCATGGTGTTGATGGCTCTCTTCACAACATTCATCACAACTCCTAT
TGTGATATCAATATACAGGCCAGCTAAACTAGCTGTGACTAAATACAAGCATAGAACCATAGAGAGGAAAGACACGAGCAAACAAGTCCGAATCT
TGTCCTGTTTCTACAGCACAAGAAACATTCCCACACTGATCAATCTCATTGAAGTTTCTCGTGGAACTGCTAAAAAGGAAGGACTTCGCGTCTATGC
CATGCACCTTATGGAGCTTTCTGAAAGGTCATCAGCAATCTTGATGGTACACAAGGTTAAAAGGAACGGTTTGCCCTTCTGGAATAAAGGGGAGGT
TTCGGATTCTAACCAAGTAGTGGTTGCTTTTGAGACATTTGAGCACCTTAGCAAAGTATCCATTCGACCAACAACAGCAATCTCTCCGATGAACAGC
ATGCACGAGGACATAATCACTAGTGCTGAGAACAAGAGGGTTGCCATGATAATCCTCCCGTTCCACAAACACCAGCGACTTGATGGACACTTTGAA
ACCACCAGAACTGATCTAAGGCATGTGAACCGCAAAGTTCTTCAGCAAGCACCATGTTCAGTTGGCATATTAGTAGACAGAGGTCTCGGTGGAGCA
TCTCATGTACCAGCTAGCAATGTTAACTTTACAATAACTATCTTGTTCTTTGGAGGCCATGATGACCGTGAAGCACTTGCTTATGGTATCCGCATGGC
TGAGCACCCTGGTATTACACTAGTTGTGGTACGTTTTGCTGTTGACCCTGCGCTTGCTGGGGGCAGTGTCAAGCTAAAGATGAGCCAGAACTCCAGT
CCCGAGGTTCAACCTGAAGATGAAGTTGTTATTTCCAGGTTGAAAGAGAGTATCTCAACGGATGGATCAATCAAGTATGAAGAGAGGACAGTTAAG
GATGCTACAGAACTTATTGAAGCAACAAAATCATATAACAAGTGCAATCTATTCCTTGTTGGCAGAATGCCTGAAGGACAAGTTGTTGCATCTTTGA

ACAAAAACAGTGAATGTCCAGAATTAGGACCTATCGGAAACTTGTTAACATCGTCTGATATCTCAACAACAGCCTCTCTTCTGGTGGTGCAGCAGTA
TCGCAGCCAGTTATCACAAGACGCGTTGAATTCGTTGGAGGATGGTGAGACATCAGATGGAAATGAATCTAACTAA
>PG0029945
ATGACTACTCGGAGGAGATCATCCTCCCGGTTGCAATCCTTATTTGTGAAGTTCTCTACTGTTTCAGCAATTACAGTATTGCTGCTCTTTCTAGCTTTG
GCATTTGCATTTGCGCTTCCAGAATCAGATCAGCTGCTTATTGGAGGTAGTAATGGAACTCGTAATGGTAGTGAATTCAGCAGCGGACCTCGGAGTA
GGCCTAAAGAGGATAGTTTTGCTGATATGATCGATCGAGCACTTGAAAAAGAGTTCACTGAAAATGATAAGGATGAAGTTAATGATGCGGGCAGCT
TCAATAATAGTGTGGCCGAGCAGCAGGCGGTGTTGGAGACTGTGGCTAGAGTTAAGCCCAAGAAAAATGACACAAAGAAAGAGGAAAAATCTTTT
CAGCTTCATCATGTTTTTAAACTTGATAATGACCATGGAGCAGAAGAAACGCCAACATTAATAGATAGGAAGGACAATGTCTTTATCATATCTAATT
TCAAGTCCAAGTATCCGGTATTACAGTTAGACTTGAGGTTAATATCAGATCTAGTAGTGGTCATTGTTTCTGCAACATGTGGTGGAATTGCCTTTGCT
TGTGCGGGTCAGCCGGTTATAACTGGATACTTACTAGCAGGATCTGTTGTTGGACCTGGAGGTTTTAATGTTGTCAGTGAAATGGTGCAAGTTGAGA
CAGTGGCTCAGTTCGGTGTAATTTTTCTTCTTTTTGCTTTGGGGCTGGAGTTCTCAACCACAAAGCTTCGTGTTGTTCGAGCTGTTGCAGTTCTTGGAG
GCTTACTTCAAGTTCTTCTTTTTATATGTCTATGTGGAATTACAGCCTCGTTGTGTGGCGGTAAGCCTTCAGAGGGTGTATTTGTTGGAGCATTTCTCT
CAATGTCTTCTACAGCTGTGGTGTTGAAGTTTTTGATGGAAAAGAATAGTACAAATGCACTTCACGGCCAGGTTACAATTGGCACTCTTATTTTGCA
GGATTGTGCTGTAGGTCTTCTGTTTGCTTTGCTTCCAATCCTGGGAGGCACTTCCAATGTTCTGCAAGGGTTAATATCCATGACAAAGTCGTTGGTCA
TGTTGCTCTCATTTTTGGCTATTTTGTCAATATTATCACGGAAGTGTGTTCCATGGTTCCTGAAGCTGATGATAAGCTTATCATCACAGACTAATGAA
CTGTATCAATTGGCTTCAGTGGCATTTTGCCTCCTTGTAGCTTGGTGTAGCGATAAGCTAGGTCTAAGCTTAGAGTTGGGTTCATTTGCTGCTGGAGT
GATGATTTCAACGACTGATCTTGCCCAACATACTCTTGAACAAGTTGAACCCATACGCAACTTCTTTGCAGCTCTTTTTCTTGCCAGTATTGGGATGC
TCATCCATGTCCATTTCCTCTGGAACCATGTTGATATCCTACTTGCATCAGTGATATTAGTGGTCATTGTTAAAACTGTTGTGACTTCTGCAGTTGTTA
AGGCCTTTGGCTACAACAACAAAACTTCACTGCTAGTTGGAATGTCTTTAGCACAAATAGGAGAATTTGCTTTTGTGTTGCTCAGTCGTGCTTCTAAT
CTTCATCTAGTTGAGGGTAAATTGTACCTGCTGCTTCTAGGGACAACAGCTCTCAGTCTGGTGACTACGCCATTGCTTTTCAAGCTTATACCTGCTGT
AGTTCACCTTGGTGTGCTGCTTAGGTGGTTCCCACCTGATAGTCCAAGTGAGTTTGGATTTAAAAGTGACAATTTCCGATCTGATAGTGCTAAACAA
AGGATTGCACTGGTATCCAAGGATCTGATTCACGAAGGTTAA
>PG0027255
ATGGATTTCTTGAAACAGAACGATACGAAAGTAGAATGTCGAAATCAAATTACCATAAGAATTGCATCAATGTCTATTTACCTCTTAGGTTTCTTCT
TCGCCATGTTCTTGTGTAACTGTGTCCATCTCCTCCTTCGTCCTATCTCCCAGCCTCGTATCATTGCAGAATCCATTGTGGGATTGCTACTTAGCAATC
TTGAATTCGTTCGTTCAAGGTTCTTAAACGATAGTGAAGTTCAACAGACATTGAATTACATTGTGGATGCTATTATGGTGTGTCACATGTTTGTTGTT
GGATTAGAGATTGATCCAAATATATTCCTCCAATTAACTTTACCAGAGGCGAAAGTCGCGTATTCAGGTGTACTTACAACTTTTGTTTTAGCATGTCT
GATCACTCCACTTCTCAATATATCTAAACAATCAAATGCTGTATTCAGTTCATGCCTCGCGATTGTTCTAGCTGGAACAGATTCACCTCTGTTAACTC
GATTGATAACTGATCTGAAGATTGGTAAATCAGACATTGGTAGATTTATTGTTGACGCGGGGATACACTCTGATGTTGTATCGATCCTGTTGATTGCT
ATTGGATTTCTCATATTTGATCCTGATAAGAACTTTCAGAATCGTAGTGTGATAATGATGTTGAAGATGATGGCGATATTGGTGTTTCAGACGTTGTT
AGCATCGAAAGTAGTACCATCTGTTATGAAATGGGTGAATAATGAAAATCCAGAAGGGAAACCTATGAAAGGATCACATTTAGTTGTGGCATTAGC
ATTTATAATCTTAATATGTAGTATGTCACCAGTTGTTGGTTATAGCAAAGTTTTGAGTGCATTTTTGGTTGGTTTGTTTATGCCAAGAGAAGGAAGAA
TATCAAAAATGATGATTGGGAAAGTGAATTATATTTTTAGGACAATTTTTTATCCATTGTTTTTCTTTTGGGTTGGAACTGAGGCTAAACTTTCTGAA
TTTGAGGCTGGTAAAATTGCTTCATGGGGTAAAATTATTATTCCATTTATAATTGCAACAAGTGGTAAAGTTGTTGGTTCTGTTGTTTCTGGATTGAT
GCTTGGATTTCACTGGCCTGAATCAGTTGCTACTGGATTGTTACTCAATATCAAGGGTCATTTTCAAGTCTACTTGGCTATTAATGCATATCGGATGA
ATGTCATAAGTATGTCAACAAGTATCGCGTTGGTGTTTGTAACGTTTCTAACCATAATATACACCCCGGTAGTCGTGGCAAAAATTATTGAACGTGC
AAGGAGACGTTCACCAACACAGAAAATGGCATTACAATGTGTCAATCCGGTGAATGAGATTCGAATATTGCTCTGCATTCGCAGTCCTCAAGATGTT
TACTCAGCAATAAACTTCATGGAAATTTCGCGAGGGCCAGTAAATCCAGGGATAATGGTCTACTTAACTGATATGATCGATTTAACAGATAAAATTG
CAGCAACATTGATAACACAAGGCGAGGGAATTGATGCTGTAACTGTGACTGACCCTACGGTAGTTGAAATGAGAGAGAAAATCACTCAAGATGTCA

ACGCTTATTTAAACGAGAACTGTCAAGGTGTTAGCTTACGACGAATGATGGCACTTTCTACCATCAATAACATGCATCAAGACATAAGTATCCTTGC
TGAGGACCTGATGGTACATCTTGTTATACTTCCTTTTCATAAGAATCAAGAGGAAGATGGACGACTTCAGGTCGGTCATACAGGCTTTCGTCATATC
AATCGTAAGGTTCTTCGAAATGCACCTTGTTCAGTAGGGATACTAGTAGACAGAGGACTTGGTAAAACAGTGATCGCGAGATCATCCATTTCCCTCA
ATGCAGCAGTGATATTCATTGGTGGAAAGGATGACAGAGAAGCACTTGTGTATGCAGGACGTGTAGCACGACATCCTGGAGTAAAGTTAACTGTAA
TTAGATTCTTGTTAGAAGCCGCGGGAGATAGTGTATCATCAAGAATTAGTAAAGCAAAAGCTAATACTAGCGAACATTTAGAAGAAATGAAGATAG
ATGATGAGTGTTTTGCTGAGTTTTACGACAAACATGTTGCTGGTGGACGCGTTGCTTACATTGAGAAATACCTCATAAATTCTGGACAGACATTTTC
GACATTAAGGTCGTTAGAAGGGCAATATGGTTTGTTTATCGTGGGACGGGGAGGGCGGGTTAATTCTGTGTTAACAGTAGGGATGAGTGATTGGGA
AGAATGTCCTGAGTTAGGTCCTATTGGTGATATTCTTTCAGCTTCTGATTTCTCAGTAACTGCATCTGTTTTGATCATTCAACAACATAGTCTTAAAG
GAGAATTAGATGGACTTCATGATGAATTCTCAATCATGTAA
>PG0034953
ATGGAACTGTTCGATTATGTGAATGATAATAGCAGCCATGAGATGGTGGTTTCGATAACGGTTTTCGTCGCGATTTTGTGTCTTTGTTTAGTGATTGG
TCATTTACTCGAAGAAAATCGATGGGTTAACGAATCCATCACCGCCATTATCGTTGGTCTCATATCAGGGACAGTAATACTTCTGATAAGCAAGGGG
AAAAGTTCTCACATACTTAGATTTAATGAAGAAGTGTTCTTCATTTATCTACTTCCACCGATAATATTTAATGCAGGATACCAAGTTAAGAAGAAGC
AGTTCTTCCATAACTTCTTAACTATTATGTCGTTTGGAGTTATTGGTGTTTTCATATCATCAAGTATTATCGCATCGGGCAGCTGGTGGATATTTCCCA
AGCTTAATTTCAATGGATTGACTATTCGCGACTACCTTGGTATTGGAGCAATATTTTCGGCAACGGATACTGTTTGCACATTGCAGGTTCTTCATCAA
GACGAGACTCCGTTACTTTACAGCCTTGTCTTTGGGGAAGGAGTAGTGAATGATGCTACATCAGTTGTTCTATTCAATGCAGTGCAGAAGATTGATG
TCGCAAGATTCAATGGCTGGTCGGCTTTCCATGTCTTTCTAGATTTTCTGTACCTGTTCTCCACAAGCACTTGTCTCGGAGTTGTTGTTAGTCTTTTAA
CTCTTTTAATTCCGTACTATGCTGGACTTATAACATCATACATTCTGAAGGGCTTATATTTCGGAAGACATTCTACCGTTCGTGAAATATCGCTCATG
CTTCTGATGGCATATCTGTCTTACATGTTGGCTGAGCTTTGGAGCCTCAGTGGAATTTTGACCGTCTTCTTTTCCGGAATCTTGATGTCTCACTATGCA
TGGCATAATGTGACTGATAGTTCAAGAATCACTACGAGGCATGCATTTGAAGCTATGTCTTTCATTGCTGAAACATTCATATTTTTGTACGTGGGGAT
GGATGCGCTGGACATTGAAAAGTGGAAAATGAGCCAACAGAGAATCAGCAAAACAAAGCAAAACAAAGCTGACTCTTTTATATCCAGTCTTGCAG
GTAGTGTTTGGACTTCAATGGGAATATATATTACTGTGCTCATGTTGATGGCTATTGGGCGTGCTGCTTTTGTGTTCCCTCTCTCTGTTCTTTCCAATT
TCATGAATCGAAATGCTACAAGAACGCCGCCAATAACGTTCAAACATCAGATAGTAATTTGGTGGGCTGGTCTTATGAGAGGGGCGGTTTCTATAG
CCCTGGCGTTCAAGCAGTTCACTTTCTCTGGTGTCACTATTGATCCAGTACATGCCGTAATGGTTACGACTACAGTAGTTGTTGTACTCTTCAGTACT
CTGGTTTTTGGCTTCTTGACAAAGCCACTCATTCACCATCTGCTTCCGCATAATGACAGTAGGAGAGGTATAGACAGAGAATCATCTATTTCAAAAG
AGACACTTCCTTTGCTTTCGTTTGATGAATCTGCCACAACCAATCTACTACGCGCGAAGGATAGCTTGTCCATGCTGCTTCAAAGACCTGTATATACA
ATTCATTCCTACTGGAGGAGGTTCGATGATACTTACATGAGACCGGTATTTTGTTCACCAACCATAAACGAGGAGTCTGTATAG
>PG0021928
ATGGTTAATCCAAATTTTTTGAAAGCCCCTGAACTTGCATCGTATAATGTTCGAGGGAATCAACCAGCTTTAGGTGTTGATAA
ATTATGTTATTTAACCGATACCTTTTCAGACAATGGCATATGGGAAGAAATCAACCCTTTGGAATCCAAACTCCCAACTTTTA
TATTTCAACTTGCTATTGTTCTTTTTGTCACCCGTCTTTCTCTCCTTCTCCTCAAACCTCTCCGCCAACCTCATTTCGCTGCTGAATTGTTGGCAGGT
ATACTGGTGGGATCAATTGCAATTGCAAGTGAATTTGCGCGAAAACATATTCCAACATTGGGTTCTGTATTTGCAGCTTCTCGAATTATGATGATGG
AGACGGTGGGAAACCTTGGACTAATTTATCATGTATTTCTTATTGGATTAGAAATTGACTTAAGAGCATTAACAAGTGTTGGACCTAAAGCCCTTGG
CATTGGTATTGCTGGAGCTCTTTTCCCCTTCTTTTTGGAGCTTCCTTGTACACTTTTATCGCAGAGTATCCAGAGAATTTCAGGTGGGGTTGTCTTTA
CTGGGGTGTAGCACTCTCAGTCACTGGCCTTCCTGTTATAGCAGAAATATTTGCAAAATTAAAGCTTCTCCATTCAGAAATTGGAAGAATAGCCATG
TCTTCCGCACTTGTTAATGATTTTTCCTCATGGATTCTCCTTGTATTTTCATTAGCAATAACTAGTAGCAGCTCAAGTACGTACTTGTCATTGTTTAGT
GTTCTATTCTTCATCATTTTCAGTATTTTTGTAATTCGTCCTGCACTTTTATGGATGCTTAGGAAGAGTACTAGAGAAGGAGAAGACTATAGTGAAGC
AACAATTTGTGCTATACTCACAATGGTCTTGGCTTGTGGGTTGATCACTGATATATGTGGAGTGAGCTCTGTTCTCGGTGCATTTGTATTTGGTCTTA
TCATACCATCAGATCTATTAGGTCATAGGTTTGTGTTGACAGTTCAAGGTTTTGTATCTGACTTGTTACTGCCTCTTTATTATGCTAGTTTGGGAATGA

GGACTCATCTAGGGGGAATTAACAGAGAGGACATGCTTATGATGGTTCTAATTAGCTTACTGTCTTTCATACCCAAGATTGTTTGCACTCTAGCTAT
GTCTTACTTCTATAGAATGTCGTTGCATGAAGGATTTACTCTTGGAATACTAATGAACACCAAAGGACTGCTAGCTGTCATGGCAATGAGCTTGGGC
CGTGACCATACTGTCATGAATGAAGATGGATTTGCCATCATGTTGTTTACTATTTTCTTCATGACCATAGTAACATCACCAATTGTTAACTTCCTCTA
TAGACGTACTAAGAAGTTCTTACCAAGCCAACATAGGGTGTTACAGAACCTAAAACCAGATTCTGAGCTCCGAATCCTAACTAGTATTCATGAAGT
ACAAACTGCTGCTGGAATTACTGCACTTCTGGAAATCTCGCATGCCTCTAGAAGATCTCCAATTTGCGTATTTGCTCTTCAGCTCATGCAACTGAAA
AAACATACTACCGCTTTGCTCATTGTTCATGGTGCTAGTGGCACTAGCTCAGAAAGTTACAGTAGAGCAGATGGACAAATTGATCAATTAGTTACGA
CATTCAATAACTTAGAGCATCAAAATCCGATGATATCTGTTCAGTTGTTGACTGCTATATCTCCTTATAGCACAATGCATGAAGATATATGCTGTCTT
GCTGAGGAAAAACAGGTGACACTAATCATTCTCCCGTTCCACAAGAGACAAACTATACATAATAACATGGAAGAAATGAATCCTGCTTACAAAGAG
GTAAATAATAATACTTTAGAAAATGCACCTTGTGCTGTTGGTATTCTTGTGGATCGTGGTTTTGGCACATTGTTAACTAGTAAAATAGATGATGATA
AAACTAGGAGTTGCAGAATAGCCATGATCTTTATAGGGGGACGTGATGACAGAGAGGCGTTGTCCTATGCTTTGCGTATGGCGAGACATCCAAAGG
TATCTCTAATAGTTTTGAGGTTTATTCTGGATGAAACTTCAGCACAAAATGGTGATGCAGATTTATCTCTCACAATGGAAGGTGAACATGAAGGTGA
AGCTGAAAAGAAAGCTGATGACATTTTTGTAAATGAGTTTAGGCAAAAGGTACAAAATGATGACTCAGTTGTTTATACAGAGGAGATGTCTAGTAA
TGGGGCAGAAACTGTGAAAACAATAAGATCACTAGGCCAAGATTTTGATCTCTATGTGGTAGGAAGAGGATTGGGCTTCTTTTCGCCGCTCAAAGG
TGGATTAGATGAGTGGAACGACTGCCCGGAGCTCGGATCCATTGGGGACTTGTTGCTCACATCAGACTTTTCTTCCACAGCTTCAATTTTAGTGGTG
CAACACCATGCTGAGCTCAATCCTGGATCATCAACTTAA

>PG0012169
MASNGTMKCPSPMKATSNGVFQGDNPLDYALPLAIVQICLVLVLTRLLAYILRPLRQPRVIAEIVGGVLLGPSALGRNQKYLHAIFPPKSLTVLDTL
ANFGLLFFLFLVGLELDPKSLRRTGKKALSIALAGISVPFALGIGTSFVLRETVSKGVNQGPFLIFMGVALSITAFPVLARILAELKLLTTDVGQMAM
SAAAVNDVAAWILLALAIALSGVGRSPVISLWVLLCGTGFVLLCIFIAPRIFKWMARRCSEGEPVDEKYVCATLAVVLAASFVTDMIGIHALFGAFV
LGVLVPKEGPFAGALVEKVEDLVSGLFLPLYFVSSGLKTNVATIQGAQSWGLLVLVIVTACFGKIVGTIVVSLLCKLPIQEAVTLGFLMNTKGLVEL
IVLNIGKDRGVLNDQTFAIMVLMALFTTFITTPIVISVYKPAKLAVTAYKHRTIQRKNTSKQLRILACFHSTRNIPAMLNLIEVSRGIEKREGLRVYA
MHLMELSERSSAILMVHKAKKNGLPFWNTEQVQDSNQIVVAFDTFSNLSKVSIRPTTAISPMNSMHEDIVASAERKRVAMIILPFHKHPRLDGHLET
TRGELRHVNRRVLQHAPCSVGILVDRGLGGASHVSSSNVDFSVTALFFGGHDDREALAYGVRIAEHPGISLIVVRFIVDPEVSGTSVKVEMNDKTN
PEAQSDDEEFLADVKQKSSIDGSIKFEERLVKDARGTIEAIREYNRCNLFLVGRMPEGQVVVALDKKSDCPELGSLGNLLTSPEFSTTASVLVVQQY
RSQLPEESLSSLKEGESSDGDCDSE*
>PG0012168
MATPASPLTCPKPMKATSNGVFQGDDPLDYALPLAIVQICLVLVLTRVLAYLLRPLRQPRVVAEIIGGILLGPSALGRSEKYLHTIFPPKSLTVLDTL
ANFGLLFFLFLVGLELDPKSLRRTGKKALCIAIAGISVPFVLGIGTSFALRATISQGVNQGPFLVFMGVALSITAFPVLARILAELKLLTTDVGRMAMS
AAAVNDVAAWILLALAIALSGNGSSPFISLWVLLSGAGFVLLCILIGPPIFTWMAKRCSDGEHVDEIYVCGTLAAVLAAGFVTDSIGIHALFGAFVL
GVLVPKEGPFAGALVEKVEDLVSGLFLPLYFVSSGLKTNVATIQGAQSWGLLALVIFTSCFGKIVGTIVVSLLCKMPVQEALTLGFLMNTKGLVELI
VLNIGKDRGVLNDQTFAIMVLMALFTTFITTPIVISIYRPAKLAVTKYKHRTIERKDTSKQVRILSCFYSTRNIPTLINLIEVSRGTAKKEGLRVYAMH
LMELSERSSAILMVHKVKRNGLPFWNKGEVSDSNQVVVAFETFEHLSKVSIRPTTAISPMNSMHEDIITSAENKRVAMIILPFHKHQRLDGHFETTR
TDLRHVNRKVLQQAPCSVGILVDRGLGGASHVPASNVNFTITILFFGGHDDREALAYGIRMAEHPGITLVVVRFAVDPALAGGSVKLKMSQNSSPE
VQPEDEVVISRLKESISTDGSIKYEERTVKDATELIE
ATKSYNKCNLFLVGRMPEGQVVASLNKNSECPELGPIGNLLTSSDISTTASLLVVQQYRSQLSQDALNSLEDGETSDGNESN*
>PG0009710
MAVKCASSPMHATSSGLLQGDNPLHYSLPLVIVQICLVLVLTRVLAYILRPLRQPRVVAEIIGGILLGPSALGRNKNYLNTIFPPNSLPVLDTLANLG
LLFFLFLVGTELDPRSLRRTGKKALCIAFAGITLPFVLGIGTSFALRSTIAQGVNQAPFLVFMGVALSITAFPVLARILAELKLLTTDVGRMAMSAAA
VNDVAAWILLALAIALSGSGSPIVSLWVLLSGTGFILLCIVIAPRIFNWMARQCPEGEPVNELYVCATLAIVLAAGFVTDAIGIHALFGAFVVGVLVP
KEGPFAGALVEKVEDLVTGLFLPLYFVSSGLKTNVATIQGAQSWGLLVLVITTSCFGKIVGTICVSLMCKLSVQESLALGFLMNTKGLVELIVLNIG
KDKGVLNDQIFAIMVLMALFTTFMTTPLVIATYKPAKMAVTEYKHRTIMRKDTTKQLRILTCFHGTRNIPTLINLIEATRGTEKKEGLRVYAMHLL
ELTERPSAILMVHKARKNGLPLWNKEKAGESNQVIVAFETFGQLSKVSIRPTTAISPMSSMHEDIIASAERKRVSMIIVPFHKHQRLDGHFETTRADL
RNVNRRVLEHAPCSVGIIIDRGLGGASHVSASEVNYTVLVLFFGGHDDREALAYGMRVAEHHGITLNVVRFIIDPAVIGASVHVDIAQNSGPVPESS
QEDDIYLSDQKQKSSGDSSIVFQESIVKDVRETIEVIR
GFKKCNLFIVGRMSEGQLVSAFDSKSHDCPELGRLGNLLISGEISTSASVLVVQQYRSELPQESLRSLRVGDSSRVGSSRIGNSARIRHSTKGDDGDE
EVTEI*
>PG0035252
MACPKPMKATSNGIFQGDNPLDYALPLAIVQICLVLVLTRVLAYILRPLRQPRVIAEIIGGILLGPSALGRNLKYLNAIFPPKSLTVLDTLANFGLLFF
LFLVGLELDPRSLRRTGKKALSIALAGISLPFGLGVGTSFILRGTIAKGVGQGPFLVFMGVALSITAFPVLARILAELKLLTTDVGRMAMSAAAVND
VAAWILLALAIALSGTGHSPLISLWVLLCGTGFVLLCIVLVPPIFNWMAKRCPEGEPVDELYICATLGAVLVAGFVTDAIGIHALFGAFVLGILVPKE

GPFSAALVEKVEDLVSGLFLPLYFVSSGLKTNVATIQGAQSWGLLGLVIFTSCFGKIVGTFLVSLLCRMPVQEAVTLGFLMNTKGLVELIVLNIGKD
RGVLNDQTFAIMVLMALFTERLRGKTRAKNSES*
>PG0005009
MDHQCPSPMKATSNGIFQGDNPLDFALPLAILQICLVLVVTRGLAFLLRPLRQPRVIAEVIGGILLGPSALGRNKGYLNAVFPPKSITVLDTLANIGLL
FFLFLAGLELDVKSLRQSGKKVLAIAVTGITLPFALGIGTSFILRGTINKGVNATAFLVFMGVALSITAFPVLARILAELKLLTTDVGRMAMSAAAIN
DVAAWILLALAIALSGDNLSPVVPLWVFLCGCGFVIGAYLIVPPIFKWISRRCHEGEPVDELYICATLAAVLAAGLVTDVIGIHAMFGAFVIGVLLPK
EGPFAGELVEKVEDLVSGLFLPLYFVSSGLKTNVATIQGIQSWGLLVLVIFTACFGKIVGTFIVSLLWKIPKNEALALGFLMNSKGLVELIVLNIGKD
RKVLNDQTFAIMVMMALFTTFITTPLVLAVYKPAKMLSKGDYKHRRIERKNPNTELRILTCFRSSRNIPSVINLLEASRGTERGERLSVYAMHLMEF
SERPSAILMVHKARHNGLPFWNKGQRSANHVVVAFEAFQQLSQVSVRPMTSISSLSDMHEDICITAEKKNIAMIILPYHKNLRLDGSFESTRPDFHL
VNRRVLEHASCSVGIFVDRGLGGTAQISASNVSFSIIVLYFGGHDDREALAYGTRMAEHPGVELTVIRFLVESDSSEEIVTIHTEGTSAATLVSADEG
FLAAFRTSISDDSSIKYEEKTVRNVSETITILRDYSRCSLFLVGRRPDGVLPLALSQRIDCPELGPVGSLLTSPEYTTTASVLVVQQYYDNLYANSSQQ
RDVASVPEN*
>PG0008849
MGFNVTNSIKTSSDGVWQGENTLHYAFPLLIIQTTLVVFLSRLLAFLLKPLRQPKVVAEILAGIMLGPSAFGRNKTFTNCIFPSWSTPILECVSNIGLL
FFLFLVGLELDLNTIHKSGKKAIGIAFAGISLPFLFSIGVAFVLRKIIKGIDSVGYGEFFLFIGVSLSITAFPVLARILAELRLLTTQIGEMAMAAAAFND
VAAWILLALAIALAGGGGVHHSPLISLWVFLSGIGFVVFMFLIIRPIMIWVAKKSSNGNNNIVEETCICLTLVGVMLFGFMTDFIGIHAIFGGFIFGLIIP
KNGDFSEKLILRIEDFVSGLLLPLYFASSGIKTNISQIHSVKAWGLVVLVVSTACVGKVLGTFVVGIMLCSMPMREALALGFLMNTKGLVELIVLNI
GKEKKVLDDETFAILVIMALFT
TFITTPIVMAIHKPSSTQNPQLEKPQKKIKKQNNLRILACLRGPRDARALINLIESLRSEKNNNNYASVTKLYVMRLVEFTDRLSSISMVQRARKNGF
PFIGRVLFRDDATDQVGAAFEAYSTLGKVIVRPTMAISGLSDLDEDIIHVAEKKRVELIILPFDKYWQMEGNEEVEIHAGHGWRMANERVMSQAW
CSVAVVVDRGLQLVDNGMRICIVFFGGADCSKALEIGSRMVEHPAIRVTLVRFIHHGSTNFDEVERTLDDSTIAEFKMKWGKQIVYSEKEANNLVN
EVLEIGKSGEFELMIIGNNKSKFPQGIMAKLFDEQQLNNSEFGPLANLLASSDKGIKSSVLVIQQQQQEAKFGNSKVASSFIDKDIV*
>PG0013814
MATDVVTVGNKTEENIVCYSPSMITTNGVWQGENPLDYALPLFILQLTLVVIFTRILVFILKPFRQPRVIAEILGGVILGPSVLGRSKRFTDTVFPLRS
VMVLETMANIGLLYFIFLVGVEMDIAVIRRTGKKAIPIALAGMVVPFLIGVSFSFMLHKSTQETKHGTFVLFLGVALSVTAFPVLARILAELKLINSEI
GRIAMSAALINDIFAWILLAFAIAFSENKTMALTSVWVLLSTAAFVVFCVIIIRPLIGWMIKRTPEGESISEFSICIILSGVMICGFITDAIGMHSIFGAFM
FGLVIPNGPLGLTLNEKLEDFVSGLLLPLFFVISGLKTEINAIDGVGSWAILALAIVLACVGKIAGTVLITLYYRIPIHEGITLGLLMNAKGLIEMIVINV
GKDQKVLDDKSFAIMVVAT
VLMTAIIIPFVTLIYKPARKFAPYKRRTVQSTKPDSEFRVLACIHTPRNVPTIINLLEASCPTKKSPICVYVLHLVELTGRSSAMLIVHNTRKSGRPALN
RTQAQSDHIINAFENFEQHVGSVSVQPLTAISPYSTMHEDICTVAEDKRVAFLIIPFHKQQTVDGGMEITNPNFRTINQNVLANAPCSVGILVDRGLS
GSTRLAANQVSHHVAVLFFGGPDDREALSYGLRMREHPGINLTVMRFLPGVSALEEARSGSRRSNMNDPNVLTIVTDDDKEKQLDEDYVSAFRLR
TANDDSVVYIERVVNHGEETVAAIRTIDQSHDLFIVGRGQGTISPLTAGLTDWSECPELGAIGDLLASSDNAAIVSVLVVQQYVGMGHGDHILTPDS
PGQQLHEHFNFGNTNNRTQIRGQQPQFHTQP*
>PG0030154
MADPEPLIDVGKLNEKVLCYAQTIYRFNGVWEGPDPLTPIIPLFFIQVSLAILITRFVTFVLKPTKQPPFVAEIISGILLGPTALGRIMRFRRLLFPNYNF
HVIETMAHVALVFYGFLVGLQMDMKSVLRIGIKARNVAIIGIIIPFVMGTILYFSLTRDEEVRGFIFYGGALTITGFSVLSKILDKQKILQTDIGKMAM
SSAVINDIGAWFILTLGYVVTGSTANIHWALICTIAYALFCVFYLRRAIGWTIRKMPEGQGYSEFFICSILAGMAISGVITDALGTHPIIGAFLFGLSIPN

QVLQAEIIDKLDDFVTGIFMPTFFVVCGLRTNFGQMGSIYEIVGYILLFVSAKILSSIAATFFSEMTIKEALAVGVLSNTKSIMALIIIEAGQAQQVLST
QLYSLMVAGILVMTAIVTP
MTMLHRPSQEIAPHKRRTIQKARMEEELRVLACIHGTHDIPSVINLLGSSHSTPASPITVFALQVVELVGRGSSMLEVHNSGKRGSRSLGHEETQTR
QIITAFDNYELRSDGVMVQVLTARSALSTMDEDMCNIAKDKRVAFIILPFHKQRGIEGEMEDVNPEIRAVNEGVLANAPCSVGILIDRGLSETSDYA
KNIVVLFFGGADDREALAYALRMVDRPDTRLTVVKFIPDEGASDIEQTEFADESHVNVQIDKESEKLMDDEFLNRFKISTANDKSVTYIELLLNDVE
EAVKAIKLMDQHNYDLYIVGKGRGVVSPLTAGLVDWCDCPELGAIGDLLVTSEFDSTFSVLVMQQYVKPIGDGSVNSYGSMSERIAGIGNMDMD
LDMQRADSESGDVFSSFRRRTEHMPRV*
>PG0021928
MVNPNFLKAPELASYNVRGNQPALGVDKLCYLTDTFSDNGIWEEINPLESKLPTFIFQLAIVLFVTRLSLLLLKPLRQPHFAAELLAGILVGSIAIASE
FARKHIPTLGSVFAASRIMMMETVGNLGLIYHVFLIGLEIDLRALTSVGPKALGIGIAGALFPFFFGASLYTFIAEYPENFRWGCLYWGVALSVTGLP
VIAEIFAKLKLLHSEIGRIAMSSALVNDFSSWILLVFSLAITSSSSSTYLSLFSVLFFIIFSIFVIRPALLWMLRKSTREGEDYSEATICAILTMVLACGLIT
DICGVSSVLGAFVFGLIIPSDLLGHRFVLTVQGFVSDLLLPLYYASLGMRTHLGGINREDMLMMVLISLLSFIPKIVCTLAMSYFYRMSLHEGFTLGI
LMNTKGLLAVMAMSLGRDHTVMNEDGFAIMLFTIFFMTIVTSPIVNFLYRRTKKFLPSQHRVLQNLKPDSELRILTSIHEVQTAAGITALLEISHASR
RSPICVFALQLMQLKKHTTALLIVHGASGTSSESYSRADGQIDQLVTTFNNLEHQNPMISVQLLTAISPYSTMHEDICCLAEEKQVTLIILPFHKRQTI
HNNMEEMNPAYKEVNNNTLENAPCAVGILVDRGFGTLLTSKIDDDKTRSCRIAMIFIGGRDDREALSYALRMARHPKVSLIVLRFILDETSAQNGD
ADLSLTMEGEHEGEAEKKADDIFVNEFRQKVQNDDSVVYTEEMSSNGAETVKTIRSLGQDFDLYVVGRGLGFFSPLKGGLDEWNDCPELGSIGDL
LLTSDFSSTASILVVQHHAELNPGSST*
>PG0023958
MAKSAYLTDNAFTERNIVCYDHSDVISNGYKVRNPLRFPAPLMVFQLSIISLTSLIIGVGLKPLGQPILVAQVLGGILFGPSALGRSEVLRETIFPPRGV
MALETAATFGVFFNLFAIGVECDSKRMFRPGKKAVIIGISVLFSSLISNMGMATLMQSFITMDPPLAKALPVVAISQCIVGFPNVCSLLKEMQMLNT
DQGRLATTSAMFCDVIGFTMGAVGFIKLQVEKEHSIPRKMGSGLSPLILVIFTIYFVRPAIKKTLKQRPDGKPVGENYFVCILIIVLTYIFAAETIGQHF
LFGALLLGMAIPEGPPLGAALINKLHYPIGKLLYPVFLTTSGLKTDVFTIHFKSLWVISLLVLFSVLIKIAVMIIITRFTGLTVHDSVIVGLMLNSKGIC
DVVFFNLWRISEALTDEHFAVVIIISVILVTVIITPLLKYLLGSIEEQSPTKRRTLQHSKPDTELRILVCVHHLQSVPTMVNLLEASNATEQSPIGVIGLV
LIELVGRAAPLLITHNHTQGEIPEDASISLQIINALRQYELAYETCVTLQPFTDITHFDLMHEDICRLSLDQNATFLILPFHKHWEIDGSIGTSSRAIQNI
NSKVIKKAPCSLGILVDRGILKGSMAILNNQKGYHVAVIYIGGPDDAESLAYGARLARHPNVSVTLIRFLMFGYDNARERKVDNSLIEAVRYENSM
NENFIYEERVTRDGVGLSASLRSLEDRYDLIVVGRYHEESPLLVGLGEWSECPELGVVGDFLASPDVGIIASVLVVQQQRVRGKLLNRAAKPVVVN
NQDGGPYPEMNNHNHNNNGMSTPTTVSNDHPRWEITIDRAN*
>PG0030375
MGRSGFGRIRAYSSLIFRPEGRMVLQTISDVGFMFHVFVLGVQVDPTMLRRAGRNAVLIGVSSFVMPFALGGLACYVLPHLTVIDDATAHFLPLLS
VINSASFFPVITSLLSDLKILNSEIGRIATLASLVNDGCIYAASILLTTIDASSSYSKWNGVMAIAWIGTFLIVIVFAVRPLVKHIARTIPERGAMKESHF
LMIAVLALLCGFVSQSIGQPPAVGTFILGVVVPEGPPLGSSMVYKIDSLCTGLLLPAKFAISGLTLDIFSLGRGKSLLGVEAVILLGYLGKFAGTLVSA
VHFAVSFQDAVPLALIMCCKGIIEASFYIGLKDTGAITSEAYALLLITMLVITGIVRPLIWYLYDPSRRYLGYRTNSIQHLDPTSELRVQVCIHNEDNV
PSLVNLLDVSNPSRRRPIAVFVLNLMELKGSAAALLVPTHNRKGKPKLKSLPSRTEHISNAFNILAHRNHGSMVVQHFTSIVPYATMHDDICTIAVD
KGVNIVIIPFHKQWAIDGTVGANFPAIRMVNQQVLHKAPCSVGILVDRGQLADNTQILFGHSLFRITMLYLGGPDDDEALAYCCRMLGHPHITMSL
VWLKHSSDNIEKSMESHMIQWFKANNVDAGRVSYKEEVVNDAVGTTQVLRSLEDSCDLCIVGRDHEQSELTLGINEWIECPELGFIGDMLATSDY
SFSLLVVQQTPPGTEFINIQPLQPVASSFYSGSGKYSQHSGSGKYSQHSGYGPFG*
>PG0007292

MGSHMMEPDDLATYAKIFRHGQNDSSICMSIGKIQSKGSILFHNTNPLDYSVPLLLSQLSLASLFILFTSTVFKPLGQPSNVIQIFGGLLLGPSFLGRID
GFIQLFYPYRSLVVIDAVALFGYMFFFFLIGVQIDPWILKRVEKKEFIIGVSTVATALVLSISTSFILITFHIHIDPLVAESLPVVATMSSVLGFPVIAHYL
TELRMVNSDFGRMALSCSLVSNMFGFLIIAITSLSSQPSVEKFMFLQSITSGIGFTMFVFLVVRPLVIWSTRRNPPGEPLKQSFICMVFIGVLLSGFCSK
ALGLNLFYGPLVYGLAIPAGPPLGSALVEKLQFIVSWLFMPIYFVKTGLVTDIFSVKLKNYLVLQSIILVACLGKFLGALISSIYNQVSLRDAISIGLVS
NVQGVLELGMFKMMKQNE
AIADEAFVVLCISLLIATAIVTPILKSLYDPHKRYAAHKNKNIQHMKPHSELRVLACIHDQENVPSTINLLEALHPSNQSHMDIAVLHLIEMVGRAHP
LLINHKLPLMMKHTNEASASKRIINAFKVFEKNFCETVTMHPFTGISPYVMMHDEVCTMALERRASLVMIPFHKRLTSSTSSSVNQKRASKMGIKT
MNDKILQTTPCSVAIIVDRSLVNTSRPILDAWSLYRVGVLFLGGPDDREALALGERMAGKQNISLTIVRLVLLHESGNYSNNNTSSSDYETIQKMM
DNEMLSEARSDMAGNYRVKYVEKLIRDGTGTAAVMRSMEDEYELIIVGRRHDSQSPLLLGLSDWVEESELGPVGDMFALADSQSNSTILVVQQH
NGG*
>PG0011649
MATAAATSVKLTGELPGAVNEKCYAEIPLHSPGIWGLPNPSLFLDFNLPLLLLQLAVIFILTQSLHLVLKRIRLPRLISEILAGIILGPTMLGKIPNFTEN
LFPQSGEIFIDLMSKIGYIFFIFLSGVKMDPKVVLRSGSRAWTVGLLAVILPVASFASLYFGFFSQDANMHRYRQPATQSIFLIQGLIAFPVVASLLVD
LKIMNSELGRLALASSLISDLFSNLGLTIFSTLRIGLLAEITTVISVQSFVLLLGIILLIVFTVRPISLWVIKRTPEGRPVNSVYITWGSVCVLLAVILVDN
AGLNYQYGPFILGLVIPDGPPLGSTLVDKLETLVSGMLAPLLITYCGMKVNLVDLYDLVFLNWVWVMVFFCLTVKYASVFLPALACKVPPKDAA
ALAFIMTSQGVIQMSFYLNNVINQTVDGETFSMLTASVLLIAALSHFCVGTLYDHTRIYAGYQKRDIQHASSNSELRLLSCAHRFDDVVGARKILDA
SFPCKESPLSVYALHLVELAGRASPVLIDHQLGQKNTSGVARSQKMVEVFIAFETQFLGSASTHFFTSMSLPRFMHQDICSLAFDKLASMIILPFHRK
WNQQGKIILDSSNLRTINNNVLDLAPCSVGILIDRQKIKRLASQSGNESSMYQVSVVFMGGNDDREALAYAKRMSRSPELQLTVVRFVSWDIDVRE
NQWDAVLDAEMLKEVRLLGQHQDNIVYREERVKDGAETALIIHAMEEAFDLIMVGRRHRDDLQQLLGLNEWNDLPELGPVGDMLAAAEINRPV
SVLVVQQQIVKNK*
>PG0014998
MSNSTEIISTQKVLQCVTFPPRGFSLGFFKKGSTPWIYSVPTIESQILIIYLLTQLFHFPLKRIGFPKIASEIFAGLILGSTFLGRYKSYQEKLFPLPSQSILG
ALTTFGFLLFLFLSGVKMDTSMTRKIGKRALVIGFLNHLAPLITGMITVFALSSDFYQEGVTPLSIPVEVISIAKTSFPVISYLLKDLGLLNSELGRLAL
SSALISDLVGLAIHAFIFLIVIGAKNTIQRAITDAILLIAFIIVVIFVFRPLMMWIVKRTPEGRPVKDLYILMIVLAVLLSGVFSAWFEQSVLFGPLIFGLA
VPEGPPLGSTLVDKLDPFTSGFLLPIFVTVMSLRTNLSAINPSASYTFANIILLCVGSITKILACLLPMLYCKMPLNDAAAISLIMSTRGVVDLASYSFL
RDDKIINQASFAFMVIATAVTSIFVQIMVKWLYDPSRKYAGYQRRNLMNSNNKLPILVCIHNPDNTAAILRLLEKSNPTRDFPIVSNVLHLIELRGRA
SSVFISHQVQTKAITDVAYSENVILAFQGFERNNYGAVTIQAFTAISPRNLMHEDICTLSLDVLASIIILPFHRKWAVDGSVEVEDHGLRTLNSSVLER
APCSVAILVDRGQLKRSTSVRASENAYCIAILFLGGNDDQEALAFAKRMAISGTISLTVIRLISKQDVSCDVDEVIDLDIVGDWKQSRSSWENVKYIE
HYVHETTETALLVRSLVDDYDLIITGRRNNTHSPLTAGLEEWTEIPELGVIGDMLASKDLKTRASVLVIQQQQTTL*
>PG0004171
MSKLNLTEDPYLLSKGLVICRGIHPPHTFGIFSGENPLEFSFSLVLLEISTIIAISRFIRYLLKPLRQPRIISELLGGIIIGPSVLSRSKGFRNFIFPDTADYAL
KNIGLIGFMYFLFISGVKTDLTQIKNVGKKQWYIAIFGVSIPMLCSLFIGLALQKSMEKELAKASSMLGVTSELAITAFPVIYPIIRELNLLSSEIGRMS
LSTALISDIIGIQFVVIFEAAKQGEHKSMAALWFLIYSFLIGASIFGGVRQIMIWIIKATPEGKSVEQIYVVFILLGVLLTGFLCDLGGIAVANGPLWLG
LAIPDGPPLGATLVEKTETIVMDILMPFSFAYVGMFTDISSIYTHWPHLQPIFFMALTAYLVKMVTVLFTSYFFNMPFRDCLALSLVLSLRGEVELLIF
IHWMDLKMITRPYFTMLVLMTIGVTSIVTPLISMVYDPTRPYMINTRRNIQHTSLNTELNIIACIHDEENVPGIINILFEVSNSTAPRTSMVHALHLME
LVGRAAPIFIDHQESVNIDQNPIHNALKHFGGENITINSYTSYSPKRSMYQDICKLALEKKASIIILPFYKGTQVLTRQGVQLVNSNVLNHAPCSVGIY

VDQGASPTSNNYNVGRRSSINKFALLFMGGSDAREALSYADRIAANPDVSLTAIRFLSHNGEGDNEMEKKLDDGLVTWFWVKNEGNEKVVYREV
VVKNGEDTIAALHTLQNEEFFDLWILGRNQGINPVLLQGLTHWSAQNELGVIGDFLVSMNSGTTTSILVMQQQILRGQEPTSLGFLKKIATCR*
>PG0018689
MAAATEQMAAAGSCNQELFNPIISMGWQYSLILVISHILQILLRPLGQASPIVQILAGFLMGPSGFSRIKAVEEFFIQSYNSGYYEFMALIFRTIIMFLIG
LETDFPYLMRNIRPASIIACGSSLGCTVFASAVTFLVFQETASHGSSFIMALMIIITLANAASPIVVRVAADLKFGTSETGKLAISSSLIADAYSVFLLFI
LSEFKSTSIAKWIFFFFLYFLIVGVVIVINMYLANWLNRRNRNKKYLGNTEIFILVAILYIAAMALEQLGFSSIIASFLIGSMFPRGGKAARTLLIKLTY
PIHNFIFPIYFGNHGFRANITKLKNLRNFMVFSILILSSIGGKIVGTLAACFHLKIPYREGVLLSFMMNLKGHVDILALTIGLANDLVSSQNFYDVMIAT
IIVNTLIWGPIVAFMVRRESDIIGYRQIYFESHNPETELRILACVHSPRPVATMLGLVAASRGPREVPITPYLMHLVELPGKKKTNLMYNQREDDELS
DEDDYGGNDVVEINDAMDMFTSETGLLVQQIKAVSPFSRMHADVCNTAEDIRASIVVLPFHKHQRIDGKLENGKQGIRTTNQKVLRHAPCSVAILI
DRGLTAGCLNPSGSDSLQHIAILFFGGPDDREALGFSKRLGMDHHVNLTIIRFLPSSSRGQISGVNIAHKTDDVLMAIPNDEVEKETDSAILADFHSR
YVATGQVGYVEKVVENGADTASALRDMAEMYSLFIVGKDGRGHSILTTGMSDWEECPELGKVGDFLASPEFDISGSVLVVQQYRPSKNDDDDDD
DK*
>PG0027255
MDFLKQNDTKVECRNQITIRIASMSIYLLGFFFAMFLCNCVHLLLRPISQPRIIAESIVGLLLSNLEFVRSRFLNDSEVQQTLNYIVDAIMVCHMFVVG
LEIDPNIFLQLTLPEAKVAYSGVLTTFVLACLITPLLNISKQSNAVFSSCLAIVLAGTDSPLLTRLITDLKIGKSDIGRFIVDAGIHSDVVSILLIAIGFLIF
DPDKNFQNRSVIMMLKMMAILVFQTLLASKVVPSVMKWVNNENPEGKPMKGSHLVVALAFIILICSMSPVVGYSKVLSAFLVGLFMPREGRISKM
MIGKVNYIFRTIFYPLFFFWVGTEAKLSEFEAGKIASWGKIIIPFIIATSGKVVGSVVSGLMLGFHWPESVATGLLLNIKGHFQVYLAINAYRMNVIS
MSTSIALVFVTFLTIIYTPVVVAKIIERARRRSPTQKMALQCVNPVNEIRILLCIRSPQDVYSAINFMEISRGPVNPGIMVYLTDMIDLTDKIAATLITQ
GEGIDAVTVTDPTVVEMREKITQDVNAYLNENCQGVSLRRMMALSTINNMHQDISILAEDLMVHLVILPFHKNQEEDGRLQVGHTGFRHINRKVL
RNAPCSVGILVDRGLGKTVIARSSISLNAAVIFIGGKDDREALVYAGRVARHPGVKLTVIRFLLEAAGDSVSSRISKAKANTSEHLEEMKIDDECFAE
FYDKHVAGGRVAYIEKYLINSGQTFSTLRSLEGQYGLFIVGRGGRVNSVLTVGMSDWEECPELGPIGDILSASDFSVTASVLIIQQHSLKGE
LDGLHDEFSIM*
>PG0031029
MERIVVRREKLRAWIFLFLSVVYSGRMTLAARSEKEIRARFYGNLVNSSAPVTNDGTIAKMFDRVLEKEFSENDQPEGSDGRSFNSTVADETGVLE
TVAKITHEKIKKNETQQTNDTRSFKLQDVFSLENEGSDDVTTLIDKKDNVFVMSNKKSKYPVLQVDVRLISDLVVVIVSAAIGGIIFSCLGQPVIVGY
LLAGSLIGPGGLKFISEMVQVETVAQFGVVFLLFALGLEFSLTKLKVVGPVAVLGGLLQIVILMFLCGTTAMLCGANLSEGVFVGCFLSMSSTAVV
VKFLVEKNSNNALHGQVTIGTLIFQDCAVGLLFALLPVLGGNSGLLHGIISMGKVLLILSMYLSVASILTWSFVPRFLKLMIRLSSQTNELYQLAVV
AFCLLSAWCSDKLGLSLELGSFVAGVMISTTDFAKHTLDQVEPIRNLFAALFLASIGMLIHVQFLWTHVDILLASVILVIVFKTTVATVITKVFGYNI
RTSLIVGLLLAQIGEFAFVLLSRASNLHIVQGKMYLLLLGTTALSLVTTPVLFKLIPAIMHLGVLMHWFPVENVAPDEEKVAMIVDTHNRVL*
>PG0029945
MTTRRRSSSRLQSLFVKFSTVSAITVLLLFLALAFAFALPESDQLLIGGSNGTRNGSEFSSGPRSRPKEDSFADMIDRALEKEFTENDKDEVNDAGSF
NNSVAEQQAVLETVARVKPKKNDTKKEEKSFQLHHVFKLDNDHGAEETPTLIDRKDNVFIISNFKSKYPVLQLDLRLISDLVVVIVSATCGGIAFAC
AGQPVITGYLLAGSVVGPGGFNVVSEMVQVETVAQFGVIFLLFALGLEFSTTKLRVVRAVAVLGGLLQVLLFICLCGITASLCGGKPSEGVFVGAF
LSMSSTAVVLKFLMEKNSTNALHGQVTIGTLILQDCAVGLLFALLPILGGTSNVLQGLISMTKSLVMLLSFLAILSILSRKCVPWFLKLMISLSSQTN
ELYQLASVAFCLLVAWCSDKLGLSLELGSFAAGVMISTTDLAQHTLEQVEPIRNFFAALFLASIGMLIHVHFLWNHVDILLASVILVVIVKTVVTSA
VVKAFGYNNKTSLLVGMSLAQIGEFAFVLLSRASNLHLVEGKLYLLLLGTTALSLVTTPLLFKLIPAVVHLGVLLRWFPPDSPSEFGFKSDNFRSDS
AKQRIALVSKDLIHEG*

>PG0022786
MELLVETRPFRVVEESISAESSGSDPTNAVIFVGISLLLGIGCRHLLRGTRVPYSVALLVLGIGLGALEYGTHHGLGRIGDGIRIWANIDPDLLLAVFL
PALLFESAFSMEIHQIKRCAVQMLLLAGPGVLISTFFLGAALKIAFPYNWSWSTSLLLGGLLSATDPVAVVALLKELGASKKLNTIIEGESLMNDGT
AIVVYQLLLRMVTGWTFNWGAVIKFLVQVSLGAVGFGIAFGIASVLWLGFIFNDTVIEISLTLAVSYVAYFTAQQGADVSGVLTVMTLGMFYSAV
AKTAFKGESHQSLHHFWEMVSYIANTLIFILSGVVIAEGILGGDNIFKIYDNSWGYLILLYALILVSRAVVVGVLYPFLRYFGYGLDLKEAFILVWG
GLRGAVALSLSLSVKRSSDGSQYISPDTGTLFVFLTGGVVFLTLIINGSTTQFALHYLGMDKLSAAKKRILNYTKYEMLNKALEAFGDLGDDEELGP
ADWPTVKRYITSLNDVEGEPVHPHTSSENDDNVDHMHLEDIRIRLLNGVQAAYWEMLNEGRIPQTIANLLMQSVEEAIDVVSHEPLCDWKGLKSY
VNIPNYYKFLQTSFVHRKLITYFTVERLESACYICAGFLRAHRTARQQLNEFIGESEIASLVIKESEEEGEDARKFLEEVRVSFPQVLRVVKTRQVTY
AVLNHLIDYVHNLEKIGILEEKEMTHLHDAVQTDLKRLVRNPPLVKFPKIRDLISVNPLLGALPPTVRETLIGSTKEIMKLRGATLYEEGSKATRVW
LISNGVVKWSSKSASNMHLLHPTFSHGSTLGLYEVLVGKPYICDIITDSVALCFSVDSERILTALRSDPAVEDFFWQESALVLAKVLLPQMFETTTM
QDMRTLVAERSTMSVYIRGESFELPHHSIGFLLEGFVKSHGSNEGLLSAPAPLLPLALEQQSFHNTEASVVHAASFSHQPSQYQVETRARVIMFDIA
GFLSGRGLQRRSSSLLSHSIDHPSRSFSRELGGLMSWPENTFKAMQHRQDVEQTGQQEMNMSTRAMQLNIFGSMISNTRRRPRSFPGISAAKTSHSQ
SYPEVRSDRAQTLVSVRSEGSTTLRKNAQVQGENKDMSIQLPSAPIEQSDTREYSSDDSGGEDEHLIRIDSGRPSFPQWITEEDKTS*
>PG1021988
MGLDAVARLGVSLLSDDDQVSVDSITLFVALLCGCIVIGHLLEESRWINDSITALVIGLCTGGIILLTTKGKSSHLLEFDEQLFFIYVLPPIIFNAGFQV
KKKQFFRNFVTIMLFGAVGTLISFSIISFGAKELLGKLDIGFLELRDYLAIGAIFSATDSVCTLQVLNQDETPRLYSLVFGEGVVNDATSVVLFNAIQK
LDLSHINSRAALVFTGNFLYLFLASTFLGVLVSSTLSCFMFYAHLPILSTFVL*
>PG0034953
MELFDYVNDNSSHEMVVSITVFVAILCLCLVIGHLLEENRWVNESITAIIVGLISGTVILLISKGKSSHILRFNEEVFFIYLLPPIIFNAGYQVKKKQFFH
NFLTIMSFGVIGVFISSSIIASGSWWIFPKLNFNGLTIRDYLGIGAIFSATDTVCTLQVLHQDETPLLYSLVFGEGVVNDATSVVLFNAVQKIDVARFN
GWSAFHVFLDFLYLFSTSTCLGVVVSLLTLLIPYYAGLITSYILKGLYFGRHSTVREISLMLLMAYLSYMLAELWSLSGILTVFFSGILMSHYAWHN
VTDSSRITTRHAFEAMSFIAETFIFLYVGMDALDIEKWKMSQQRISKTKQNKADSFISSLAGSVWTSMGIYITVLMLMAIGRAAFVFPLSVLSNFMN
RNATRTPPITFKHQIVIWWAGLMRGAVSIALAFKQFTFSGVTIDPVHAVMVTTTVVVVLFSTLVFGFLTKPLIHHLLPHNDSRRGIDRESSISKETLPL
LSFDESATTNLLRAKDSLSMLLQRPVYTIHSYWRRFDDTYMRPVFCSPTINEESV*
>PG0010663
MVFDFGTLVASLNRLSTSDHQSVVSINLFVALICACIIIGHLLEENRWMNESITALVIGLCTGVVILLISGGKNSHILVFSEDLFFIYLLPPIIFNAGFQV
KKKSFFRNFSTIMLFGAVGTLISFIIISFGATSIFKKWNIGNLEIGDYLAIGAIFSATDSVCTLQVLSQDETPLLYSLVFGEGVVNDATSVVLFNAVQNF
DLSHINTSKALELVGNFLYLFASSTILGVATGLLSAYIIKKLYFGRHSTDREVAIVILMAYLSYMLAELFYLSAILTVFFSGIVMSHYTWHNVTESSR
VTTKHAFATLSFIAEIFIFLYVGMDALDMEKWRFVSDSPQLSLQVSSILLGLVLVGRAAFVFPLSFLSNLMKKSPEERISFNQQIIIWWAGLMRGAVS
MALAYNQFTRGGHTQLRANAIMITSTITVVLFSTGVFGLMTKPLIRLLQPSPKHLSRMISSEPTTPKSFIVPLLESTQDSEADLGPNVPRPHSLRMLLS
TPSHTVHRYWRKFDNAFMRPVFGGRGFVPFVPGSPTEPSGH*
>PG0022490
MASVLASLFPKLGSLGTSDHASVVSINLFVALLCACIIIGHLLEENRWINESITALIIGLGTGVVILLVSGGKSSHLLVFSEDLFFIYVLPPIIFNAGFQV
KKKQFFVNFITIMMFGAIGTLVSCAIISLGAIQTFKKLDIEFLDIGDYLAIGAIFAATDSVCTLQVLHQDETPLLYSLVFGEGVVNDATSVVLFNAIQN
FDLTSVNLSIALSFLGNFFYLFLASTLLGAGTGLLSAYIIKKLYFGRHSTDREVALMMLMAYLSYMLAELFYLSGILTVFFCGIVMSHYTWHNVTES
SRVTTRHAFATLSFLAETFLFLYVGMDALDIEKWKFVGDRPGLSISVSSILMGLILLGRAAFVFPLSFFSNLMKKSSEQKITFRQQVIIWWAGLMRG

AVSMALAYNKFTRGGHTQLQDNAIMITSTITIVLFSTMVFGLMTKPLISLLLPPQRQLSTVSSGANTPKSLTAPLLGSREDSEVDLNVPDLPHPPSLR
MLLTAPSHKVHRYWRKFDDAFMRPMFGGRGFAPPAPGSPTEQGP*
>PG2021988
MQIGLLSAYLIKKIYLGRHSTDREVALMILMAYLSYVMAELFDLSGILTVFICGIVMSHYTWHNVTVNSKVTTRHAFATLSFIAEIFIFLYVGMDAL
DIEKWRFVKDSPGKSVGVSAALLGLVLVGRACFVFPLSLLSNFLKRSEHDKFGLKQQVTIWWAGLMRGSVSMALAYNQFTRFGHTQQPGNAVMI
TSTITIVLFSTVVFGLITKPLVRFLLPSSQGFNNLISSEQSFARPLLTNGQELEVEMGNVDPFRPSSLSILLKEPSHTIHNHWRRFDDAFMRPLFGGRGF
VPDAPELSKGGCDQY*

>PG00110663

GATGGTGTATTTAAGTTCGTTTAATCAAATATTTTTTAAAAACTATAAATAATTCAAAAATAAAATTAATAATTCACGTTAAATTTAGATGTATTTT
CGATATTTCTATCGTATGTTAAATGTTTTTCACTTGTAGTAAATAAAACAAAAAGAAGAAACGGCTAAAAGTGAAAAGTTGCATTAATAACCATAGC
CTAAGATTCCAAAAGACACAATCATCGCCATGGTGGGTCCCATTCACCCTCTTCTTCTTTTCCATTTAAATAATAGCCGTTTTCCCTTTATTTTAGTAA
CTCTAATCATTTTTATATTGTTTTTTATTTTTACTTACTATATAGTTAATTAAATGATTAAGTACAATTTACTAACTAAACTGTCTTTCAACAAACTCTT
CTTTTTTTAATTACCTTTCAAACGTAGATAGAAATAAAAAAAATCCAAAACTATGCACTAGAAATTAAAATCATTAATTAATTCGTTAAATTTGTGA
CCAAAATGAGAAATCACTCAACTCAATTTGTTTCACCAACTTTGCTTTTTAATAATCTCTTTCCTACTTTTTTTTCCAACTATTTAATTATAACTTTCCA
TATTTCAGATAACAAAAATAAAGAATATTTTTTATGGTATATTAATTATATCTTTACTTAATATTCTTTTATTTTCTTATATTTTATGTCAAATAAATTG
AAACAGATGGACTACTTACCTTTGAACACAGTAGAAACACTAATTAAAATTAATGTGTTGATTAAAAGGTCAGATTAAGTGACCAAATAGAGACAA
ACGGTCAAAAATGCAGACAGGTAGGATCCTTTTGATGAAATGAACGGCGCTGATGTGAATAGAAAGATGACCATTCCAATTTCCACACAAACTATA
AATTACCCTTTGATTCCATTTGTCTTTTCATCGATCTTCTTCTTCTTCTTCTTTCTCATCTCTCTTAATCAATCATTTCTCTAGAACTTTCCTCTGTCACT
TATCACAATTGATTCCGAATTTGATTTTCAAAAGCGCCAGAATTCTTTACTGAATTCCGCTTTGTTCAAGTTTTGCTGATTTGTGTTCAGTTTCGAGTT
TTCTCAACGGTTGAATCTGTGAGTTGTTTTGTTAATCTTGAGATCTGATTGAATTTTCGGTGGTTATAAACTCAATTTGACGGGCAATTTTGATCGGA
GTATTGGTAAAGTTTTCGTTATCTTCATTTAATTTTTTATTTTTTTTTGTATTTATTTATGATATGATATAAATGCTAATTGAATGAAATAAACAAAGA
ATATCGATGAGTTTAGTTCATGTGGCTAGTTCTGATTTTGGATTTTGAGTATTTTACAAGTGTCACGTAGTTGTTTGTAGCTGCATTTATACGCTTATA
TATTTGTGTGTGGATTTTTTTGAAAAGTTTATGATCTGAGAATTGTGTGTTATTGGTTTGTGGTTGTAGGGTTTGCTTGAAATTGGAATTAAGGTATCC
AAAGAGGAAACTTGTTTGAAAA

>PG0022490

AAAGGGTAGTTTGATTATAAAAAAAATAATGTGCGTATTAATTATGCGTATCATTGATATTATTTTTTAATTTATGAATAATTAATATTTATACCAGT
TATACATCTTTTTTGATATTAAGATATGCACGATTAATATATAAAAATTTATGATATTAATAATATAAAATACTTTAATGCATATATATTAGTATGATT
AAAGATATAATTGCTTCTCAAAGTGAGGGTACCTTATTTTGGAATTTATCACTGCATACTAATTCTAATACACTAATATATAAAAAATAATCTTTACA
TAACTAATACCAACATTATTATATTCAATATTATTTTTTTCACAAGCTCCACCAAGTGTGATCCTTTAGATATAAACATATTTTACGACCGATCTTAA
AATTATGTATTTAAGTCATGAGAAAACAAAAATACAATAAAATTATATTATACAGAGAGTAAAAAGAAAAAGGAAAAGGAAGTGACGTTATTCCC
GGAGTTTTCTATTTCTGTTTCTAGAAATTTTGCGCTTCTCCTCAAAATCATTTTCTCTCTCTGCTCCTGCTTTTTTTCTCCATTTTTGGGAGCAATTTCA
CGACGAAAACTCGAAGCAGACAAATATTTGAACTTTATTTTTGCACTTCTCTCTTCCATTTTTCTTTTTGTGGTTTCAGAAAGTTTAGAGGATTTGTAA
TATTTTTTGTGCTCTGTTTTCCGGCGCCTTTCAGAATGCAATTGTATTGGTGAGTATATCCAAAAAAAAAAAGCTGAGTATTCAAATTCTATTTTTCCGT
TTTTTGATGAATTTTCTCGGAAATGTTTGTGAAACCCTTTAATTTCATGGAAAATGATACGTACTTAACTTTTTTCGCTCATTGTAGGGCAGGAAATT
CCATATTTGAAGATTATTTGGTTAGAATTTTAAATGATCATACCCATGAAAAAATTGGAAACAAACAGTAGAAAACTATTATTTTTCTTTTTGGGTG
AAAATCTTGATTACGTAGAAAAGAATTGATTTTTATTTTAACTTTATATTTGTTTGTTTAGAAAATCAGACAAAATTTGGAAATTGAACTGTGGAGA
GTTGTCATAAACATGAATGTTTTGTTCTTTGTTAAGTTGGTTTCTAACGGAACTATTTTTATTTTTTTTTCCTTTTCTAATTATAGGTGAAAGGCACTG
GAAGCTTTTTTGTTTGCATTGGTGAGAATCTTTATGTTGTAGAAATGATCTTGTTTTCTGTTTGGGTGTACAGTAGATTGGAAAAACTGGGAGATTAG
AAATGGAGAATTTTAAATTACATTCGTGAAAACCATTCTCTTCTGCTCCTTTTGTTCAAATTGTTTTGCTTGGGTTCTTAGGTTTCGATTTATGTGGGA
TATTTTATTTCATGGAATATAGTATTATGCAGAAGATTGACATGACGGGAGCTAATCGCGATGAAAACCTGGCGAAGTTGAAAACAAGTTGGTGAA
CTGTGGTTGTAATACAAAGTGTCAAAAGAA

>PG0022786

CACCCGATAACATTTTTGAAGAGTCCGAGCAACATAGCATAAATACTAACTTGTGTTTGTTTAAAGTTGCCCCTTTTTTTAATTTCCAAAACAGCTAA
AACACTTTCAGCAAAAATTGAAAAACAAACACCATTCTTTCACAAGAATCAACAATTAGATAATCAAGAATCAAACCCCCACCCATCACATAACAC
TGCAAAATCTACTAATTAGTGTTTATTTAAAGTTGACCTTTTTTTCATTTCAATCACAGGCTCAAAAGCCACAGCTAAAAAAACACTTGAGAGGCAA
AAATTGAACAAATAAAAAACCATTCTTTCACATGAATCAACAATTGGGAAATCAAGAATCGAAATACCCAATAAAGAAAAGCTGCTATATCATCAA
ATTATCAAGAACCCAGGTAAGAAATAGTATCAAATTACTAGGGAAAGAATAAAAATGCTTACTTGAAGTGGTTAATTAAGAAAAATTAGTAGCTAA

TGACAACTTTTTTTAGCTTTGAGAAACGTGAACGGAGCTATCATAGACTGCGTTAAGGAGTGGCAAAATAGAATACAACACAGAGGAAGCAAACAA
AGGCGGTCCTGCCACCGCTGCTCACAGAGTGATAAATATTTTATTTTATTTAATTTGATATGGAATGTAATATATTTTTTATTTTTTTAAAATTAACTA
AAAATTGAAAATAATCATATAAATTGAAATAGATAGCATAAGAGATTTCGAATTGAAGTAATACGTTAGAAAATGTCTTTTTTTTTTCTACGGGTGT
TATAAATATTGTATGTCCTCCTAAATAAAATTTAATTAACATTATTCCCTTTTTATTTTATTACTTACTACAAAACATAACTTTATTTTCGTGAGAAAA
ATGAATTTTATTGACTTTGTAAATTCTGAAAAAAAATATGTTATTATCCCTACAACAACAAAGTGAAAAACTTAAATTCCTTAAAGAAAAAAGATTAC
TTTATCAAATTTGTGCTTAATAAATATTCCAAAAGCATTTTTGAGGGGAAAAAGAAGAACAAGCAACACAATCTCACTTTATTCGGATCCTAACTAT
ACGTGATCATATCAATTTAGAAAATTACTTCTTATATTATTATTTTTTCACTAAATTTATATATTTATTTTCTTTAGATACATATATCTCAAATATATAT
TTACACGAAATTAATTATAATTTATTTCAAAAATACTATACACAAACATGATTTGTATGTATCTAAAATACACTCTATTATTCGACTTTTTGCTATCTC
GCTCGTCTCTCTCGTCATAATCATAGTGATTACTTTACTTACTTAAAAAAATACAAAAATTGAAGGAATCATAGTACACTAGACAACCAAAAATGAT
AGCATCTTGTACAGTGCGTTTAATTTGAACTTGATGGTATTGTTGCATTCATAGTCAACCTTGTCCCTCTTTCAAAACACACTAAAATTCATTGAATT
TGCCACTGATATTCGATTATTGTTTGTGAAGCAAATA

>PG0009710

AAATGTTAGTATAAAAAAATTCAAGTGTAACACTTGCTATATGTATCTTTATTGTCTTTCTAAGCAATTGACTTATTGTTATTGGGGTCGGAGACTCG
GAATGTGTGCTTTCTCAAATCGCATGATATTTTTGGATATCAAAATTCAAATGCTTTGTCTGTGACTCAAACTCACAATATTAAGGTTGAAAGTGAA
GAATGTACTTTTACTTGTCACTTTGTATCAAGCTGAAAAGAAATAAACAAAATCATATTTTATCTTTAACAATAAGAACTTGTTCCGTAAATCATTTT
TGAAGATCTAATACTTAAAAATCAATTAATACAGATATTGTAATAAAATACTCATATTAATAATTTTTAAAAAACATGTAAAATTTAAAATGAATGA
ATAAAAGTGAAGTGAAACAGTAATTTTTATATCAGGAAACTGAAGTTTTTCCCCGTGTAAGATGTGTAAATGATTTTTAACGGTATTTGGTGTTTAA
ATGACTAATATGATAAGATGAGTTTGTTACTACATCCATTACTCTAAAAGTAAAATGTATATTTTTATTATGTAATATAATAATTTTTTCATTTTAAAT
TGTCTTGTTCTAAAAATAAATGTATACTTTTTACTTATGTTATACTAATAAATTGTGATTTTATTTATTATTCCTGCTAAGTAAATACAAGACAATTTA
AAATGAAACTGAGGGAGGGAGTATCTAATTTGTACACATTTCAACTCATTATGGCCAAATAATTAATTGCTACACGACGTGAATACCAGAAGACAG
ACTCGCAATTTCATTGCTCCAAAGTTGGAGGAATTAAAAATAATTAAAGAAAACAAATCACTTTAGAAGGAAAAATACATATCAAAGTTGATGTTT
GAAATTACTTGCTCGGTCCAATAATAACTATTCACTATTAACTTGACACACAGTTTAAGAAACACTAGATAATAAGAGAAATGATATTATATCACCC
TTGAATATATTAAATTTAATGTTTGAAAAAATATATTAGATATTAAATATTTAATAGCAAGAATAGAATAAACATAAAAAAATAAATTATTGTTTAA
TTTTTCAAACTGAGCAAATATTATTAAATAACTATTTTTGATATATGAACAAATAAAAATAGACGGAGCAAGTACTAGAAGGAAAAAGGAAAACGA
GAGAAAGACCTTTTTTAATTGCAAAAGCCAAGTTCCCTTAATGTGATGATAATAATTATTAAAACATGGTTGACTTATTATATTTATTAAATTTATCC
ACCAAAAGGGTATAATTGGCATATCAAAACTTGGTGGCTCCATATAAATAGAGGAGTTGAATGCCCTATGTAGGACACTCAACTCTTTTAATTGCAA
AAGTCAAGTTCCCTTAGTTCCTTACTCTTTGGAGTACTAAGTTTTTCTTTTCTTTTTTCATCAAGAATTTATGAGATTCTAGCCGATAACCTATCTTATT
TTCAAGGTTATTAATCTCAGACTCCACTAATTATAATC

>PG0021928

CTTAATAAGTGTATCAATTTTAAAGTGGACAAGTAATTAGGGACAAAGGGAGTAGGAGACTATGCACTTTTTTTGCCGCTTCAACTTCTGTGGTATT
GTAATCAAATTCTAATAACTCTGAAGTATTTATTGTACTACCTCCGTCCACTTTTAATTATCATGTTGCACTTTTCGAAAGTCAATTTGATTAATTTTT
AAAGTTAAATTAGATTACATTAATTTGATATTTTAAACCAAAAAAATTAGATATTCAAAAACTATATGAAAAATACTATAAATTACAATTTTTTTG
CATATTGAAATATTAGTCAAAGTTCTTATCGTTTCACTCTATAAAAGGAAACCATGACAATTAAAAGTGGACAGAGAGAGTATTTTCTACCGACTCT
TTTACTTCTACGATATTGTAATCAAATTGGAGAAGCTCTAAAGTATCTTCTATCCCCAATGTATCGTAGTCAAATTCAAAAAACTTTACACTTTATTT
TGCCCAATTGTATTGCTAGCAACTTTAGAAAACACTCCAGTTGCTTCTTCAAATTTTGAAACTTTTAAAAGTCTGATTTCAAGAAATTTATTTTTAGA
ATGAGCCAAAGATCAACTTTGTCAATATAAGATAAATAATAATGCAAATCATTATCATCTTTTGTTTTAGAAAAAAGTGCTTATGAGATGCAACAAT
ATTGAGCATGCAACATAACTAACACCTAACAATGATGATATTAAGAAACTCATCATATGTAACATCCCTCCTCATCTGTATCGATATTGTACAAAAC
GACTTAACACTTTTATGTTTCTATTCATTCACTATGTAAATCAATTTCTTATAGTATCTAATCTTTTCATTAATTTTCATAATATTTGTAGGTATTATACG
AATATATATATATATATATAAAAGTGACAATATTATGATTAGTATGTTATTCTTACATATAATACATGTGTAGACTTTAAGTGTAAATACACTAGAAT
GTGCAATTTTTAATCCTAATTAAAAGAGTTCCTTTTACCACATACTTCAAGTTGTGTCCTATTTTTTATTTTTTCACTCTCAAAAGTCCTTTGTAATTCC
ATCCCCAAGCGAAATGGGGGTAAAAGCGAGAGAAAATGTAAAATACTAGAATTTGACTTTAAATTGTTTAGGGGTATATAAGCCAAAATTAAACTA
TTTAAATGATAATACTATGTCATTTTCCCATTAAGAGAGAGAAATTTTTGAGAGTTCTTTTTTCTTTCTCCCTCTAACATCCCCAAAATCATTTAGAG

AGTAAAACCCAATCAGAACGGTATACCATCCATTTGTACATTCTCTGAATACAATGAATAAAGAATTTTTTTCTCATTTCTCAACGCATTCTCTAAG
TTATTCCAAATTTTATCTTTTCTGTTTTATGCCCAGAAAAAAAAGTGGCCCAAAGGTGGTGGCAGGTTTAACTGTCGCCATAGGGGCCGAAGGCGAT
AGATCTAGGAAGGAAGAAGGAGGTGGTAGAT

>PG0018689

TATAGCAAACACAAAATTCATATTTGTATGCTATAACAAAGTTTGCATAATTGCATTCCATAGCAAACATAAATATGTATATTTCGCTATACATATA
CAAAAGAAAGCAGTTGTATAATCTGCTTTGGTATACATATACAAAAAGATCAATTGTATAAAGTGAGAGAGACGAGTGAGCGAGCGAGATCTGGG
AGAGGGGAGAGAGGGGAACAAAAATATATGTATATATACAATTTTTTATTTTTAATATAGAGCAACTTTTACATATAGCAAACACAAAATTCATATT
TGTATGCTATAGCAAAGTTTGCATAATTGCGCTCCATAGCAAATATAAATATGTATATTTCGCTATACATATACAAAAGAAAGCAGTTGTATAATCT
GTTTTGGTATACATATACAAAAAGATCAATTGTATAAAGTGAGAGAGGTGAGTGAGAGAGCGAGATCTGGGAGAGTGGTGAGCGAGATCTGGGAG
AGTGGCGAGTGAGATTTGGGAGAGGGGAACGAAAATATATGTATATATACAATTTCTCTCGCTTTATACAAACACAAACACACTTTATACACTTGC
GTTTGTATAAAAAAACGAGAGAGGCGAGGGAGAGAACGAGAGTGCGAGCGAGATTCACAAAGAGAGAAGTGAAATAGCAACAGTTTACTATGAGG
TACAATTAAATCAAGCTATATTTATAGCATTTAATTTGAATTAATAGTTTGCTATTATATACAATTTTCCCTTAAATAATTATATTTATATCATTTGGTA
TAAAATCCATGACTTATTAGAGTTTGATTTCATACTTTTATTTCGACTGTCTCAAACTCTCGGTTATTCTACAATATAACAATGTTGAACAAGATGCA
ATCTGTCGACTCATGTGCATGGTTCATTTGATTTGTCACTTTGTTTCTAAGTGAATTTCAATAACAATCGTTAACCGATAAGCCAATAACCAATAACT
CAATATCTTAATGATTTTATAACAGTTTGATATGTCTTTAAACTAATAACCAATAAGTCGAACCGATAAACTTCAAAACTAAACCGAACCAACTAAT
ACGCACTCCTAATGCCATCTGAAACCAAATTAAGAAACATATTTACATATTATACCAATTATCTAATACCCATAACTCATTTAACCCGACCCAAACA
TAAATCAATCCGTCCATTTAACACCCCGAGTCTAGTAAATACACAAACAAGCAAAGGGCAAAGCAAATACACGAGACAACATCCGCTAATAACGG
ATTCGAAAACAAAGAAGAAAAAAAAGAAAGTTAGCAAATCCACTGCCCTGTTTTCGCACTTTGTTTGCACAAATGGTTACAACAAAGAAGAAAGA
AGAAAAAACAGAGCAACAAATAAGAAGAAGAAGGTTTGTGAGGGGAGGAAGAAGAAATTTATCTCTTTTTCATTCCTCCTCTCTCATCCTTCTTC
ACAATGTTATTGCTATCACATTCTTGAAAAAAATTCAAACAAAATTCGATC

>PG0031029

TTGGTTTTGATTTTAGTCGGTTCTTTTGTTTTTTTTTTTCCAGATTTTTATAACTATATGGTGTTTGAAAAAGAGATCAAATATATTTTCACTTACTTGT
GTGATACTTAAGAAATGTTAAATGAATAATTTTTTTTGTAAAAACTGTAAAAATTCACATGAGTACATTATTTTTTTGAAATATCAACGTTCATTATG
TTAAATTAATAAGATTAAAGGATACAGTCAAACTGAATACAAAACGAAATATTTACAAAGTAAATTGTTAACTTCGAATTTGAAAAAACTATAAAC
AATATAATTGTAACTTCGGATTTAATACAAATAAATATTTACAAGCCCAAATTTGAAATAAAATATATAAACATTGAAAACTATAAACTAACTAAA
AATATATTAACAAAGTAGATTATAAATAATATTTTTTATTCTATAAATAAAATAAAATAAAATTTATATATATATTATGTCGGTTTGGTTTGGGTTCG
GTTTGACTTTTTTTCTTTTAATACCAAACCAAATCAAGAGTGGTCGGATTTTTTTTTCAACCGTCAAACCAACCAAACCAATCTAAACCACAAATCGG
ATATTTTTCTCGATTTGATTTGATTCGTCGGTTCAATTTGACTTGTACACCCCTAGAGGCTAGATACAATAATAGTAAGATAAAGTAATTTCCTATTA
AATCGCATGATACTGTAAAATTGAACAAATTTTTTTAATTGTAAAACTTAACGTTCTCTATATGTGAACGACGAATGATACATTTTATTTCAATTAAT
TAATCAACAAGCCTCGCTAGAACTCTATTTATGTATGGCATGGCTTACAGTTTTAATTATTAATTTTTTATTTTTTATTTTAAAAAAACTATTCATTTT
GCTTCTACAAGCAAGCCAGTAACTAAAACCCCACTAGTCCACTACCACCTTAGGTTGCAAAATAGACACTAATTTAGATGTGTATTAAGTGATGAGG
ATAAAATCAAAAATTTATTTGAGAATATTAAAAAAAAAAAAATTCTAAAAATGAGGTGCTCGTGTAATTTTTTTGTCCACCGTTAATGGATAGTAGTT
AAACTATCTAAAAGCTAATATTTCACCTATATACCAGCACTTTTTTTAGGAAACTGAAAAATTAAGAAAGCCTTTTTTCCCCTCATATATTAAGTCTT
CTTTTATTATATATATTGAATATTTTCTAAAGGAGTATTAACAATTATATAAGTCTTAAAATTAATATATTGAATTAATTTGACAAAAATTAAACCGA
ACAGATCCTATTACTTGAAACTTCATTAAATTCGTCAGGCTTAGATCCCGCCCTGCAAAAAAAAATACTGAACAGTCAAATGGAGACAATAACTTCTT
TTCAATTTTTGGCTTCGATCTAATAAAGCGGAAAGTTCAAACAAACACCTTTTGCGTCTCCATTTTCCCTTTCTTCTTCGTCTTCTCCAATCTCCTGCA
AATTCTCTCTGTATTTTCTGTTCAGCTCTGTAA

>PG0027255

ACTTTCAAAATTAAAAAAGCGCTTTAAAGTTAAGGGGTCGTTTGGTTGAGGGATAAAAATAAATAGTCCTGGAATAGGAAGTTAGTTAGTCTTGGG
ATAAAATTATGTTGTCTTGTTTGGTTGACATGTTTGGATAACTTATCCCACCAATAGTGATGGGATAAGTTATTCCAGAATAACTAATCCCAAGATA
ATTAATCCTGCAATAACTAGTTATCTAATAAAAGTGAATGATTCTAAGCAAGGGAGGTGTATGAGTGTTTTATCATTATATTCATATTAATTGATATA
ATATATAATATTAGGTCTTGAAAAATTTGGGGAATAAGTAATTAATGTTGAGAGTAAAACATGAAAAGAGAAAAAAAAAAGTTTTTTCTTTTCATATG
CTAAAAGTGAGAAGTAAAGTAACAAAATATTTTTGAGTGAACTGGGGAATATTTGACAGCTTTCTTCTACTTTTCAATGGTAAGATTGGAAATTTTG

TTGTAGCAATTGTGTGTTCAGTGTGTTCGGTTTGTTAACATGAAAAAGACAAAAAGTTGCTTGTGCGGCCGCTCATTTCCTTTTACTTTGAAGCGACT
TGTTTGCCTATAAAAATTATGATTCACGTGTTTTCAGAATAAAATTTTGCTTTATTTGAAAATCAGTGTTTGACCATGAAAATTTGAAATTTAATTCG
AAGTTGTATATCAAGTTCGAAAAACAACTTATAACTTATTTAAACTCATTTTAAAATAGTAGTACATTCAAAGATGAATATTATTCCACATATTGTA
AAAAGTATAACTAAACACAACTCAAACTTCATATAGTAAAATGAAAAATATTTGAAATAAATTAGGACCTTAAATGGGGAATACTAAAGCATAATT
AATTATTTTTCCTACATTTCATTATTATTGATGGAGTATTATTACATTTTATAACTAAGTAAAAAAATTGTACTTACGACTTAAATAAATTATAGATA
TAAAAAATTAATGTTATTGAACCCGTACACAACACAAGCTATCCATAACTAGTTTGATATATGTTGGGAGAGTTAGTGGATACTCAATATTACTCTT
GACTGATGGAGACTCGATATGTCCATCGCTATGAAAGAAAAAATATTATTTTTGAGTCATTGTGACATCCTCATAGACAAATGGAGTCAATTAAGTT
TACAATCTCCTCAAGCTACCTTTCTTATATTCAAAATGAAGGTTCAACTCCGAAAATAAAAAAAATATATCATTTGTTACATTTTATATGACGGTATT
GATTAGGCATAAGTTTATAAAAGAAGAAATATTTTTTATTACACAAACTAAACATGTAGTTATATAGTCAAATTTCTTTATAACACGTCGAATAATA
ATATTATTAGCTAGTAAGTAGGAGACATATGAGCAAGAGTAAAAGGATCCCCATTTCTTGAGGAATTAATTCCAAATAACAATGGATTCATTTCATT
CATTTTTCTCTTTCTGCTAATTGATCTGAAGAAGAAAAAAAA

>PG0011649

ATGTAGTAACTGGTAACACCTTTGGCAGTTCATTTTATGTACTCTACTCGATAGTGCCAGACTGCCAGTCCCACGCGACTAAACTAAAGAAAGGAAT
CCTTTCGTTTTCTGGATATTTCAGAATGTTTACTTTCTAAAGCTTGTTTTCATATTTCACTCATCCAAGTGAGATAGGAATTTCATACTTCGTTGGCTC
AATCAACCTAAACACACTTCACTATTCTTTTTTTTAATTGTTCATCGCAATACTTATGTCTTTTCTTTCGTCTCCCCACACCTTACAGGATGAATGGAG
GACAACACTTCTTTTGAGAGTGAAGGAAGCGTTGAAGGCGAAGGAGCTAGAGGAAGATGATCTTACTTAAATTATTCTTATATGAAACTGGTTTGT
AAATTGCATTATTTGTGATTCCAAATATTTCTACTTGGTGATTGTTTTCTTATCTCATGCTACTTAATTTTTAACATTTCATTTTGTACTGATTGAGTTT
GACAAGGTTGTAGCCTGGTAGCTAATATTAATGGTTTCACACAATATTAATATTAATTGATGGAGTTTGATTACCAAATTCAGAGGTAAAACAAATT
CAATATATTGTTACATGGTACCGGTTGCTGGAGCGAGGTAATATGTATCCCGTGAAATAATTGTATCTCAAGTTAATAATATTATATTCACTTACAA
ATCTCTAATTATTTTTTTTCAAGTGAATACATGTTCAAATATAACTAAAATTATACTTTTCTAGAAGTAATCAACCAATGAAGAAAAGATAATTTAAA
ATCAAATTGTAATTAGTTCACTAAAATATACTTGCGCCTTGAAGTAAATAATCTTAATTGTTTTTTCTAGAGTTTCGATTCATACATCAACCTTATAA
AACAAAAACAGGTTGATGCACAAAACATTTTAGTCATAGTGATCAGAAGAGGATGTTACTCCAAGAAAAAAATGCAAAATATATATTAATGACTGA
TTACATTATCATAATTCGTACATAAATATAATTTTACTATCAACCTTGTAACACACTTCATTTTCTTCTTCACTCATGTAACTCCCATCTCCTTATATCC
CAGAATATTAATCTAGATTATATAAAAAAGCTTCTAAAATATATCTAAACATAGAAAATAACTAAGAAATTCTATTATTTTCTTAAAAAATATTTATT
TTAAGTACTTTCTCTTATTTTTATTTTCATTTTATGTCCAATATTTATGTTATGATTCAACTAAATTCCAATGACTCGAACCTCAAACATGATATTAAC
TCCTCCTAAATTTATTTTCATCTTTCCCGCTACATTGCCTTCTGCAAAACAAAACCAAAGGCATTGAATATTTTACGGGTGTGATACTATTTTCGTCCC
TCCATTTTTGCTTCAAACAAAAAATGATACTACTACAAATCTCAAAACAAAAATTGTTGAATTTCTCTTCAAATGTGGGTGTGATTCGACCTAGCCA
AGGTCACCATTATTATTATCATCATCATCA

>PG0007292

GAGCCCCTCTCAAAGATTATTAGAATTGAAAGAGAGAGCAGCTTTGCTGGAGCATATCTGGTTCTATGTTATGTGGACTTTGAGGAAGTAGTATATT
CACTAGAACACATCCAACACAGATACTTCCAGTTTTAATTGGTAGTTTGAAGGATTTATATGCAACAGTGGAACTGCATTTTCGGAGGATAAAATAG
AGCTTTGCAACATAAATTTGGCTTGCATAGATATTCATCTTTCTTTTTTTTTGAGGAAATCTCAAAGAAAATGGAGGAGAACCATTCACCAACATGA
GGTTGTAATAGGGAATGTGGTCATGCATTATAAAGAGATGCAACAAAACTATAGTTAACATGTGTGGATTTGTTCTCTTTCTTGCGCGCGTGCAGAA
TGTCATCTGCTTATATTACATGCGTATTTCTCATCTTTTTGGTGGACTTGATGGACATTGTGGAATGAGGTTTAATTGAACGCTTCTTTTGCTGCTTTG
TAGGCTTCAGCAATTAGATTTGAGTTGCGTGCAGGAGAGTGGGATGAAGCTTATATGCAGATGGACGGTGAGCCATGGAAACAACCAATTAGCAAG
GAATATTCAACCTTCATTGAAATTAAGAGAGTACCATTTCAGTCTCTCATGGTTAACGGGAAAAGAAATTGATCGTTCTTCTCTTGTCATTTCCAGAT
CTTCTTAGAATTGTAGTTATCTATGTTCGTGTAAAGCATTTCGATCTCCCAGAATAGCTGTTGCTTGTTAACATGTACATACATTATGTGTATTATAAT
TTATACACATGGGCATATTATCTGATTTATGGGTAGATTATGCACAGAAATGAGTTGCATAAACATTTTAGTGTTGATTTTACCATGATATTCAAATG
ATTCTTTTTTCCCCAAACAACACATACTAATCATGAATTCATTTTTCATGATCCCAATGCCCAAAAAAAAACTGAAGTTTGTCTTGGTGAGTATTGGTC
ACTTGGTCATTTCTAGATCAGATTTCAGACCAATCAGAGTCCGTCAACTAAATTCAGAAAAATCAACATGTATTAACACACAAAAAATGTCCATAAT
CATTGCCTTGAAACACTGAGAATTTTTCACCAATCCGTTCTTTTAAAGCAGCACCTGCGCTAACACAAATTAAGCAACCATAATTTTTTGAACAGTG
CTTATGTAGTAAATTGTGATGTATAATAACTATTTTGAGAAGTCGCTTATTTTTACTAGTTGCCTTCTTTTTACTCGTGTGAAATGTATGATTGGAAAA
TTACTAGGATATTGTGAAAAGTTATTTTTAAAAAGCCGAATAATTAATTAATATAGCGAGAAAAAAGGAGATTGATTCCATATTGACCATCAACATG
ATTTCTTCTTTTCTCTATTCTCTAACTAAAATCTCTTCCTTTCCTCCTCGAATGAGGAAGAAGGGGAGGAAGTATATAATTTTTGAAAGAGAGAGGAA
TTAAGGTGGCGGGAGCGGGGGACGAGGGATGTTGAA

>PG0013814

GTCCAACCAAAATAATTAAATAAGAGACCTTATTTGTCAAATTATAACCCCCATATAAATGGTATGAGATACGGTCGGGACCCACAATTATGGACCT
TGAGGGGTGCTTAACACCTTCCTCTCGAGGTAACTTGAACCATTACCTGATCTATGGTTTCGCAGACCCTTAAGTGAAATTAGAATTAGTTAGGTTA
GATAGGTGTCCTAACGCGCCTTTAATTCGTTAGGTGATAACTCTTCTCTTAAAATAAACCTAAATGAGTTGCTAGGTCGTTGCACTAACCCGTTTGTG
AAAAATGGGGAACGACAATATATATATATATTAAGACCATAGAAAAAATAAAGTTTTGTTATTAAAACGAAAGAAATAACTTTAAATATCAACTATAA
GTAAACAAAAAAAAAAAAGTTATGCCTTCGATTTATTTTTTGTTTTAGACCACCAATTAGCTTGAGCCGTCGCTAACTCTTTTATGTTTGATATCAATC
CATGCTGAGTAAACATTAAAATGATCAGTATTTTTATAAACAGAGTTGAGTTTGACCTAATTGTAACCATCATCATGAGATACGGTCGGGACCCACA
TTTGTGGACCTTGAGGGGTGCTTAACACCTTCCTCTCGAGGTAACTTGAACCATTACCTGATCTATGGTTTCGCAGACCCTTAAGTGAAATTAGAATT
AGTTAGGTTAGATAGGTGTCCTAACGCGCCTTTAATTCGTTAGGTGATAACTCTTCTCTTAAAATAAACCTAAATGAGTTGCTAGGTCGTTGCACTAA
CCCGTTTGTGAAAAATGGGGAACGACAATATATATATATATTAAGACCATAGAAAAAATAAAGTTTTGTTATTAAAACGAAAGAAATAACTTTAAATA
TCAACTATAAGTAAACAAAAAAAAAAAAGTTATGCCTTCGATTTATTTTTTGTTTTAGACCACCAATTAGCTTGAGCCGTCGCTAACTCTTTTATGTTT
GATATCAATCCATGCTGAGTAAACATTAAAATGATCAGTATTTTTATAAACAGAGTTGAGTTTGACCTAATTGTAACCATCATCATTAGAAATTAAC
ATAATATAAACGTGAATTAATTTGAATGCAATGAATCATGCATTAACGAGACATAAACTTAAATGAATCATAATAATAAATTAAGAACAAGACATA
ATATAAAAGTGGAAAAAAAAATCGAAAAAAAAGGAAAAAAACCTTCAAAGCATATTAGAGATGCCACATTGCCTTTGTTATTCCAATGGTAATTATG
GACCATAGAAGATTTAGCGCTCAAATGAGTCAATCAACCTAATTTCCATCTTGAAAATTTTTCTGTTTGTTTTCTCCCTTTTTTCTTTCTTCTTTCATGG
AATTTTTTACTCATTCAAGAACGTAGCACGTACTCATGCATGTTCGTGATGATTTTATATGGTTGACAAGATATTATGTTTTATCATTTTTTTGGATCT
AATTTTTGAACAAGGACAAGGTAACATTGGAGAGGCAAA

>PG0023958

TATTCCCCATTCGTTGCACCCTCCCTCCCATTTCTTCATTTGATTTAGGGCTAAAAGGTGCAAATGTATAACAACAATTAATGGTTCATATTTTTAAG
ATTGCGTCTCTTTTTCTTTAATTTTGTGATATTTGTAGTTTAATTAGGGCTCAAGGTTAGTGAGATTAACATTTACATAGTAAAAATTGAAATTTTTTG
GCTCCTATTTTAGGATTATTGTGGATGAACTTTACATGATTTGAGGTGTTGTTGATATGAGTTCGATTTCTTATTTCTTTTTGGGTCTATTTGAGTTTGT
AGTGCGAAATTTAAATTGAAATTATTATAATAAAAGGAAGATTTAACCTCCATTGAAGTACTTTAAGCTTTGAGAATAACGGGAGAGATATTGGCT
ATAAAATGGGAAAGAAGAAGAGAAAATAAAATAAAATAGACTAAATAAGTCCCTCACGCGTCAATAGCAAATGTATAACACTCACTTTGTCATGTC
AGCTGAAAGTGTCAAAATGACACAACGGAGCATGCCATTAGGTGTCTAAAATGAACAAAGTCTAGTTAAAGTGTCTAAGTGAAAATTGCTGTCAAC
TTTAGGGGGCCACCGATGAGTTAGGCCATATATATATACACACACACATCTAAAACAAAGAATTGGTCAAGCTAAATATTTGGCATACACTCAAAC
CTCTATTTAACCATCATTCCTTAAAACAACATTCCACTGTAACGGCCTAATTTCTTTAGAACTAATTTTTGATGTTATGTTTTTCGTCTCTATAACA
TTCTACTTATAACAGTATTGACATTCACTATAGAAGTACACTTTTTGTAAAATTACATCTCTATTTGTTATTGGTGTCATGCGCATGTTAAATTTCAAG
TATTAATATTTAAGAGAAAAATTATACTTAAATGGTATCCATCATTTATAGTCATAACTCGAGAGGAAGGCTATTGGTGACCTTTTTTATATTGATAT
TATTTTTCTATTTTGTTGGCTAAGTTTGTTTACCAAATTGAGTAAAGTAAAAAAACAATACTACTAAAGAAATGCCACTTTCTCCACATATTTAAATT
TTGAATGTATTTTGAAATGTTTCAATGATATCTGGTCATTTTGGTCTCGAAAAACAATCAACTCTATGACAATTTGTATTAATAATTTTAAAGCACTT
GTCCAATGTTCTTTCAACTATAATTATTTACAAAAAAGCTTTAACTTAATACTAATCCCCTCTACCTTTCATTTTTTTGGTTCTAACTTAAACTAAAAA
AACAAAAGGATATTAAGTGAGGCAAGGGTCCTTAAATTGAGCAATTTAAGTTGGAGAGAAAATAGTAGAGTATACGTGTGTAGATAACTAATGAAT
TTATAGACCATTTACTTGAAAACAAACAAATATAATCCACCAAAATGATGAGTGATGATGGAATAAACAAAGAATTGATCATCATCTGTTAAGATA
GAAGAAAGTAGGAAGGAAAAAAAAAGAATTTAAGAGA

>PG0030375

TCCATAATTAGCTTTAGAATTCATAAATTTAAAATTTCAATATTTGTCATCTTACTATGTGGACTTCTTCCAAAGCGAAGGATATCAGCTTTAACAAT
CTCTATTTCCCACACTCAACTTGTAAAATCAAGTAATGGAGTTAGAAATTTGACGGAGGGTATACAAATTTTTTTATTGTAATAAGAATATGTAAAA
TCAAATATATATCTATAGTAGCGAATATTTAATCCGTTTGTATACACATAACTTTCTAGGGAGGGTGTGCACCCAACCACCCTTGGCTCTCCCCCATG
TCCTAAACAAGTTAAAAATAACAAGATTGAAACAATTTGTTTTAAGAAAATAAAAAGAAAGAGAGAAAAACAGGACCATTGAAAATTAGGAGTGT
GTTTAGTAGGAAAGTAATCCAAAAAAGTAGGCTAATATTACGGATATATGTTCAATTATCCACATTTCTATCAAGCAAATTCAACTTACTTTAATTTT
GTAACATTCTAAACTAATTGTTTCACCAAAATTTTCTTCCATTTTTTCTTGTTTAATCAACAAAAATGGTAAGAAGAAAAGTTTCAGCTTGGGCTGGA
TTTGTACAAGGGCGTCATCGTCAAGAATTAATTGTATGCCAATTTTCAGATATGACAAATTCAAAGGGATGTATTTGGTCTGGTGGTAACCCTTTTGC
TTTTAGTTTACCACTTGTGTTGGCCCAATTGATTTTCATTTTCTTTGTTACAAGAATTACTTTTGCCCTCATTCAACCTCTTAAACAAAGTATGGTCAG
TGCACAACTTATTGTAAGCTTCTTTTTTCTCACAATTTTATTTTACTTGTGTTGTAAAATTCAAGCTTACCAAGTAGTTTCAGGATTTGAATTTTATGA

GTTTGAACAACTAAATATAATACCGTTTTCCGTTCATTTCTAAACATGTCTCTTTACCCCTCTTGAATTGAAATTTTCCAACCTAACATGCTTAAGACC
ACAAGGATATTTTGGTACATTTTACATATCTTTAATTTAAGATGCACCATTCAAAAGTTTTAAACGGCATGCCAAATCAAAACTAGACAAACAAAT
TGAAAAAGAAGGATTATAATGTCTGGTTTTGTATGGAAAATGTGGGGTTTAACAGAAATTCTTGTGCCCGAAAATTCATGCTATCATCATTGTTACA
AGTGGATTCAGGATTTAAATTTGATGACTTCAAGAACCAAACCTGATACCATTTTAAAATGGGTTCTTAATGTTTTATTTCTTGAAGTTATACTAATT
TGTCACATGTGTATATTTATGTTTTGTATCGAAAATACTGGATTTAGTTGAACTTGTTGTTTATAGGCTGCTGCATAGATCCGCCTCTGATTATACCTT
ATAATTCTATTGCTTGCAGAGCTACGGTTTTAACTTGATGAGTTTAACCTTTTGTTTATACACCTCTGATCACATGTGTTGCAAAATTTATACTATCAT
TATTGTTGATCTAGAACAGGCTGGCATTATC

>PG1021988

AAGTCAAACTAGCTCACTCTGTGTGAAACGGAGGGAGTAATAACTTGACTAAATTCTTCTGCACTGACCAAATCTTTTGCTTTCCTACCTATTCACTT
TCACCATCAGCAGTTCAGCACTAGTCATGTCACAACTCATGTGAAATACACTCAAATCGATTATAAATAAAATTTAGCAAAATTTTAAATGGATCTA
GAATCAATTTAAAATGTTAAGTTCCATTTTTATCCGAATCCTCTATTTAAGGAAGCAAATAATGAATTTTGGACTTTCACAAGTGTAACAAAGTTGG
ACACATATTTTTGGTTACTTAAAAGAGTTATAAAAAGTATATAAAACATCCTTTTCCAACTTTGCTTCTAATTCTATTTTTTTCATTCGTACCTTCCTC
CTTAAGCAAGAAAAAAAATTAAAAAAATTCAGTCTTTGTTCTTTATTGTCTTCATACAATTAAAATTTTGCCTAAACTGCAGATTTTCAGCAAATTAAA
AGGTAAAAGTTTTTTTTACTTTTTTTCATTCATTTAGTTGTGATTTTGCCTCTGCATTTGCTCTGCAAATTGAAGGTAATTTTATGAGTATTTCTGTTCT
GTTATGAAAGGATTATTTATTTTTAGTCCATTTAGAAAAGAATGTATTTTTTAGGGGCAACTATTTTATTTCATCTTTTCGTATAGTATGTTTAAGATT
ACAAAATTAACAAATATTTTGGTGAATTCCAAGTATCTTTAGTTTAAGAACATATAAATATAATCAAGTTGAAACAAAGGAAGTATTTTAAAAGTAT
TCGTATATAATCTAGACAAGGTGGGAGTGGCGGTGGTGGAGATGGGCGAAGATTATGTGTGTTGGAGGGTGGGGAGGATATATAATATATTCAATG
TGTAATGTCATTTGTAGAAGTTGTTTTTTGCACCCATGGGTTAAAAAAAGAGGATTTTTTTTACTGTTAATTGTCATTTGTTGTTTCGTATTATGTCAT
GTGATTCGAGACAGAGTGAGGATTTGAAGTATATGAATTTTAAAGTCATGATTTGAAGTGTATGGGTTTTGAACTTGGAACTGAACCCATAGCTCGT
ATTAGTTAGTGGAATATTTATACATAGTTGTGGCAATGTCAGGAATGTATACTTGTTAGTATCTATTGTGTGTTTAGTCCACTTGGATGAAGGATTGT
AGTTGAATGAAACAGTAGGCGGAGTATCCGCTGGGGCCCTTTCCAGGCACTTACTGAACCTGCTGACACTTGTGAAAAGTGAAAATGCAAAAGCGG
CCTTCTTGTTCAAACTTAGTATAAATGTTTTGTTTTGGAAAAGTATGTATATATTTGTATCTTGGTTCAAGTTTTATGCACTGATGATGTAAAAATTGT
TTACACTATTAAGTCATTTAACTCTTGTTACTTGATTAAAGTTGTTGTTTTTTAATTGGAAATATGCATTCGAAACAGGCAGAGGGAACTGATTGTTG
TGTTTCTTGACCAGTTGTGGCTAGTTGCAATC

>PG2021988

NNNNNNNNNNNNNNNNNNNNNNNNNNNNNNNNNTTAGTATCTCATTAACACAAGGAAAGCTTCTATGCAGTAGTAAAAAAGTTTCAAAAGATATTT
GAAGTTATGAGTTTGACTTCCACCTGCACTTTCTTAGTATTTTCTTGGATCCCTTATCTCTCGTCCTTTCTCCACCTCTGCCACTGTGGTACCATAACC
CTGTGAGAGTATTTTCTTACTCTGGATTTGAGTCCGTAAGAACTATGTTGCTTGGACTCTTCAAAAAAATATCAACAGGTGCATGTTGGATCCTCCAAA
AGTATTGCCTTTTTGGAGGATTTGACACGGGTGTGACACTTTTGAAGAGTCCGAGCAACATAACCTAAGAATTAATCGCCATTTAATAATTTGATTT
AATGATATGGATAAAATTTAAGCTCAGTCTTTCTGCGGATGGCATTTTTCTGTAAATTGATTTTGACCTCTTATCTTCTGATAATTTCAAAAACTTTTT
TCGTAGCAATTGGAGCAATTTTTTCAGCAACAGACTCTGTTTGCACTTTGCAGGTATGGATTTCCTCCAAACGTTGCTGCTGTACTGTGGTAAAATGG
ATAATTGTTGAGATAGCAACTTGTGTAAACTGAATGTTATGAGTTTAATTCTCAGGTGCTTAATCAGGACGAGACGCCTCGACTCTATAGTCTAGTC
TTTGGGGAAGGGGTGGTAAATGATGCAACATCTGTGGTGCTCTTTAATGCGATCCAGAAGTTGGACCTCTCCCACATCAATTCAAGGGCTGCTTTAG
TGTTCACTGGAAATTTTCTTTACCTGTTTCTTGCAAGCACTTTCCTGGGAGTTTTGGTAAGTAGCACACTTTCTTGCTTTATGTTTTACGCGCATTTGC
CAATCCTTTCTACGTTTGTCCTCTAGAAGTTTGGTCCGTGTCTTAGTCCCAGTGTGGCTGGTCATCCTTCGTAAACTATTTTCTCACCGACGAGCTAAT
AAGACATGAGTCCCTCTTCGGCAGACTTCTCCTTTTTCTCATCAGGCCACGGGGTACTAACAGTCGTTTCACGCTGTTGTTGTTTGCCATCCATGAGA
ACAGAATTTACTATCCGATTTGCATGTGTTAAGCATGCCGCAATTGTTCATCCTGTGCCAAGATCAAATTCTCCATAAGATTTATTGTTGCATTACCT
ATAGCTTCCTTATTCGTTCGGCTCCACAACTTTGTGGAAATGGATCCCATTTCTCTTTTCAGTGATGCCAAAACAGGTGACATTATTTGACTTGTTGT
AAACTTCCTCTCTTTTTTGGTTGTATAAGAATTTGTATTCATTGGTATGACATCTACTATTTCCTCTGTAATGTCTTTGTATTTTCCTGTTTGAGCAAGT
CGGAATTTTTTTATCTCTTAAGCAATGCTGTTAGAACTAGGTCATTTTGTTCTTCTTTTTTTCCAACTCCCTCGTTTGATCAGATTATGCTAACCATCA
ATCATTATTTATGACATCTTGCTCTTGA

>PG0012168

AAAATTGGCATTTGAGCGTGGAAAATTATATTATTTATATGTGATTAAAGTTATTTTATTTTTATATTTATATATTATAGACGTTGAACTCCTGTTGCC
TTTTTTTTTGGTGTGTGTTTAGTTGTTTAATATTATGAAACTCTTAGTGAAAATTCAGACTTTGTGATGATTTTTAAAATTTTGTTTAAATTATACCTGT
CAACTTCAAGCCTTTTTTTTGGGTTTATTCGATTTGTGGAAGAATTGTAGTAGGGTGGTTGGTGGTGATTTTTGGATATTTTGCGTATTTTGTAAAGG
ATATTAAATGAGAAAGTTGGCAGAGTTTCGTGGAAATAACACGTTTTAGACTGCAGTTGTGCAGATTTTGGAATTGATTTTTTTAAAATTTTTGTTGA
CTATGACAACAGTCACGACTATAAACAGATTAGTGATTATGGGAGGGAGGGTGGTATGCTCTGCTATCTAGCGAGCCATTATTTTACTGTTTTGTTG
TAAAGTTTTTTGGGACTTTTTTGTGTAGTTTTTGTGATATCAGCATTTGGCTACATGTCAAATGAGTTTTTATGGTGATATAGGTGGTTCATTTATGGA
GTTTTGTTTCTTTCTCATATGGTTTTTGCTATCATTAATTTGTTATGGATTTGAACAAAAGTTGCATGGGTTGTTAGCGCCTTGACTAGTTCCATAGGG
TACCTGTTACCTTTCATCAACAGGTCAACTATGTCCACCATGGCTCGAACAAATGAAAACTTACATTGTTTTATTTTCTATTACTCATTGATTTGATA
GTGATTTCATTTTACACTAAAAAGTTCATCCTTGTTGCTGTCACACTATTACGACATGACGAAAATTTAGTATTAGGAAATTTTGTTGTGTATATTAG
TAGCAGTGTTCATCTTTTTAACGTGAGAAAACAGCTTTTTAAAGAGTTTCCAGATTCCTTAAAAAGAATAAAATAGATGTCATTGTTTCTTTGTTCCT
TTTTCAAGCATTATGTGGCACCATAATGTGTGGTTCTTCTGAAAGTTGTTTATTTAAAATTTGTACTTTAGCTTGATGCTTTGGGTCCCCTGTAAAGAG
AAGGATTTTTGATATTAGTATATGTTCTTTTGTCAAGTATCATAGTTGCGTGGGACATAAAATAATATTAGTAATATGTTTCACCAACCCCTTTTTTA
AGTATTGATTTCTATTGCATTTGAAGTACGTGCTCCTGGTGTTGAGGTCTGGGAGGATTGAGTGTACGCAGAGTGTATGTGCGAGCATTGTGGATAA
TTAGAGTTACTTTGTCACATGGGATATTATGTAAATCTATGGCCTTAAGGGATTATAATTTAGAGTTCTTATTTATAATTTGTTTATCACATGGATAT
GTTCTGTATAAAGTAATTCTACATAGTCTGATTGGTTTTGGACATGATCATGAAATGCAGGTTTTGAAGGTTTTGGCTGTTTCTTGTTGATTTGGAGT
TGTTTTATATAAGGACTAAGGCCAGC

>PG0012169

NNNNNNNNNNNNNNNNNNNNNNNNNNNNNNNNNNNNNNNNNNNNNNNNNNNNNNNNNNNNNNNNNNNNNNNNNNNNNNNNNNNNNNNNNNNNNNNNNNNN
NNNNNNNNNNNNNNNNNNNNNNNNNNNNNNNNNNNNNNNNNNNNNNNNNNNNNNNNNNNNNNNNNNNNNNNNNNNNNNNNNNNNNNNNNNNNNNNNNNNN
NNNNNNNNNNNNNNNNNNNNNNNNNNNNNNNNNNNNNNNNNNNNNNNNNNNNNNNNNNNNNNNNNNNNNNNNNNNNNNNNNNNNNNNNNNNNNNNNNNNN
NNNNNNNNNNNNNNNNNNNNNNNNNNNNNNNNNNNNNNNNNNNNNNNNNNNNNNNNNNNNNNNNNNNNNNNNNNNNNNNNNNNNNNNNNNNNNNNNNNNN
NNNNNNNNNNNNNNNNNNNNNNNNNNNNNNNNNNNNNNNNNNNNNNNNNNNNNNNNNNNNNNNNNNNNNNNNNTTCGGTTCGGTTATTTCTTCGGGTTTTTC
GAATAAAACCATAACCAAACCAAATACTATCGGTTTTCAAAAATCTAAAACCAAACCAAACCAAACCCAAATAAAATCGGTTTTATTTTATCGGTTT
GGTTTGATTTTTCGGTTTGGATCGGTTTTTATCCAAACCGTGAACACCCTATGTAGGAGTATCAAATGAGCAGGTTATATATAAATTTAAACAGATTA
AAATGATTTAGGTTGATAAATAAATGAATAAATTAACTTGTTTAGAAAGTTATTTGAATTGATTTTAAAAAAAAGAAGAAGTTATAATTAACTCAAT
TTGTTCCATTCTTATTAAGTCTTGATTGTTTTATAATTTTTGAAGTTCCTAATATTTTTCTCTCTCTTAAGTAGTACTTTTTTTTGTATAATATTTTGTGA
CATTTTAGATTGAACATACTCCTTAAAAATGTCACTCACCCCTAAAAGATTTATTTTTAGTAAATTATCCTTAATTAAATAGTAAGTGCCTATTTAAT
ATTATTCACACTCACTTATCTAAATAAGGATATAAATTTAAAAAAAAGTGAACAATTCTTCTTCACTATGTAAATATTATTTATTTAGCTTAAATATA
AAACTTAAATTATCACATAGTAATATATTTATTTTTAAACTGCATGTCTTTTTTTTAAAAAAAAAGTTATTCAAAACTAATTAAACATATCTTGCTCGGG
AAAGAAAAATAAATAAATGTACCTGGCACTCTCTCTATTCTATAAAAATATAAATTTTCTCCATGCACATATATTTGTGCTAGGGATCTAAAAAGTC
AAAAAAAAATATTTAAAATTCAATCTAGGAAGTTAATTTATTTTTTTTAGCAAAAATTATTCTTTCCCTCTAATTTTGTGACTGAAATCCAAATCAGCA
TTGAATTTCATTAATCCTATCTATATAAGGGCAAAAAAAAAAAAGACTTTTTTTCTTCATCTGACTGTTTTTTTGTTTTCTTGAAAATGTTGAAAATTTG
CAGAAATTGAAGATAAAGATTGAATCTTTTTGAGTTTTGATTAAGTGGGGTTGTTTTATT

>PG0009954

GGTTTTCGTAGAAATGAATGAATTTTTTATTTATTTTTTTCATTTAGTATGTGTAGACATTATTTTAATAATATCTGTATATAATATTATTTAATAAAAT
AATAAATTTCATGAAAGTTCATTGGTAGGACTACTAATTCTACATACTTATTTCTTTTATTTTTGATTAAATGAAATATATCTAATAAAACACTTTATC
ATATATTCAGTTATCTAATTTAAGTAAATCATAATTTAACGGTCTATTAGAGTCTATTTGACTTTACTGTTGAGAAGTCAAAAACACTAATTTTGAGT
AAAAAACATTTATTTTGGAAAAACAAGGTGTTTCACTAATCAATAAAATTGCTTTTAAATGAAAGCAACAGTGCTAAAAATTTCTGCTTATTAAACA
AAAGTAGAAATAAAAGCAGAAAATTATTTATTTCAAGACCAAATATATCTGTCATATATACATATTTATCAATATATCTCTTATATTTATTTATCTATT
TTTTATTTTTTGTGATAAGATTTTTGTACGTAGTGATTTGATTTGTGAAATGATTTTTAATTTATTTTACTTGATATTTCTAATTATATTTAAATCATCA
TCTTAATATTATATTAATATTATTTTTTTATTCATCTATATATAATATTAATTTAAAATCTAAATATCTGTATTTTAATTTAATTTTTTTTATAATAGATAT
TTAAAATAATTTTAATATTAAAAGTGCATTAATACTTGTTCTTTTTCATAATTTGATTCTCAAAAGTATTTTTTCTTAAAATTAAGCCAAACACAATCC
GCTTATCAAAAATACTTTTCAAATGAATTAACCAAATACAAATTGTATTTTTTCTCAAAAACACATTTTTGAAAAACTATTTGGAAAAAATATTTCTA

AAAATAAGCAATTTTTAATAGCAATGTCAAATACGTTGTAAGTTTAGTTAGTCAAATAGAGAGATATTTTTAAGCTGTCAATAATTTAGAGATAAAA
TTAATAATTTACGTCAAATTCAATGGTGTTTTCAGTACTTTATGAATAAATCAAAGTTTAGTGTCTAAATAGAATTTGGTTTATAAATTTGAGGGGCT
CTACATCCTACACATGGTCACGTGAAAACATCCCCCAACTGTCTTAATCATCCTCATTAAGCCTTAAATGATAATCTTCCAATTTTGTCGAGTAATTC
AACTGTCCGGTTAAAATCTAACCATGGTTACTTTTTAACTTATCTTCCAACTGTCTATTTTTCTCCACAAGAAAATTCAATCAATGTCCCTCTTCTCTT
GCATTTCAACACACAGTATGGTGTGTCAGTGTGTGTTGTATATATAAATAAATAAAAAACAATGTACAGTGTAGTACTATCAACTTACTTACTTCTG
CTACCGAGTAGAGTTTGTTGTTGTACACCCTGTAGAAACTGACAAACCGACACCTAAAACACTACTCTTAATCCACTGCTTTCCGCCGGCGCGGTCG
CCGTCACCGTCGTCGCAGTGCA

>PG0030154

TTCGTCCTTAGACTTAGGATTCGGCATATCCGTGCTTACCGATGAGATGTTTTTGGCACTAGGTGCTAGTGGGACATCTCCTGCCACTTTGTTGGGAG
TGATATTCCCTTTAGGATTTGAATTCCTTTTTGGACAAGTGTCCAAATATTCACACAAGAGTAAAAATTCACTCATGTTTTCCGTTTGTTCAGTATTG
AGTTGTTGTATCCCATACTGCATCACTTAGAAGAAGAATGCTCCAAATTGCCAAAACTCAGATTACCCGATGAAAAGGATAATCTAATTCTACAAAC
AAATGCCTCCGACTATCATTGGGGTGCATTATTACATACAGATTTAAATGAAATCTGTAGATATACTAGCGGAACATTCAATGAAGCTGAAGTAAA
ATATTTAACCAACGAAAAGGAATTACTAGCAATTGTCAGAGGGATAAGAAAATTTTCTACTTTTCTTTTACCAAAACAATTCACTATCAGAACCGAC
AATACTCAAGTATCAGGATTAATATTCAACAAACTTCCGTCTGAACCTCAGTACAGAAGATTACATCGATGGCTGGTTCTATCGTCATTTTACTCTTT
TAAAATTGAATACATTAAAGGAAGTGACAATTTTCTTGCAGATAAACGTTTGAGAATAAAATATAATGCTTTAATTCCCAAAGCTCGTCTTGTTTGT
ATCCATTTTTTATTTAGGTGTGGAAGTTTTTTATCAACA
CTTGTTGCTTTAACGTTGAATTCTCTTAATTTTTCCACTATGATATAATTAATCGTTGAAAAATATAATTGCAAGTCTTTGCTTTCCATGCTGATGCTA
AAGAATCTGCAGTAGACTCCTTCACCTTGTGCATAATCTTTTATTGAATTTCTCAATTGTTCTGGTAGCTTGGATATTTCCTATAAATTCGGTTTAGTG
TAAACTCGGTCTAGGTATCCAAATTCAAATAATTGGGCCGTGAAACCCGATGATCCGTTAGTAAATATGAAAATTCTTGAGAACAGGCTGGTTGTCC
CTAAAACTCCAAATGTTTCTCCGGTTTTTTTTTTTCTTCATAACATTATCTTACATACAAACTATATATATATTTGTATGTTGGATTTGACAACAATTCAA
GTTTTTTTTTTTTTGTCAAATAACAACCTCAAAAAAATTAAAAAATGTTTTAAGCTTCCAAATTGAGCGGAAAACCATTCACTTTGAACAAAGCTTTG
TTGGTAAAGAAACAAGTTTTTACTATTTTTCACATGGTGACAACCATATCTTTAGTTTTACTCATGTTCCATTTTATACATATCTCCATTTGTTGTGGT
GGCTAAATATCAAAATAGGGTTGTAGCCAGGGTTGTTCCCTGTCCTGGCACAAGACTAGACAAAACAAAAAAAGGGTGTTCAAGAAAAATAATGTA
CAAATTTTGATTGTACAATTTTTGTGATTTAAG
AGGGAAAAAATCTTTTTGTTGTCGTTATTGTTGTTGTTGTTGAACATTAGAAAGGCAAAA

>PG0035252

GATTCCTCTATCTTTAATTTGAGGTCTCAAATTTGAATATGAAAAAATTCCTAACAAGAAGTGTTTCCACTTTTAATGAATTCTACAATGTATTTGGT
ATAGTAGAAATTATAATAGACAAATCTCGCTGGTCTTTCTCTCACTCCTATGCATTTGGTATTTTCTAGATACATGTGGATCACACTAGATACATACG
GTGATACAGATATATGTATCTAGCGTGATTTACATATATCTGAAATACACAACTAATAACAAATATACATGTATCTAGGTGTATCTTCTTCAGTATAT
GATAAAGTACGTAATTATTTGAAAAATATAAATATAACTAATATTATATATACTTTCTTCATTTCAGAATAAATGAATTATTGAATCTTTTTTTATAAT
TCAAAATAAGTGAATTGTTCAAAGTTTAAAAGAATCGATTGGAAGTTTTTCCAATCATGAGCATGCCAAATTCATGGCTCTATAAAAATGGAAGGG
GGTTGTGTTAATTAACTTAGCCATCAATTCCACACTCTTTGGGAAAGAGAAAGTTTCTCTCTCATCTCCTTTGTCTTTTTTCGTTTTTCCTTTTCATAAT
AAATTAATCTGCTCAATTCCTCTTAAATTTTAAGAAAAAATATTGTGCAGATTCAACTTTCTCCAGCATTCAGGTTTCCCTTTTTCCCTTCCTCTCTCT
CTATATATCGTATATATATAAGATATATTTAGTACAAGAACGATTTTTTTTTTAGAAAATTAAAGAAATTTCTGACTTATTTTCTAGTATATTAAGTA
AGCAAAAAAATATTAATAATATGTAAACTAGCAAAACATTATAGAGATGAAATGAGGTGGAAAGTGGGGGTCCGGGAATGACATGGAACGAGATG
GTCAAAGGGTGAGGTTCTGATCGTTAGATAATTATTATTTTTTGAAATTGACTAAAAATAAAATAATTACACAAAAATTGGGACAAATGGTAAAAA
AAATAAATCAGAATAATAATATTTACAATTATTTTGCACGTCATGAAATTGAATTTGCTTAGTAAAGACAAAAAAAACTTACGATAAATACACCATA
TGTTATTTACTCTTTTGCCCCTGCTTTTTCCTTAAAAGTCTATAATTACTTAAGTTTTTTATATTAAGAAACCAAAATTGTTTAGGATTAGATATTAGT
TTGTTAATTTATTTGATCCATATCTAACTAGGATTTGCGCTTCAAAGTTATATAAAAAAAAATAGGAAATATGTTTTTCAATATCTAGTCAAACTAGGT
TTCGTACTATTATAAATAGAGTCATTACTAATTAAATTCATATAGTAAAAAAGACATACTAAACAATTTATCGTAGTTTTATGAAATAAAATATCTTC
CTTCGTTTATTTTTCTTGAATACTGTTTCAATAGAGCCTTGTTAACATCAATAAAACATATTGTGACTTATAGACGACGTTTTTATTAAATCTTGAATC
TGTTTTTCACAGTGACGAACAATTTTACAACA

>PG0004171

AAAGCGCACACATTGTATGAGTGGTTTATTAATCATGGTACAATTGAATATAATTTTATTCGTTGATTATATAAAGAAAAATCAATTGTAAATTTGA
AAATTAATACTTAATCCAATTTACTAAATCGTGTATTACCGAGTTGTTAATGTAATTTTTAATTGGAGAATAATCTACTTATATCTATTATTAACATG
AATTTAATTTTCTCCTTTTGATAGAAAATTCTATATTAAATAAAATAATTTTAAAAAAAATGTATATTCTGATTGTGTTATACATTAATTACATACATTA
AATATATTTTAATTCAAGTATCCTACATAAAATACATCTATTTTGAAGTATTATAATTGATCATTAGGTATTGATTGATTTTTTATTTGTTTGTTTTGA
AACATCCACTTAATTAGTATTATATGAGTAACTGGTTTTTTTTATTCATGATCAAAATTAGTGAAATAATTTTACCAATTTTAATATATGTTAATTATT
TTTTGGAATGATTATAATATAGAAGATTAATGTGTCATTCATGACATAGTTATAAATGTCTTATAATCAGTTTTTTTTTTTTATAAACATCTAATTTCC
AAATTTACAATGCTTTTTCTCTTGAAAAACATTCATTATGTAGACTAATATATTTAGTGGATTATATTTAAAGAAAAATTCAATTAAAACTTTTTGAA
TCTACCCTTTATTACTATATTTTACTAAATTTGTATTATAAAATAATTGATTATAATATTAAGTAAAATAAGGTAGTCAGTTTTAGTTATTATTAATGA
GAAATTTAATTGTCATCTTCAATAAAGAGATTTATTTTAAGCAAAAAAAATACTTTTTTATTTTAAGCAAAAAAAATACTTTCTCAAATGTATGCATTGG
TACAATATGAACACAATTGAGTTTGGGGCAAGGGGTAGAGGAAGGAGTGTAGGTTATGTATATGTACAACATAATCTGTATGGTATAGTAATGAAA
AAAATATATTAATGTATAAAAACGAAATTACAAATTTTTTTTTTAACTAAAGCGTACAATCTTTTTTTAATTAACGGAACAATATTTATTTTTAAGAA
TAAATCGAATGAACTATATACACACAACAAAATATATACACAAAAATACTTTTTATTTCCCTTGGTTGCCAAAGAATTAGTATTTTTGTAGTTCAAG
GAGTTTGGAAAATAGAAAAAAGTTACTTTTATTTTAGAGGGAAAAGTATTGAGAAAATAAATGCAAAAAATTGAAATATGGATATTATAGACTTTTT
AGCATTACTCTTTATAAAAACTATAAAAATAAAAAAGATTTTGTGGTAAGCAGAATTTAGTTATTTTGTTGAGAATGGCTTAAGTAACAAATTTTAG
TAAAATTAATGGCATAGAGATAAAAAATGAAAATGAAGTGTGTTGGCCCTTTTTAACGCATCTCCGATGATTAGTAGGATGCAATATTGATAGGGTT
TTCTAATGAATTGAATGGGAAAAAAAGAGAATC

>PG0008849

CGTTGTATTACATTATTTTTATGTGAACACTAAAATAGTGATGAAATATTCGGTGAATATGAAAGTGATGATATGGTTATTGATGAAAATGATGAAC
AATCGGCTCAGAGTAACAAAGTCATGTGCTTTGTCTACTTCACGATGTATGAAATGATGCTTGTTTCACGCACTCCAAACTACCACATTGCTCCAGT
GTCATGGACACTACTTGTTATTTGTTGCAACGAAGATCCAATTGACTTGTAATACAAACTTATGGTTAGTTTTGATAGTTTTTAAAACTTATGGATAT
AAATCATATTTTTCTAAAAAAGTGAAATATGTTTCTCAAATACTATGACCAAACACAAGGTGAAATTTCACCCAAATAATATTTGCCAAGAATATTT
AAAAATTTATGATCAAACGCTAGCTAAATTATTTCACTCTCAATTTCGAATAATGCCACGTAAATTTGAAAAGACAGTAGTAATCAACTATGTACAA
CACGTGTAACAGTTAAATACCACTAAATATGTCCATTACATCACAATTGCATAAAAATACTATTGTAGTACAGTAAAATAAATAATTATGTATCTCA
TTTTAATATTAATTTAACTAAAAGAAGTGACATGACAACTGAACTTACCTTTGATTTTCCGAAAACATTTTTTTCCTTTTTTTACCCAATATTTAATGA
ATTTGAAAATAATGAAAATTTTAGAAAAAAAATATTTTTTATATAGCATTTCTATAATTTTCTCATTGTTACAGGTTGGTTATTTGAATACTAATTTAG
CTTATGATGAATTTAAAGCTGCAACTAAAAAAATGTTTTACAATGATCCAATAATAAATTACATTAATAACTGGTCATTTTAATCCTTAAATTATAATT
TATAATATAATATAAACTAATGTGACAATGATATACCACTAAATATATATTTCAGTATATTGCATTGTACAAAAAAACCCACCCAATGAAAATAATAA
TTAGATTCTTCTACTATGGTCGAGGAGATTAATTTGGAGGATACATTGGTACGCGGATTCATGATTTAAATTTTTAATATTGAACTTATTAGGTTGCT
CGTTTCTTAATCAGAGGTTTTGAGTTCGAATTTTAAGTATAAAAAAATTCTTACTAGGGAGCACTTCCCTTCGAATGAGGCTCTACGCAAAGCGAAT
TCAAATTAGTCGGGCTCTAATGGAGGTACCAGACACCAAATAAAAACAAAAACAAAATTAAAAAAAACAAATATTGAATCTATTATATTTTTAAAA
TTATGAATTTAACTTTTGTTACAATTTTAATAAATTATTATGCATAAATTTATGCTTCACGTCGAAAATACTAAATCGATGAATCCGGGGTCTTTCTCT
CCACTATAAATACCCCTCTCTTTGGCTTAGTTTCAATATAACCAAAAACTTGAACTTAGGAAAATACACATTGTGTATTATATAGTTCATTATAAGGT
TATTTTTGAAGAATTAATATAATTCTCATAATTAATT

>PG0034953

TTTAACATATATGCACGTTCCAATACATGTTCATTGTCAAATCTGTACATATACATTGAATACCCTGCATGAGTTCTCCTTTTGACTAAATAAGGTCC
ATATGTTATTTCAGCTCCTATGCATTTCACTAATTGTGTAACTACTCTCTACTTTGTTGTTCTTGCATTATATGATTATTACATGTCACTTGTTGACTAC
ATAGTCCCACAACTTAAAAAATGCTATGACCACCAAAAGAATCATCTCAAATGTTAATGGAAAGATATTAAATGAATCTGCTTATTGATGGGTGGT
ATCCTAGGGGTGGAAAAATCACCTAGCATGGGTTGATCCCAATATTACAGGTGGTATCCCAGGGGTGGAAGAATCACCTAGCATGGGTTGATCCCA
ATATTACAGGTGGTATTCCAGGGGTTAAAAAAAAGCCTAGCATGGATCGATCCCGATTTATATGTCTACCACTGATCAACTGGTCATTTGTATTATA
TGTTGTGCATAGAGTGTAAAGTAAAGAATGATAAAGTAAAGAAAAGAATGTAAAGTAGCTGATTCCATAATATGAGAAAAGGTGGTCTTGTATTCT
TACATTGGTATATGATTTCATTTGAGTTCTTATTTCACATACAGTACTTTATGCCTTACATACTTAGTACATTCTCCCGTACTGATGTCTCTCTTGGGG
AACCTGCAATTCATGCTGCAGGTACAGGAGCTAGTAGATCTCCCCAATAGGAGAACAGGACATCCAACGGCTATTGGTGAGCTCCAGGTTGATTCG
GGGCTTTCCGAGTCTATAGCATGTATTTTGGTATTGTATAGTAGCTGAGTGTAAGGCGGGGCCTGTCCCGACCTTAACACCATAATGATTTATCATTT

AGAGGCTTTGTAGACTATGTATAGTGAAGTGTGGTTGCGCCTCGTGTCTTAGACATCATGGTCCCAACGGCCAAGTCATGAGCACTTTCTTTTGGCTT
GTTAATACTGTCTACTTTCATGAAAACATGTTATCCACTATGGCAGCACCTTTAAATGTTCATAGATAGAGCATGATTACAGGTTGGTCCTCCCGAGC
CTTCTCGGCAACGGGTGCCGGTCAGCCTTAATGGGATTTTGGGCGTGATAAACTTTTCACCTGGCATATTTAAAATCACAAGATTAAAACGTCTTTTT
TTTTTTCTTAAACTTCGTTTCAAGTTAAATTAGGTCATTCTTTTTTAAATGAATTGAGTAATACAAGTAAAGAGGGACGGAGGGAGTAACAAAAAAA
CAATATATAAAGGTACAACAAAGAAGAATAACACATGGCCACGTGTATTTGTTGCATGATAATTTCGTCTTCTTCCCCAAACGTGTTAATTTCCCCA
GCAAGCACCGCAAATTTTTTGTTTTCTTATTAGCAGCTAAAACGTTCATTCATACATATCATGCAGCAAAAATATTGCTTAGAGATAGAAAAACCCT
CAAATGATGTTAAATTTGATTCAGAATTGAGTTTTAAAA

>PG0005009

ATATAACATTGATGAATCAACAGTTTAATAGATAACACAAGTACGCTTTTTAAAAAAAAAAATTGATGACATATAACTCATTCCCCTATTAATTAATA
TTCTTTCCGTTACATATTAGTTGATCAATTTGCATTTTGCATGATTTCACCCTTAAAAAACTTCTTTAGAATTTTCCAACCAATTATGAACACTTTCAA
AAAAGAATTAATTGTAATATAAAATAAAGAAAAGTTAATTAGTGCGTTCTTGATTTAATAAGATAAATAATTAATATGAGATAATTATTTTTTCGTAT
AATAATCAATTGATATGTAACAGAAGAAAGTATAGATATTATGATAAAATATTTATATTAATTATTAAACTTTAAAAGAAACGTGGAAAACTCAAAA
TGAACAAGTAAAACAGAAGAGAACGAGTATCTTGTACTGTCAGATTGTACTTTCATTCATTCTCGGACTTTTAAACTGTTTTGGGGACTGAAAGCTA
CTTGTTCACACACCAATCTCTTTCAAAAATTATACAATTTTGGTGTTTATTTTTCATTCTTATTTGTTTGCAAATCTCGCAATCAATTAAACAAAGGTT
ACAAGGTATGATTTTTTTCTATTCGTTTTCTTCGTATAGCTCACTACCCTAATATTTTTGTTTAATTTGGTATATTTGATAATAATAAAAAAAAACGAG
TCATGAGTCTGATGAACAAGCAACTGATATAGATCCTTTGATCTGCATATTCTTTTTCCGAATTTCGATGAGTATTATGTGTTTTGTGCAAAAAATTAT
AATCTTTTTATTGATAACAACGTACTCGGTATAGTTTCATAAGTGCGGTCCGGAGAAGAAGGTAGGTTATACTCTTTACCTTTTTGTAAGGTAGAGA
ATTTGTTTATGATAGATTCTCGGCTCAAGAAAATCCTTTTTCGAGACATTCCAACTAGAATTACTTGTTCCATTGAATTTGTAGGGATTGGTGCTGAT
TGAATTTGTACTCTAATTCTAAAAGAGGTATATGCATATATTTGTAGCTGTGGTATCTGTGTCAATTTGTGGACATCTCGACTCATCTAGTAACAGAT
TACTACTGCTCAAAAGTACATTTGTAATGTAAATCTGTGTACCAAGGTTTGGGTAGATAGGCTCACTTTAAGTGTTGTAGTCAGCGGTGGTTTAGCG
TTATATCTATGGGTTTACGTGAACTCAGTAGCTTTTGCTCAAGCTCTATATTTCTAGTATAGGAACCTATAAACTCGAGGTCGTTGTAGGAACTCATA
AACTTTAAATCTTGGATCCACCTCTGTGGTTGTAGCAGAGCAATCCTGGATGCTTTTTACCACTCAAACATCCCTTGGGAATATTGAACTTATTTACC
TCATCGTTCTACTAAAGATTTTCATGTTGATCCTTTTCATAATTTTGAGTTGAAATTGGAAGTGTAATAGCTATCTTGTTTTACAGTATTGTTGTCACA
GGACTAGTTTGGAGAGGCATCCAGTGAAACT

>PG0014998

GATGAATTTTTTTCTTATATAAATGGACATTTAGCATATAACTAATTCTCACTATTTAGATGAAACAATACAATATGTCGTAGGTGTAAACTTACCAG
GTATGTAACAAAAATTCAGCCTTTCATGTTTAAATATGAGTCATTTAGTTATTATTTCTAAAAAAATTATTGTGCTTTTTATGTTGTGCTTCTCACATG
AATGAAAAAATAAGATAATTTTGTTTGGAATGCTAAGAATATTGTCGTCATATATAGTTTCTCAAGCTTTTGGTATCTCAGTAATACTCAATAGTGTA
GCAATATTTATCGTCTTCTTATTTAGCTATCTCGAAGAGGTAAGCATGGAAAAGTTGGTCCAATTAACTTCTTAAATTGTATTTGTGTTATATACAAC
TTCTCTTGATTTGTTTCTACTCATCATTACATTTTAAAGTACAGTTGTGTAGGAATCTTGCAGATATCACTATGTACATATTTCAAAATGATTATGAAT
GTCGAACCTTTATGACTTTGTTTTCTTTGATAATATCTCTAATTTGTTGATAATGATCATTTCTTAGAAATGATCTTGCCTCTATAATTTGCCTTTTGAC
TTCTATATGTTGGATACCTTTAAAAGGGGGATTAATTCAATAATATTTAATTTACGATATGCAAATAAGTCATAAAGGAACGAGAGGAATCATCTAT
TGATTTTGGATCTCTAATACATAATTATAAGAGCTATTAGACTAAAAACATTCTATCTTCACAAATATTAACCTCACCTTCTATCCAACCTGAGATCA
AAGGCATGTGACAACTATCGTCTCCTTTATAATTGTTCAATAATTTAGTGATATGACATTATTTTGTTTTATTCCAACTACATAATAATCAAGTCTTCA
CAATTTATGGATAATAAGGTTGTCAAAAAACTGACTTATAATTTAATTGTTCTTGCGAAGGTTTAGTATGGATTACACGTGCAACACACATCCAGAA
CTAGTTATATGATATAAGTGAGTTTGTCTTTAATCAAAGTAGTGCTTATTTGTTTTGTTAGGGATATTTTGGTATTTCAACTTTCAACTTTTTTTATTCC
CACTTATAGTGATATATATATATATTTACTTTTTTGTAATAGATTTATTTTTACAAGGTACTTTTATTTTTCTTTCCGGATAAGAGATGTGAAAATAAATA
ATCTGATTTCCTCCCAATTGCCATAGTTACTACTTACTACACGACCAAATTATGGGCATGATGGAAACATCATTTACATTAAAATATAAAGTAACAT
AATAAAATTGGAAAGATAGGATGAAACTGAATTAATGTAACACACATAAAAATAACTGATCATCATGAGTGACGTCCAGTTTCCTTAAAATTCTGC
AAACTTGTTTTAGGCCTAGACATGTCGAAAAGCTAAGAGAAAAACCCTTTTGAATATGTTCCCAATGTGGAGGTGGAGGCGGAGGCATACAAAATA
GGAATAAGAATAGAAATAAAAATAGAAAA

Supplementary Table 4 ： The RNA concentration of StNHXs

Nucleic Acid Conc.	Unit	A260	A280	A260/A280	A260/A230	type
560.7	ng/μl	14.018	6.708	2.09	1.99	RNA
561.8	ng/μl	14.045	6.803	2.06	1.96	RNA
540.9	ng/μl	13.522	6.436	2.1	2.02	RNA
628.1	ng/μl	15.703	7.574	2.07	2	RNA
529.2	ng/μl	13.23	6.325	2.09	2.01	RNA
527.7	ng/μl	13.192	6.313	2.09	2.01	RNA
557	ng/μl	13.926	6.687	2.08	1.98	RNA
430.4	ng/μl	10.759	5.215	2.06	1.9	RNA
1131.2	ng/μl	28.28	12.885	2.19	2.2	RNA
1173.4	ng/μl	29.335	13.518	2.17	2.17	RNA
540	ng/μl	13.501	6.45	2.09	2.02	RNA
227.5	ng/μl	5.689	2.854	1.99	1.66	RNA
527	ng/μl	13.175	6.328	2.08	2	RNA
535	ng/μl	13.376	6.42	2.08	2	RNA
522.5	ng/μl	13.062	6.252	2.09	2.01	RNA
551.5	ng/μl	13.788	6.584	2.09	2.01	RNA
543.5	ng/μl	13.588	6.488	2.09	2	RNA
586.6	ng/μl	14.664	7.067	2.08	1.97	RNA
453.2	ng/μl	11.33	5.489	2.06	1.91	RNA
437.6	ng/μl	10.941	5.26	2.08	1.92	RNA
524.5	ng/μl	13.112	6.288	2.09	2.02	RNA
1395.6	ng/μl	34.89	16.411	2.13	2.03	RNA
1722.8	ng/μl	43.07	20.237	2.13	2.01	RNA
840.7	ng/μl	21.017	9.936	2.12	1.98	RNA
891.3	ng/μl	22.283	10.548	2.11	1.96	RNA
754.6	ng/μl	18.864	8.961	2.11	1.96	RNA
529.2	ng/μl	13.23	6.361	2.08	2	RNA

249.2	ng/µl	6.23	3.12	2	1.68	RNA
193	ng/µl	4.825	2.423	1.99	1.68	RNA
627.8	ng/µl	15.695	7.592	2.07	2.01	RNA
801.4	ng/µl	20.035	10.415	1.92	1.86	RNA
823.1	ng/µl	20.577	9.64	2.13	2.1	RNA
937.5	ng/µl	23.436	11.03	2.12	2.1	RNA
969.5	ng/µl	24.238	11.396	2.13	2.1	RNA
943.5	ng/µl	23.588	11.057	2.13	2.1	RNA
876.6	ng/µl	21.916	10.325	2.12	2.05	RNA
826.9	ng/µl	20.672	9.715	2.13	2.05	RNA
805.7	ng/µl	20.144	9.449	2.13	2.02	RNA
663.3	ng/µl	16.582	8.275	2	1.86	RNA
619.6	ng/µl	15.49	7.636	2.03	1.87	RNA
570.4	ng/µl	14.259	6.89	2.07	1.97	RNA
569.2	ng/µl	14.23	6.896	2.06	1.93	RNA
